# Clinical guidelines for managing hearing loss as a complication of drug-resistant tuberculosis treatment: an evaluation of implementation fidelity in Kano, Nigeria

**DOI:** 10.1186/s12913-022-07536-y

**Published:** 2022-02-03

**Authors:** Sani Ibrahim Muhammad, Ejemai Amaize Eboreime, Vivian Ifeoma Ogbonna, Iliyasu Zubairu, Latifat Ibisomi

**Affiliations:** 1 Division of Epidemiology and Biostatistics, School of Public Health, Faculty of Health Sciences, University of the Witwatersrand, Johannesburg, South Africa; 2 Department of Otorhinolaryngology-Head and Neck Surgery, Aminu Kano Teaching Hospital, No. 2 Hospital Road, off Zaria Road, PMB, Kano, 3452 Nigeria; 3 Global Mental Health Research Group, Department of Psychiatry, University of Alberta, Edmonton, AB Canada; 4 Department of Community Medicine, University of Port Harcourt Teaching Hospital, Port Harcourt, Nigeria; 5 Department of Community Medicine, Bayero University Kano, Kano, Nigeria; 6 Nigerian Institute of Medical Research, Lagos, Nigeria

**Keywords:** Adherence, Drug Resistance, Ototoxicity, Tuberculosis, Nigeria

## Abstract

**Background:**

Nigeria has a high burden of Tuberculosis (TB) including Drug-resistant Tuberculosis (DR-TB) and hearing loss. Despite several efforts directed toward its control, many patients fail to respond to treatment, having developed DR-TB. Lack of adherence to the DR-TB guidelines/improper implementation of the guideline has been identified as one of the factors impeding on effective treatment. This study sought to measure the implementation fidelity of health workers to management guidelines for hearing loss resulting from DR-TB treatment and to identify its determinants.

**Method:**

A questionnaire-based cross-sectional study was conducted at the Infectious Disease Hospital, Kano. Implementation fidelity of the Programmatic Management guidelines for the treatment of Drug-resistant Tuberculosis was measured under the four domains of content, coverage, duration and frequency. The determinants examined are intervention complexity, facilitation strategies, quality of delivery and participant responsiveness as proposed by the Carroll et al. framework. Other determinants used are age, sex, professional cadre and work experience of healthcare providers.

**Results:**

The Implementation fidelity score ranged from 40 to 64% with a mean of 47.6%. Quality of delivery, intervention complexity, participants’ responsiveness, and being a medical doctor exerted a positive effect on implementation fidelity while facilitation strategy, age and work experience exerted a negative effect on implementation fidelity.

**Conclusion:**

The implementation fidelity of management guidelines for hearing loss resulting from DR-TB treatment was low. Implementation fidelity should be assessed early and at intervals in the course of implementing the Programmatic Management of Drug-resistant Tuberculosis guideline and indeed, in the implementation of any intervention.

**Supplementary Information:**

The online version contains supplementary material available at 10.1186/s12913-022-07536-y.

## Background

Hearing loss is a known complication of second-line drugs used in the management of Drug-Resistant Tuberculosis (DR-TB) [1, 2] . This complication is particularly common with injectable medications such as aminoglycosides, which often cause permanent damage to the hearing and balance systems of humans [3, 4]. Yet these medications remain essential in the management of DR-TB, given the burden of the disease.

Nigeria has the tenth highest prevalence (323 per 100,000 people) of TB globally [5, 6]. Despite several efforts directed toward its control, many patients fail to respond to treatment, having developed DR-TB [7] - defined as resistance to at least one of the anti-TB drugs [8, 9]. In 2017, the prevalence of DR-TB in Nigeria increased from 4.3% to 32% and from 25% to 53% for newly diagnosed and previously treated cases, respectively [10]. This increase in prevalence raises concerns about meeting up with the target of providing access to DR-TB diagnosis to all presumptive DR-TB cases and enrolling 100% of diagnosed DR-TB patients on appropriate treatment, towards ending Tuberculosis by 2035 [10, 11] . Further concerns exist about the rise in the incidence of hearing loss resulting from intensified efforts to curb the rising cases of DR-TB. The incidence of hearing loss secondary to DR-TB management increased from 15.6% in 2004 to 61% in 2016 and may be contributing to poor patient compliance to the treatment regimen [12].

The factors suggested for the rising incidence include (i) limited qualified personnel to implement the management guidelines, (ii) lack of appropriate audiological equipment for screening the patients on injectable second-line anti-TB drugs, (iii) health care personnel’s negative attitude towards patients with hearing loss, and (iv) lack of adherence to the DR-TB guidelines/improper implementation of the guideline [12, 13].

The World Health Organization’s (WHO) revised protocol for managing DR-TB called Programmatic Management of Drug-resistant Tuberculosis (PMDT) published in 2011 emphasizes monitoring and management of adverse effects resulting from the DR-TB treatment regimen [10]. The core components of the suggested management strategy for managing hearing loss in PMDT are listed in sequential order:

1. Develop a management protocol and train all staff responsible for delivering treatment of DR-TB and its implementation.
2. Inform patient about the early symptoms of ototoxicity, such as tinnitus and dizziness.
3. Perform a monthly audiometry of every patient on injectables, starting with a baseline at the time of enrolment on treatment.
4. If the patient is experiencing clinically significant ototoxicity, decrease the dosing frequency of the injectable to two to three times a week. Then, consider switching to capreomycin.
5. Stop the injectable if symptoms worsen despite dose adjustment, and add additional anti-TB drugs to reinforce the regimen

Nigeria adopted the guideline in 2012 (Additional file 1). The management of hearing is a component of the Nigerian TB Guideline [14, 15].

Whereas there are currently nine special centres established in Nigeria to carry out a hearing assessment in the treatment of DR-TB [16]. Empirical evidence is lacking on how well the guidelines are being implemented nor on the factors affecting the implementation process. This study contributes to filling this gap by assessing the implementation fidelity of the management guidelines for hearing loss among healthcare providers at the DR-TB Centre of the Infectious Diseases Hospital, Kano State, Nigeria.

## Methods

### Analytical Framework

**Fig. 1:**
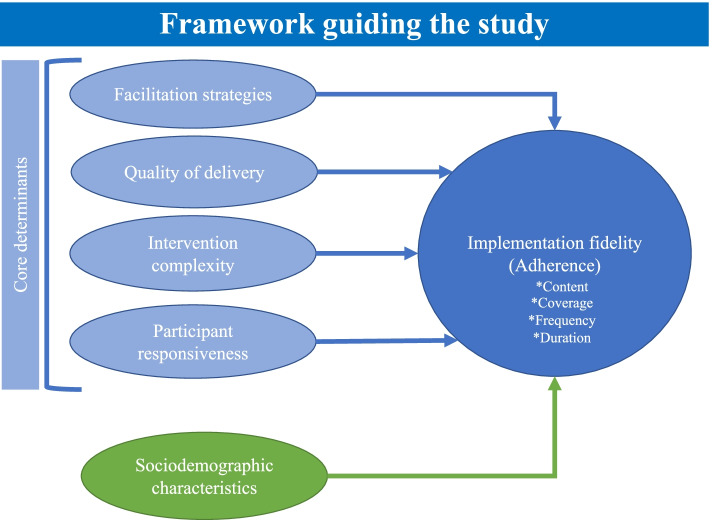
Conceptual framework for implementation fidelity adapted from Carroll et al. (2007)

Implementation fidelity has been defined as “the degree to which programs are implemented as intended by the program developers” [17]. The conceptual framework in Fig. 1 adapted from Carroll et al. guided the assessment of implementation fidelity and its determinants in this study [12]. Implementation fidelity has four constructs of duration, frequency, coverage and content [18]. The four determinants affecting implementation fidelity according to the framework are: intervention complexity, facilitation strategies, quality of delivery and participant responsiveness [19].

Facilitation strategies refer to the delivery mechanism employed towards the effective implementation of an intervention. These strategies include training and the provision of protocols and guidelines. Quality of delivery typically describes the way the health care providers deliver the intervention. In this case, receiving supportive supervision, coaching and mentoring was used as a proxy measure of the quality of delivery of the PMDT management guideline (Additional file 1). Intervention complexity describes the sophistication of an intervention, as simple, detailed intervention are very much likely to be delivered with high adherence (implementation fidelity) as compared to complex, vague interventions. Participants’ responsiveness refers to the extent of participants’ responses to the program or their level of engagement by it (here, PMDT management guideline). This often could be inferred from both the recipient of the intervention and the personnel responsible for delivering the intervention. Table 1 summarizes these analytical constructs. The following characteristics of the healthcare providers were also included in the model: age, sex, work experience and professional care.

**Table 1:** Analytical constructs and their operational definitions in this study

Analytical construct	Operational definition in this study
**Implementation fidelity**	Adherence to the Programmatic Management of Drug-resistant Tuberculosis guidelines
**Facilitation strategies**	Training on and provision of protocols and guidelines to health care providers
**Quality of delivery**	Supportive supervision, coaching and mentorship for implementation of the guidelines
**Intervention complexity**	Perceived difficulty of implementing the guidelines by health care providers
**Participants’ responsiveness**	The extent to which the health care providers understand the content of the guidelines

### Study setting, design and population

The study was conducted at the Infectious Disease Hospital (IDH), Kano, Nigeria. The IDH is a public specialized secondary health care facility serving a population of about 1.5 million people and having a patronage of about 300 patients/day. The hospital is the only infectious diseases hospital in the whole of Northern Nigeria and serves as a referral centre for Tuberculosis (TB) and handles the treatment of a patient with Drug-resistant Tuberculosis. Hearing loss assessment for DR-TB patients is conducted in IDH as part of PMDT guidelines’ implementation. The implementation fidelity assessment was a cross-sectional study. All front-line health care providers that have been involved in the implementation of PMDT guidelines for at least 6 months before the enquiry in the facility were recruited. Staff that met the inclusion criteria but were unavailable for whatever reason during the period of the study were excluded.

Using 0.05 as the critical significant level with the power of 80%, the required minimum sample size of respondents was 73, (See Additional file 2 for Stata output on the sample size calculation). We reached out to all health care workers at the centre (*n* = 80). A total of 73 health care providers were successfully interviewed (response rate = 91%).

This study was guided by the STrengthening the Reporting of OBservational studies in Epidemiology (STROBE) checklist (Additional file 3).

### Study questionnaire

Data were collected through an interviewer-administered questionnaire developed primarily for this study (Additional file 4). This was developed based on the domains and constructs of the adapted implementation fidelity framework. A 5-point Likert scale of “5-Strongly agree, 4-agree, 3-neutral, 2-disagree and 1-strongly disagree” was used except for the facilitation strategy where Yes/No responses were used. The questionnaire was tested for reliability, as well as face and content validity in a pilot study. Implementation fidelity was measured by ten questions across its four constructs; facilitation strategy by six questions; quality of delivery by six questions; intervention complexity domain by three questions; and participants’ responsiveness by four questions. Other information collected is age, sex, professional cadre and duration of working in PMDT (work experience).

### Data management and analysis

The Cronbach-alpha reliability test was used to confirm the internal consistency of the questions used in measuring implementation fidelity and for each of the determinants. The alpha values ranged from 0.50 to 0.86, indicating moderate to high internal consistency. This allows summation of the constructs to derive a composite score for each of the variables. The ten constructs of implementation fidelity had a minimum score of 10 and a maximum score of 50. Likewise, scores were computed for each of the four core determinants. The values were then converted to percentages for analysis. The relationship between implementation fidelity and the determinants was examined using a linear regression model in Stata 14.2 (Inc. USA).

## Results

Table 2 presents the basic characteristics of the respondents. Table 3 shows that the implementation fidelity score ranged from 40 to 64% with a mean of 47.6% and a median of 46%. The range, mean with standard deviation and median with interquartile range for the four core determinates are also presented in Table 3. The unadjusted model in Table 4 shows that only quality of delivery, participants’ responsiveness, sex and professional cadre were significantly associated with implementation fidelity.

**Table 2:** Basic characteristics of the study population

Variable	N (%)
Age category	
≤ 35	28 (38.4)
> 35	45 (61.6)
Sex	
Male	14 (19.2)
Female	59 (80.8)
Professional cadre	
Doctors	13 (17.8)
Nurses	49 (67.1)
Principal Clinical Assistant	11 (15.1)
Work experience	
≤ 12 months	10 (13.7)
13–36 months	17 (23.3)
> 36 months	46 (63.0)

**Table 3:** Implementation fidelity scores

Variables	Range %	Mean (SD)	Median (IQR)
Implementation fidelity	40–64	47.6 (5.7)	46 (42–52)
Facilitation strategy	17–100	78 (27.1)	83 (83–100)
Quality of delivery	06–20	15 (04.3)	17 (13–19)
Intervention complexity	20–60	41 (13.8)	47 (27–53)
Participants’ responsiveness	25–45	36 (06.8)	40 (30–40)

*SD* Standard deviation, *IQR* Inter quartile range

**Table 4:** Regression outputs of implementation fidelity and its determinants

Factors	Unadjusted Model		Adjusted *(R*^*2*^ = 76.64)	
	Coefficient	95% Cl	Coefficient	95% CI
Facilitation strategies	0.18	−0.31-0.68	−0.64^a^	−1.23--0.05
Quality of delivery	1.91^a^	1.11–2.71	1.15^a^	0 .17–2.12
Intervention complexity	−0.69	−1.65 - 0.27	1.24^a^	0.53–1.95
Participants’ responsiveness	3.82^a^	2.07–5.56	0.57	−1.88 -3.01
Age	0 .32	−1.76 -2.41	−1.13	− 1.13
Work experience	0.45	−0.29 - 1.21	−0.14	− 0.14
Sex				
Female (Ref)	0.00		0.00	
Male	10.37^a^	8.03–12.71	0.67	0.67
Professional Cadre				
Doctors (Ref)	0.00		0.00	
Nurses	−11.31^a^	−13.67- -8.95	−12.80^a^	− 12.80^a^
Principal Clinical Assistant (PCA)	−9.34^a^	−12.45- -6.24	− 13.25^a^	− 13.25^a^

^a^Statistical significance at 5% level

R^2^ = the value accounting for the internal variation of implementation fidelity in the model

In the adjusted model, facilitation strategies became significant and exerting a negative effect on implementation fidelity. Implementation fidelity decreases by 0.64 for every unit increase in facilitation strategy. On the other hand, for each unit increase in quality of delivery, implementation fidelity increased by 1.15. Similarly, implementation fidelity increased by 1.24 with each unit increase in intervention complexity. Implementation fidelity score had a negative relationship with healthcare providers that were not medical doctors.

The eight predictors in the model explained about 77% of the variability in the model (*R*^*2*^ = 76.64). Thus, the characteristics measured in the model explained a good proportion of implementation fidelity in this context of management guidelines for hearing loss and its determinants.

## Discussion

Using primary data, this study measured implementation fidelity of the management guideline for hearing loss resulting from the treatment of drug-resistant Tuberculosis among healthcare providers in the Infectious Disease Hospital, Kano, Nigeria. The study found the implementation fidelity to the PMDT guideline to be low among the 73 health care providers surveyed. The implementation fidelity was significantly influenced by facilitation strategies, quality of delivery, intervention complexity, and the professional cadre of the health care providers. Low implementation fidelity of the PMDT guideline among healthcare providers may account for the rise in the prevalence of hearing loss resulting from DR-TB treatment in Nigeria. The finding resonates with the concept that the degree to which the interventions are implemented affects the outcome of the intervention [17]. Programs and interventions implemented with low fidelity usually lead to poor outcomes [20, 21].

This study found that facilitation strategies (largely training of staff) hurt implementation fidelity. This finding is contrary to the expected positive influence that it should exert [12, 22]. Thus, our study suggests that more training does not necessarily improve the implementation fidelity of interventions. From the literature, this observed negative effect may be attributable to training fatigue or the complexity of the training content or its delivery. Studies have established that programs are executed well with fidelity when they are simple, clear and detailed than when they are vague and non-specific [17, 19, 23]. But further studies may be necessary to provide context-specific knowledge on the gaps concerning the PMDT implementation in Nigeria.

The availability of supportive supervision (quality of delivery) and clear roles in the management guidelines (intervention complexity) positively affected implementation fidelity in this study. Similarly, higher implementation fidelity of intervention is achieved when a program is acceptable to the participants responsible for the implementation and recipients of the intervention [23–25]. There is evidence from the literature that coaching, mentorship and supportive supervision is very effective in improving fidelity, acceptability and sustainability of the intervention [26, 27].

Nurses and principal clinical assistants were found to have significantly lower fidelity scores than the doctors. Whereas ther reasons for this are unclear from the results of this cross-sectional study, possible difference in training as well as the team leadership role of doctors in the center may have contributed to this outcome. The level of training and leadership have been found in the literature to influence implementation fidelity [28].

A counterintuitive finding in this study was that there was association between work experience and implementation fidelity. There is evidence from the literature that implementation fidelity improves with length of experience, given that length of experience is usually associated with self-confidence [29]. However, we spaculate that the highly specialized nature of the work done in this centre, as well as the recency of the guidelines may have a role to play in the findings of our study. Future research is needed to explore these findings further.

### Strengths and limitations of the study

Our questionnaire was developed by the authors specifically for this contextaual study. This exposes the questionnaire to potential bias. To mitigate potential bias, both face and content validity tests were conducted. The content validity in this study was done using the adapted constructs developed by Carol et al. Further, face validity was tested by five different practitioners with experience on the research subject. These experts screened the questionnaire and confirmed that the constructs and domains of interest were adequately captured. Each item in the questionnaire was examined thoroughly with the intent to evaluate whether each of the measuring items corresponds with the given conceptual domain of the framework.

The use of only quantitative survey method in this health services research limits its ability to understand the depth of the phenomenon being investigated. For example, whereas participants’ responsiveness is one of the core determinants that can affect the implementation of fidelity [12], this study did not find any association between participants’ responsiveness and implementation fidelity. A follow-up qualitative research would be able to explain this phenomenon. That being said, this study has provided useful insights and measurable situation analysis which future studies can explore more comprehensively using qualitative methods.

## Conclusion

The study found implementation fidelity of management guideline for hearing loss resulting from Drug-resistant Tuberculosis treatment to be low among health care providers at the infectious disease hospital, Kano, Nigeria. Training and retraining, though important, maybe counterproductive if used predominantly. To improve fidelity in this context, efforts should focus on supportive supervision, coaching and mentorships. Such strategies may go a long way to reduce the incidence of hearing loss, thus improving patients compliance with the treatment regimen, and ultimately reducing the burden of DR-TB in Nigeria.

## Supplementary Information

**Additional file 1.** PMDT management guidelines. PMDT management guidelines.

**Additional file 2.** Stata output on the sample size calculation. Stata output on the sample size calculation.

**Additional file 3.** STrengthening the Reporting of OBservational studies in Epidemiology (STROBE) checklist. STROBE checklist for observational studies.

**Additional file 4.** Study questionnaire. Study questionnaire.

## Data Availability

The datasets used and/or analysed during the current study are available from the corresponding author on reasonable request.

## Abbreviations

DR-TB: Drug Resistant Tuberculosis
IDH: Infectious Disease Hospital
PMDT: Programmatic Management of Drug-resistant Tuberculosis
TB: Tuberculosis
WHO: World Health Organiation
