# Supplementary material for: Clinical guidelines for managing hearing loss as a complication of drug-resistant tuberculosis treatment: an evaluation of implementation fidelity in Kano, Nigeria
Source: BMC Health Serv Res. 2022 Feb 3;22:142. doi: 10.1186/s12913-022-07536-y (PMC8812187; doi:10.1186/s12913-022-07536-y)
Supplement: Supplementary file 1 — Additional file 1. PMDT management guidelines. PMDT management guidelines. [file 12913_2022_7536_MOESM1_ESM.pdf]

GUIDELINES ON THE USE OF THE SHORTER REGIMEN AND NEW DRUGS IN THE CLINICAL AND PROGRAMMATIC MANAGEMENT OF DRUG RESISTANT TUBERCULOSIS AND CO-INFECTIONS OF NIGERIA.

(AN ADDENDUM TO NTBLCP 2016 PMDT GUIDELINES – 2ND EDITION)

OCTOBER 2017

List of Contributors

The following institutions and organizations participated in the workshop to adapt this document for Nigeria:

Federal Ministry of Health (FMOH),National Tuberculosis and Leprosy Control Program (NTBLCP) and its Partners: World Health Organization (WHO), United States Agency of International Development (USAID),Institute of Human Virology Nigeria (IHVN), The German Leprosy Relief Association (GLRA), The Leprosy Mission Nigeria (TLMN), Clinton Health Alliance Initiative (CHAI), KNCV Tuberculosis Foundation (Nigeria), Management Systems for Health (MSH), University of Port Harcourt Teaching Hospital (UPTH), FHI360, Systems for Improved Access to Pharmaceuticals and Services (SIAPS)

Their support was highly appreciated and demonstrates their commitment to improving tuberculosis control in Nigeria

List of Abbreviations

ACSM	*Advocacy, Communication and Social Mobilization*
ADR	*Adverse Drug Reaction*
aDSM	*Active Drug Safety Monitoring and management*
AE	*Adverse Event*
ALAT	*Alanine aminotransferase*
Am	*Amikacin*
Amx/Clv	*Amoxicillin/Clavulanate*
ASAT	*Aspartate aminotransferase*
ART	*Anti-Retroviral Therapy*
Bdq	*Bedaquiline*
BMI	*Body Mass Index*
CSF	*Cerebrospinal Fluid*
Cfz	*Clofazimine*
Cm	*Capreomycin*
CrCl	*Creatinine Clearance*
Cs	*Cycloserine*
CTB	*Challenge TB*
Dlm	*Delamanid*
DOT	*Directly Observed Treatment*
DRS	*Drug Resistance Surveillance*
DR-TB	*Drug-Resistant Tuberculosis*
DS-TB	*Drug-Susceptible Tuberculosis*
DST	*Drug Susceptibility Testing*
E	*Ethambutol*
ECG	*Electrocardiogram*
EMA	*European Medicines Agency*
FDA	*Food and Drug Administration*
FLD	*First-line Drugs*
FMOH	*Federal Ministry of Health*
FQ	*Fluoroquinolone*
GDF	*Global Drug Facility*
gDST	*Genotypic Drug Susceptibility Testing*
H	*Isoniazid*
H^h	*Isoniazid High Dose*
HIV	*Human Immunodeficiency Virus*
Imp/Cln	*Imipenem/Cilastatin*
ITR	*Individualized DR-TB Treatment Regimen*
Km	*Kanamycin*
Lfx	*Levofloxacin*
LGAs	*Local Government Areas*
LPA	*Line Probe Assay*
LTFU	*Loss to Follow-Up*
Lzd	*Linezolid*

MDR-TB	*Multidrug-Resistant Tuberculosis*
Mpm	*Meropenem*
Mfx	*Moxifloxacin*
MGIT	*Mycobacteria Growth Indicator Tube*
M&E	*Monitoring and Evaluation*
ND&R	*New Drugs and Shorter Regimens*
NDR-TBC	*National DR-TB Committee*
MTB	*Mycobacterium Tuberculosis*
NAFDAC	*National Agency for Food and Drugs Administration and Control*
NRL	*National TB Reference Laboratory*
NTBLCP	*National Tuberculosis and Leprosy Control Program*
NTM	*Non-Tuberculosis Mycobacterium*
Ofx	*Ofloxacin*
PAS	*Para Aminosalicylic Acid*
pDST	*Phenotypic Drug Susceptibility Testing*
PK	*Pharmacokinetics*
PMDA	*Pharmaceuticals and Medical Devices Agency of Japan*
PMDT	*Programmatic Management of Drug Resistant Tuberculosis*
PLHIV	*People Living with HIV*
PSM	*Procurement and Supply Management*
Pto	*Prothionamide*
R	*Rifampicin*
RR	*Rifampicin Resistance*
RR-TB	*Rifampicin Resistant Tuberculosis*
SAE	*Serious Adverse Event*
SLD	*Second-Line Drugs*
SLI	*Second-Line Injectable*
SL-LPA	*Second-Line Line Probe Assay*
SNRL	*Supra National Reference Laboratory*
SOP	*Standard Operating Procedures*
STBLCO	*State TB & Leprosy Control Officer*
STR	*Shorter DR-TB Treatment Regimen*
TB	*Tuberculosis*
TBIC	*Tuberculosis Infection Control*
TSH	*Thyroid Stimulating Hormone*
ULN	*Upper Limit of Normal*
WHO	*World Health Organization*
XDR-TB	*Extensively Drug-Resistant Tuberculosis*
Z	*Pyrazinamide*
ZRL	*Zonal Reference Laboratory*

Table of contents

Contents

List of Contributors 2
List of Abbreviations 3
Table of contents 5
1. Introduction 8
2. Detection of drug-resistant tuberculosis 9
2.1 DR-TB case detection 9
2.2 Case Finding Activities 9
3. Diagnosis of drug-resistant tuberculosis 10
3.1 Clinical Presentation 10
3.2 Bacteriological diagnosis 10
3.3 Approach to discordant Results: 12
3.4 Diagnosis of extra-pulmonary tuberculosis (EP-TB): 13
3.5 Diagnosis of drug-resistant tuberculosis in children 16
4. Treatment of drug-resistant tuberculosis 22
4.1 Eligibility criteria for starting DR-TB treatment regimens 22
4.2 Patient triage approach to select the appropriate DR-TB regimen 22
4.3 DR-TB Treatment Regimens 24
4.4 Treating mono and poly-drug resistant TB 344
4.5 Adjuvant therapy 344
4.6 Role of surgery 35
5. Treatment of DR-TB in special conditions and situations 36
5.1 Pregnancy 36
5.2 Breast feeding: 37
5.3 Children 37
5.4 Liver disease 38
5.5 Renal disease 38
5.6 Diabetes Mellitus 39
5.7 Seizure disorders 39
5.8 Psychiatric disorders 39
5.9 Psycho-active substance dependence 40
6.HIV and drug-resistant tuberculosis. 41

6.1 General considerations in the management of DR-TB and HIV co-infection 41
6.2 Clinical features and diagnosis of DR-TB in HIV infected patients 41
6.3 Drug-resistant TB and HIV co-treatment 42
6.4 Potential overlapping toxicities in the treatment of HIV and drug-resistant TB 42
6.5 Drug-drug interactions in the treatment of HIV and drug-resistant TB 44
6.6 Use Bedaquiline and Delamanid with ART 44
7. Hepatitis C (HCV) 46
7.1 Epidemiology 46
7.2 Natural history 46
7.3 Screening 47
7.4 Genotype testing 47
7.5 Assessment of treatment 47
7.6 Monitoring of treatment 50
7.7 Special considerations for specific population 51
8.Treatment monitoring 54
8.1Monitoring progress of treatment 54
8.2Monitoring Adverse Events 55
9. Management of Adverse Events 63
8.1Severity grading scale of adverse events and main laboratory parameters 63
8.2 Clinical Management of adverse events 64
Peripheral neuropathy 73
10. Management of close contacts of DR-TB patients 85
10.1 Contact Investigation 85
10.2 Prophylaxis in children and adults exposed to DR-TB 85
11. Active TB Drug Safety Monitoring and Management (aDSM) 86
11.1 aDSM definition and objective 86
11.2 Key steps to implement aDSM 86
11.3 Strategy for aDSM implementation 87
10.4 Common definitions used in aDSM 87
11.5 Levels of monitoring in aDSM 89
11.6 Causality assessment 89
11.7 Recording and reporting of adverse events (AEs) 90
12. Procurement supply and management of shorter regimen commodities 91
13.Supervision, monitoring and evaluation for DR-TB 92
13.1 Information Flow System 92
13.2 Supervision 92
13.3 Program Monitoring 92

13.4 DR-TB Program Indicators 92
13.5 Routine Data Quality Assurance (RDQA) 95
13.6 Recording and Reporting Tool 96
13.7 Evaluation 96
13.8 Treatment Outcome Definitions 97
References 98
Annexes: 100
Annex A: Reference Laboratory services and testing capacity 100
Annex B: Adjustment of Anti-TB medicine dosages in patients with renal insufficiency with clearance < 30 ml/min 101
Annex C: New and repurposed drugs: Indications, adverse drug reactions, monitoring, contraindications, remarks/precautions and drug interactions. 102

1. Introduction

This document describes the steps necessary to implement shorter regimen and new drugs for DR-TB treatment including diagnosis and bacterial confirmation of drug resistance, treatment regimen design, monitoring of treatment efficacy and safety, and programmatic evaluation.
Recent international experience demonstrates that for DR-TB patients without additional resistance or intolerance to key second-line drugs (SLD), i.e. fluoroquinolones (FQ) and second line injectable (SLI), the treatment duration can be substantially shorter, thus reducing the burden for the patients and National TB Programs.In May 2016 WHO issued a recommendation on the use of a shorter DR-TB regimen based on eligibility criteria [2]. One of the main requirements for successful introduction of shorter DR-TB treatment regimens is an ability to rule out resistance to key drugs(fluoroquinolone and second-line injectable), given the dependence of the shorter regimen on these drugs. For patients not eligible for shorter regimen, individualized regimensare recommended which will include the new drugs-Bedaquiline (Bdq) and Delamanid (Dlm).

WHO has published interim policy guidance on the use of Dlm and Bdq inDR-TB patients [7][8].
Laboratory support is key to implementing the new treatment regimens.Rapid molecular tests are now available for early detection of rifampicin resistant TB (RR-TB) and resistance to key second line drugs (SLDs) in Nigeria.There has been rapid scale-up of the Xpert MTB/RIF test, however access to line probe assay (LPA) is still limited. In light of this, there is an ongoing effort to improve access.

The conventional RR/MDR-TB treatment currently in use in Nigeria involve standardized second-line anti-TB drugs for a minimum period of 20 months. For patients with SLD resistance (pre-XDR/XDR-TB) the treatment is partially individualized according to contact history, drug history and DST result (if available).

The country has adopted the new shorter and individualized regimensfor the clinical and programmatic management of DR-TB. The implementation of this addendum will be in phases.

2. Detection of drug-resistant tuberculosis

2.1 DR-TB case detection

The diagnosis and treatment of persons with DR-TB starts with the proper identification of a presumptive DR-TB case. Therefore, it is important that all those who provide health services to TB patients are able to identify persons with presumptive DR-TB at all times and to ensure that this identification is done early, to enable prompt diagnosis and appropriate treatment.

To increase surveillance for DR-TB in Nigeria, the NTBLCP has recommended that all health care providers give priority to the following persons who present with symptoms of TB in their facilities:

i. Anybody who has come in contact with a confirmed DR-TB patient and shows symptoms of TB.
ii. Any person whose AFB result is smear positive when repeated at the end of month threeof Regimen 1 treatment.
iii. All previously treated Drug Susceptible TB Patients:
    a. Relapse
    b. Treatment after failure to Regimen 1
    c. Treatment after loss to follow-up
    d. Other previously treated patients.

Similarly, in order to expedite the early diagnosis and improve the quality of services, the NTBLCP has also identified a second priority group of patients which include:

- All persons with a smear negative AFB result who still show symptoms of TB after one week of administration of broad spectrum antibiotics
- All persons living with HIV (PLHIV) who present with symptoms of TB
- All health care staff who present with symptoms of TB
- All children who present with symptoms of TB
- All persons with symptoms suggestive of extra-pulmonary TB (see table below) in which a specimen could be collected for Xpert MTB/RIF test such as collection of cerebrospinal fluid (CSF) for examination as in TB meningitis. [12]

2.2Case Finding Activities

In the National Plan for the introduction of ND&R the following activities related to case finding were included:

- **Assess and address factors related to low utilization of Xpert MTB/RIF test:**Conduct campaigns and sensitization activities to improve test requests by clinicians; expand laboratory capacity (Xpert MTB/RIF, second line LPA, culture and pDST)
- **Active case finding activities at Health unit level:** Screening of TB in HIV patients (consultation and counseling rooms); screening for TB in OPDs, antenatal care, pediatric consultations (specific focus in malnourished children); screening of TB in waiting areas and reinforce request of genotypic and phenotypic DST for all TB retreatment patients.
- **Sensitize the staff at DS-TB& DR-TB wards, OPDs and DOT centers** to systematically requestXpert MTB/RIF andchest X-ray (when required)to all symptomatic TB Contacts (special focus on children). Perform contact investigation throughhome visit by volunteers.
- **Engaging local CBOs** to provide targeted community education and outreach for active case finding (focus in high dense populated areas as urban slums).

3. Diagnosis of drug-resistant tuberculosis

3.1 Clinical Presentation

Clinical features of DR-TB are not particularly different from those of drug susceptible TB (PTB and EPTB). Clinicians should be alert to the complications of TB, which may be more common in patients with DR-TB.

Drug-resistant TB is by definition a bacteriological diagnosis.However, in patients where bacteriological confirmation is unlikely, such as symptomatic children, PLHIV, or those with extra-pulmonary TB who are also close contacts of known DR-TB patients, a provision for the clinical diagnosis of DR-TB can be put in place.

3.2 Bacteriological diagnosis

The diagnosis of DR-TB is done by genotypic and phenotypic drug-susceptibility tests (gDST/pDST)): Xpert MTB/RIF test, Line Probe Assay (first and second line LPA) and culture/pDST.

The NTBLCP endorsed the WHO 2013 policy update that recommends Xpert MTB/RIF as the initial diagnostic test in all adults and children with signs and symptoms of TB (see Algorithm 1).

The NTBLCP also recently endorsed the WHO 2016 policy that recommends the use of GenoType MTBDR*sl* Version 2 Line Probe Assay (SL-LPA) for patients with confirmed Rifampicin Resistant (RR)TB regardless of the sputum smear result as initial test to detect resistance to fluoroquinolones (FQs) and second-line injectable drugs (SLIDs).

For all patients with RR-TB, samples should be sent for FL &SL-LPA, culture and phenotypic DST.

Regarding culture and phenotypic DST, the time to inform the results as follow:

- To inform a positive result:
  - MGIT: from 2-3 weeks.
  - LJ:from 4-8 weeks.
- To inform a negative result:
  - MGIT: 6-7 weeks.
  - LJ: 8-9 weeks.
- To inform pDST: (from the day that culture was positive)
  - MGIT: 2-3 weeks.
  - LJ: 4-5 weeks.

Phenotypic DST(pDST) results:

- Are reliable and reproducible for R and H, and Km, Am, Cm, Ofx, Lfx.
- Mfx & Gfx: there is a need for their critical concentrations to be re-evaluated.
- E,Z, Eto/Pto, Cs, PAS: are neither reliable nor reproducible.
- New drugs Bdq, Dlm: MIC (minimum inhibitory concentration) is defined, recommendations on implementation and updated guidelines are expected.

- Repurposed drugs Cfz, Lnz: need validation.

There are eight TB reference laboratories with the capacity to perform baseline culture/phenotypic DST (pDST) and the first line LPA. However, currently four laboratories have additional testing capacity for the second line culture/pDST and SL-LPA. (Annex A, Table 16: NTBLCPZoning of TB Reference Laboratories by State and Testing Capacity).

Figure 1: Diagnostic Algorithm Xpert MTB/RIF[17]

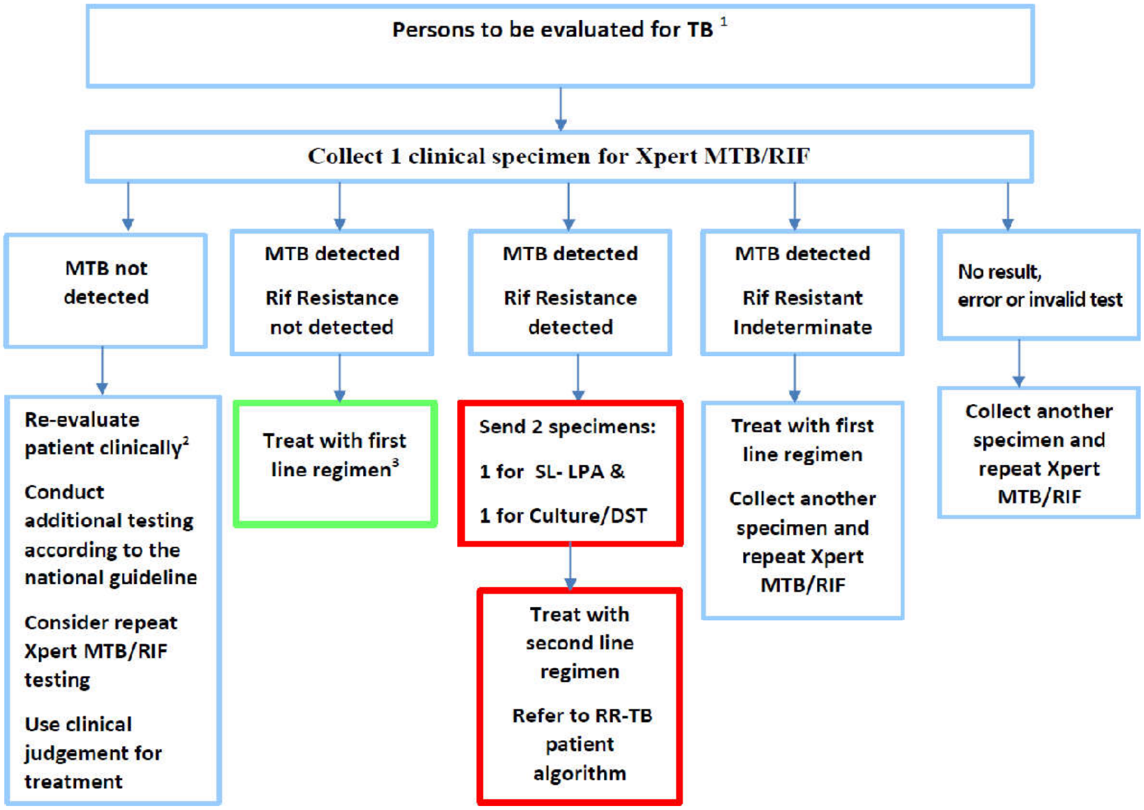

[1]Persons being evaluated for TB include adults and children with signs or symptoms suggestive of TB or with a chest X-ray with abnormalities suggestive with TB. This algorithm may also be used for persons being evaluated for extra-pulmonary TB (with other specimens as lymph node aspirate, CSF etc). Priority patients for Xpert MTB/RIF are PLHIV, children and risk of DR-TB (previously treated, non-converters: smear positive at end of intensive phase, DR-TB contacts)

[2]Further investigations for TB may include Chest-X Ray and additional clinical assessment. For persons being evaluated for TB who are HIV positive and have CD4 counts ≤100 cells/µl or are seriously ill it is possible to perform urine lateral flow lipoarabinomannan (LF-LAM) assay (when available).

<u>If there is strong clinical evidence of TB start TB treatment (clinically diagnosed TB).</u>

In HIV+ patients evaluate differential/concomitant diagnosis: Non-tuberculosis mycobacterium (NTM), pneumocystis Jirovecii pneumonia (PCP), pulmonary Kaposi's sarcoma,lymphoma, fungal pneumonia, herpes simplex or cytomegalovirus (CMV) pneumonitis, children HIV+,lymphoid interstitial pneumonitis (LIP).

[3] If patient is high risk of DR-TB (retreatment cases more than two times, non-converters: smear positive at end of intensive phase, DR-TB contacts) send additional samples for FL and SL-LPA and culture/pDST.

3.3 Approach to discordant Results:

Discordant results are not common, usually when comparing culture-based results with molecular results. Each discordant result will need to be investigated, on a case-by-case basis. DNA Sequencing can solve the dilemma (when available in future). [17]

General considerations are:

1. **Xpert MTB/RIF MTB detected, culture negative.**

<u>The Xpert MTB/RIF result should be used to guide treatment decision pending additional testing.</u>
Cultures from persons with pulmonary TB may be negative for a variety of reasons including:

- Patient being treated for TB.
- Transport or processing problems that inactivated the tubercle bacilli.
- Cultures lost to contamination.
- Inadequate testing volume.
- The discrepancy may be due to laboratory or clerical error.

Follow-up actions may include:

- Re-evaluate the patient for TB.
- Reassess possibility of prior or current treatment with anti-TB drugs (including fluoroquinolone use).
- Evaluate the possibility of laboratory or clerical error, and repeat culture.

2. **Xpert MTB/RIF MTB not detected, culture positive.**

<u>Treatment decision should be based on the culture result:</u>

- The culture-positive result should be considered as bacteriological confirmation of TB (culture is more sensitive).
- Using a sputum specimen, Xpert MTB/RIF has a pooled sensitivity of 89% for detecting MTB compared to culture.
- Xpert MTB/RIF sensitivity is lower in PLHIV, children, and other specimen types such as CSF.
- False positive cultures are very rare (due to laboratory errors such as cross-contamination and sample labeling problems).

Follow-up actions may include:

- Re-evaluation of the patient for TB and response to anti-TB therapy.
- Conduct additional testing using Xpert MTB/RIF.
- Process and culture additional samples.
- Evaluate the possibility of laboratory or clerical error.

3. **Xpert MTB/RIF MTB detected, rifampicin resistance detected; rifampicin susceptibleby phenotypic DST.**

<u>The Xpert MTB/RIF result should be used to guide treatment decisions pendingadditional testing</u>.

- Certain mutations are known to generate this discordant result, particularly in the BACTECTM MGITTM system (i.e., a false-susceptible phenotypic result).
- In some low DR-TB prevalence settings, silent mutations have been observed that generate a false resistant MTB/RIF result, but these tend to be very rare.

Follow-up actions may include:

- DNA sequencing (where available).
- Phenotypic DST using solid media.

- Evaluating the possibility of laboratory or clerical error.

4. **Xpert MTB/RIF MTB detected, rifampicin resistance not detected; rifampicin resistant by phenotypic DST.**

Treatment decisions should be based on the phenotypic DST result.

False rifampicin-susceptible Xpert MTB/RIF results are rare but have been observed:

- 1–5% of TB cases tested in various epidemiologic settings. Mutations in the region of the *rpoB* gene sampled by the Xpert MTB/RIF tests have been shown to account for 95-99% of rifampicin resistance. The remainder of rifampicin resistance arises from mutations outside the sampled region, which produce anXpert MTB/RIF result of rifampicin resistance not detected.

Follow-up actions may include:

- DNA sequencing (where available).
- Repeating the phenotypic DST.
- Evaluating the possibility of laboratory or clerical error.

5. **Xpert MTB/RIF MTB detected rifampicin resistancenot detected; FL LPA rifampicin resistancedetected.**

Treatment decision should be based on FL LPA (Rif resistant).

This discordance is rare, due to Hetero-resistant strains. Different populations of bacteria are co-existing with varying susceptibility to TB drugs (some are resistant and some sensitive). Depending on the treatment and “fitness” of the bacteria, different DST results from different samples may occur (especially if an interval has elapsed). Rif hetero-resistance can be detected in FL-LPA (detect absence of wild type & mutations) but not always in Xpert MTB/RIF (detects resistance by absence of wild type and single copy target of rpoB). The culture and pDST results can vary too (Rif sensitive or resistant), according to the prevalent population.[17]

3.4 Diagnosis of extra-pulmonary tuberculosis (EP-TB):

- Diagnosis of DR-TB in extra-pulmonary TB patients can be challenging due to difficulties in sample collection as also bacteriological confirmation (low yield in EPTB samples).
- Usually diagnosed by clinical symptoms/signs and/or investigations (clinically diagnosed TB).
- Constitutional symptoms may be absent (fever, weight loss).
- Young children and HIV positive patients are more susceptible to EPTB.
- EPTB may be associated with PTB (search for symptoms and refer sputum samples for investigation).

Table 1: Diagnosis of extra-pulmonary tuberculosis

**Extra-pulmonary tuberculosis (EPTB**			
**Site**	**Symptoms and signs**	**Investigations**	**Comments**
**Lymph node TB**<br>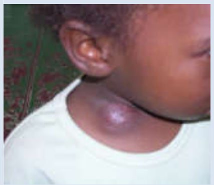	One or more enlarged lymph nodes(can soften andform a sinus).<br><br>More common in the neck, but also axillae, inguinal, or inside the chest or abdomen.	**Needle aspiration** If node is fluctuant (easy). Send sample for Xpert MTB/RIFtest and culture.<br><br>**Fine needle aspiration cytology (FNAC)** if not fluctuant (difficult). Sample referred for cytology.	**TB related lymphadenopathy can also occur inside the chest or abdominal cavities:**<br>**Chest X-ray:**nodes in chest (mediastinum enlargement).<br><br>**Abdominal ultrasound:** intra-abdominal lymph nodes.
**Pleural Effusion (Pleural TB)**<br>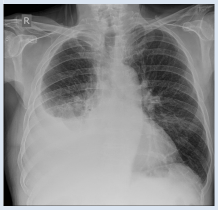	Initially asymptomatic, later chest pain (usually unilateral)<br>Shortness of breath.<br>Dullness on percussion and reduced breath sounds.<br><br>Common in young adults.	**Chest X-ray**: Obliteration of costophrenic angle, if large - massive homogeneous opacity with fluid level.<br><br>In high TB burden setting diagnosis is clinical (unilateral pleural effusion).<br><br>Pleural tap only necessary in case of severe dyspnea to alleviate symptoms.	If pleural tap is done, refer sample for AFB, Xpert MTB/RIF testand culture (low positivity rate for AFB <5%, culture <15%).<br><br>Differential diagnostic of bilateral pleural effusion is wider.<br><br>If pleural tap yields pus, consider empyema and refer to hospital for drainage.<br><br>If hemorrhagic, refer to higher level to exclude malignancy.
**Spine**<br>**(Pott disease)**<br>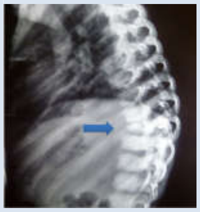	Affects vertebrae and disks.<br>Localized pain in the spine followed by deformation &destruction (dorsal or lumbar).<br>If neurologicalcompromise: paresthesia, paralysis, sensory loss and/or incontinence may be present.	**X-ray of the spine:**<br>Destruction of vertebrae and/or intervertebral disk.	Destruction of the spine can lead to neurological compromise.<br><br>Refer to physiotherapy and consult with spinal or orthopedic surgeon.
**Joint**<br>**(TB Arthritis)**<br>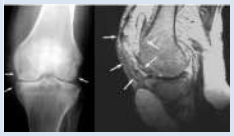	Chronic monoarthrits, limitation of movement.<br>Swelling usually involving hip, knee or elbow.	**X-ray of the affected joint:** joint destruction.<br>**Needle aspiration, synovial Biopsy:** specimens can be referred for Xpert MTB/RIF test and culture/pDST.	Refer to specialist.

**Abdominal (Abdominal TB)**<br>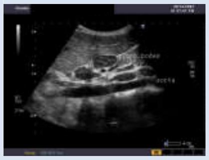	Non-specific symptoms (abdominal pain, abdominal distention due to ascitic fluid, chronic diarrhea, abdominal mass etc.)	**Abdominal ultrasound:** can be seen intra-abdominal lymph-nodes, ascites or abdominal mass.<br><br>If chronic diarrhea -**stool samples** can be sent for Xpert MTB/RIF test and/or culture/pDST<br><br>If **ascitic tap**: refer sample for AFB, Xpert MTB/RIF test, culture/pDST (low positivity rate).	Other investigations:<br>If ascitic tap, measure albumin in ascitic fluid and serum.<br><br>SAAG (Serum-ascites albumin gradient) less than 1.1 g/dl is consistent with TB.<br><br>Consider asitic drainage if necessary.
**Pericardium (Pericardial TB)**<br>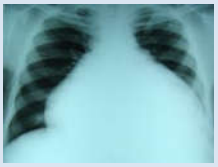	Chest pain, distant (faint) heart sounds, symptoms ofheart failure: dyspnea, peripheral edema, sometimes ascites etc.	**Chest X-ray:** enlargement of cardiac silhouette<br>**Echocardiogram** (to confirm that the enlarged heart shadow is due to effusion)	Severe forms (hemodynamic compromise) may need pericardiocentesis at hospital.<br><br>Add corticosteroids, if needed.
**Milliary TB**<br>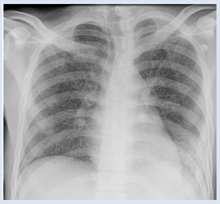	Constitutional symptoms (fever, weight loss), progressive deterioration of physical condition.<br>Nonspecific findings (wasted, respiratory distress, may have compromised conscious level).	**Chest-X-ray**:miliary pattern ("millet seeds").<br><br>In children 60-70% risk of involvement of meninges: perform lumbar puncture.	Known as disseminated TB, caused by haematological spread of bacilli throughout the body.<br><br>HIV status and history of a TB contact in children are important for the diagnosis.
**Meninges (TB Meningitis)**<br>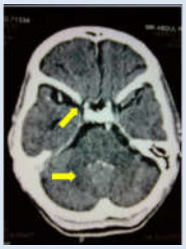	Headache, fever, confusion, vomiting,stiff neck,lethargy, loss of consciousness, photophobia, irritability, cranial nerve paralysis.<br>In infants hypotonia andbulging fontanelle.<br><br>More common in children <2 years and HIV positive patients.	**Lumbar puncture:**<br>Refer CSF sample for AFB, Xpert MTB/RIF test,culture/pDST (low positivity rate).<br><br>**CSF exams:**<br>Protein increased (>40 g/l), glucose diminished (< 60 mg/l), cell count (100-1000 white blood cells/ml, over 80% Lymphocytes).<br><br>**Brain CT scan/MRI**.	For differential diagnosis: Indian ink, Cryptococcus antigen in CSF and serum, VDRL, bacteriological culture.<br><br>Add corticosteroid, if needed.
**Genitourinary TB**	Renal involvement can be asymptomatic for a time	Clinical diagnosis	**Urine:** AFB is almost always negative.Xpert MTB/RIF test and

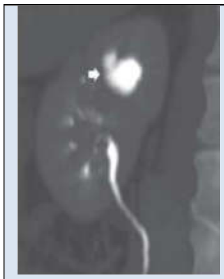	with slow development of dysuria, back flank pain.<br><br>Male: swelling of testes, epididymitis, hematuria etc.<br><br>Female: main complaint is **infertility** (nonspecific symptoms abdominal pain).	**Ultrasound** (renal, testes, gynecological).<br><br>**Cystoscopy:** urethral strictures.	culture/pDST can be done after centrifugation (low yield).
**Cutaneous TB**<br>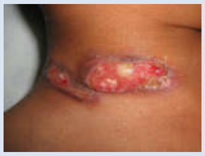	Chronic, painless, non-pathognomonic lesions (from small papules, erythema to large tuberculomas).	Clinical diagnose plus biopsy (pathology, culture).	

3.5 Diagnosis of drug-resistant tuberculosis in children

Much of the material for DR-TB in children is reused with permission from the Sentinel Project and the Union guidelines [9], [10], [14].

The diagnosis is mainly based on careful and thorough clinical assessment with support of complementary investigations when available (chest X-ray, other tests for EPTB - see Table 1)

3.5.1 Key features to consider for diagnosis of TB/DR-TB in children:

1) **Age related risk of TB disease progression and severity:**
   - There is a higher risk in children**younger than two**, with more risk for dissemination and severe disease.
   - For children above the age of 10 (adolescents), the presentation and diagnosis of pulmonary TB is similar to adults.

2) **HIV Status**: HIV testing should be offered to all children diagnosed with TB

3) **History of TB/DR-TB contact.**

Assessment of TB contact:

- Household / Close contact: when the index TB patient is living in the same household or has frequent contact with the child (e.g., neighbor, relative, caretaker)
- If no index case is identified, always enquire about anyone in the household or frequent visitor with TB symptoms or who died recently.
- In older children contact with a TB source case may be outside the household, e.g., school
- Timing of contact: children usually develop TB within two years after exposureand most (90%) within the first year.
- Assess if the contact has risk or confirmed DR-TB (If the contact died, failed the treatment, or is not adherent).

4) Nutritional status: weight loss or poor weight gain - faltering weight**(assess the growth chart)**

5) Signs and symptoms of TB: Pulmonary TB (PTB) and extra-pulmonary TB (EPTB)

Common signs and symptoms of PTB in children:

- Cough (especially if is persistent more than two weeks and not improving with broad spectrum antibiotics).
- Fever and/or night sweats.
- Weight loss or failure to thrive - follow growth chart.
- Fatigue, less active, reduced playfulness.

TWO OR MORE OF THESE SYMPTOMS ARE HIGHLY SUGGESTIVE OF TB DISEASE

For presumptive EPTB in children refer toTable 1: Diagnosis of extra-pulmonary TB

6) Risk of DR-TB in children

A high index of clinical presumption is needed for timely diagnosisof DR-TB in children.

Child with risk of DR-TB

- Close contact with a confirmed DR-TB patient.
- Close contact with a patient that died from TB, failed or is not adherent to TB treatment.
- History of previous TB treatment (in the past 6-12 months).
- Not improving after 2-3 months of first line TB treatment, including persistence of positive smear or culture, persistence of symptoms, and failure to gain weight (radiological improvement is frequently delayed).

Treatment should be started without waiting for bacteriological confirmation(based on the DST of the index patient if available or empirical treatment). All efforts should be made to refer samplesfrom the children for Xpert MTB/RIFtest and culture/pDST. Early detection and initiation with appropriate treatment is essential to ensure favorableoutcome.

7) Bacteriological confirmation:

Bacteriological confirmation is important however, this is not always feasible. Results are often negative due to paucity of bacilli in clinical specimen and difficulty in sample collection. **All efforts should be made to collect and send samples.**

Xpert MTB/RIF is the recommended first-line diagnostic test in children. It may be positive in less than one third of children with TB (a negative result does not exclude TB – particularly in children).**Culture/pDST**is more sensitive, but is only positive in <30-40% of symptomatic children.

Refer to SOPs for collection of samples procedures: sputum, induced sputum, gastric lavage, and other samples for EPTB as fine needle aspiration for TB Lymph node, lumbar puncture CSF, etc.

8) Chest X-ray

In children with pulmonary TB, the more common abnormality is **enlarged hilar lymph nodes**. Cavitation tends to occur in older children. Other chest X-ray abnormalities are often non-specific: they can be similar to other diseases as lower respiratory tract infections and pneumonia. In HIV infected children, the differential or concomitant diagnosis also includepneumocystis jirovecii pneumonia (PCP) and lymphoid interstitial pneumonitis (LIP).

In some patients, the chest X-ray can be **normal**. In extra-pulmonary TB theX-ray can be useful to diagnose pleural TB, miliary TB, pericardial TB, and spinal TB.

9) Tuberculin skin test (TST)

Not usually included in diagnostic algorithm as a result of availability and issues with interpretation.

A negative test does not exclude TB, a positive test doesn't mean active TB disease but can be useful to indicate TB infection when there is no known TB contact.

Positive results: ≥ 5 mm in HIV+ or malnourished children or ≥ 10 mm in all other children.

10) Hospitalization/referral criteria

- All children with DR-TB should be evaluated by state consilium to take the decision for the place of treatment – either community or treatment center.
- Children with diagnostic uncertainty requiring further investigations should also be referred to a higher-level facility.

3.5.2 Examples of chest X-ray in children

Figure 2: Examples of chest X-rayin children [14]

Pulmonary TB:

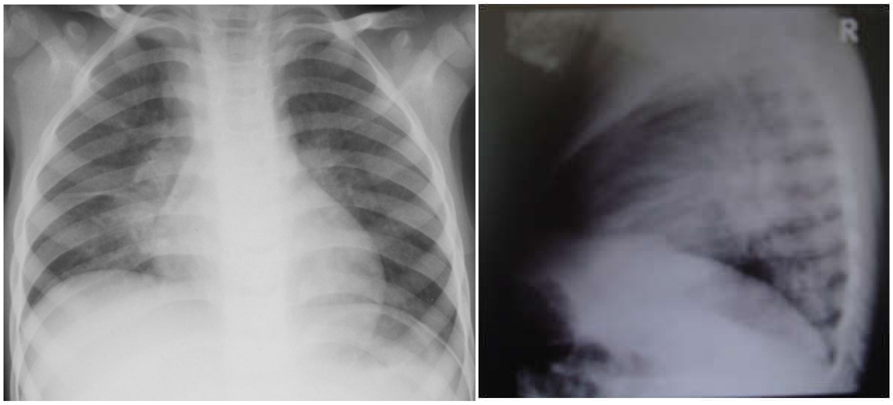

CXR suggestive of PTB: perihilar lymph node enlargement

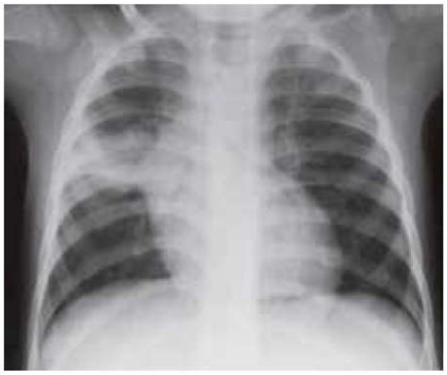

CXR suggestive of PTB: right perihilar lymph node enlargement with opacity in the right mid zone

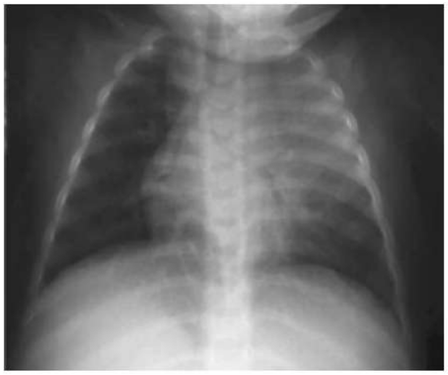

CXR suggestive of PTB: left upper lobe opacification with narrowing and shift of left main bronchus

Extra-pulmonary TB (EPTB):

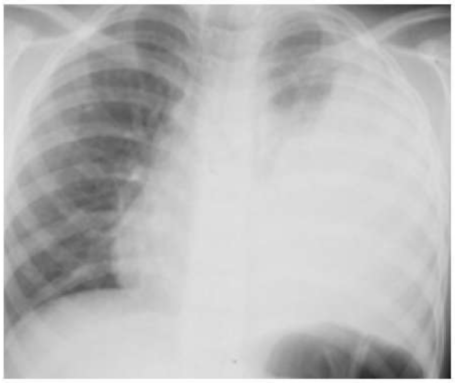

TB pleural effusion: large left-sided effusion. Pleural tap to differentiate from empyema

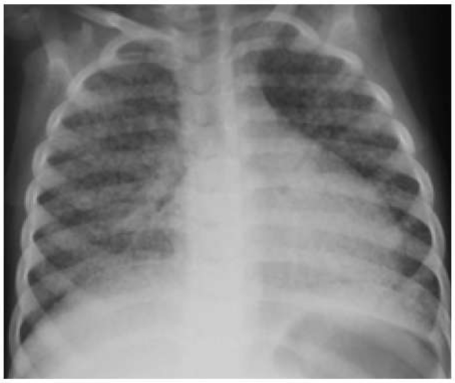

Miliary TB: typical bilateral diffuse micronodular pattern. Note differences to LIP X-ray above

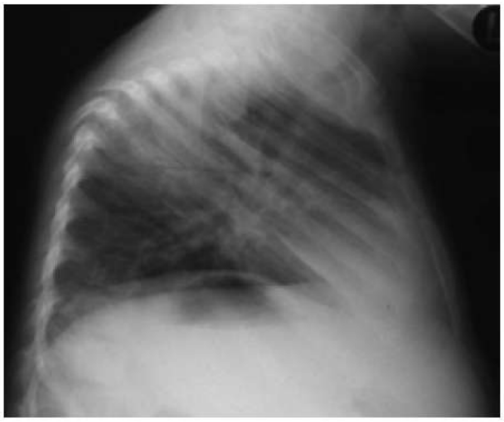

Spinal TB: collapse of thoracic vertebra causing angulation

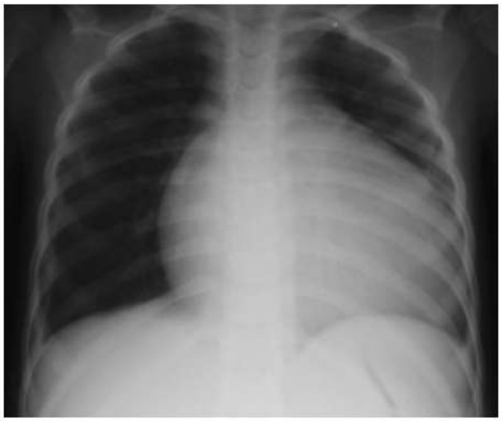

Pericardial TB: enlarged cardiac shadow. Ultrasound to differentiate from other causes of cardiac failure

3.5.3 Diagnostic algorithm in children

Figure 3:Pediatric diagnostic algorithm

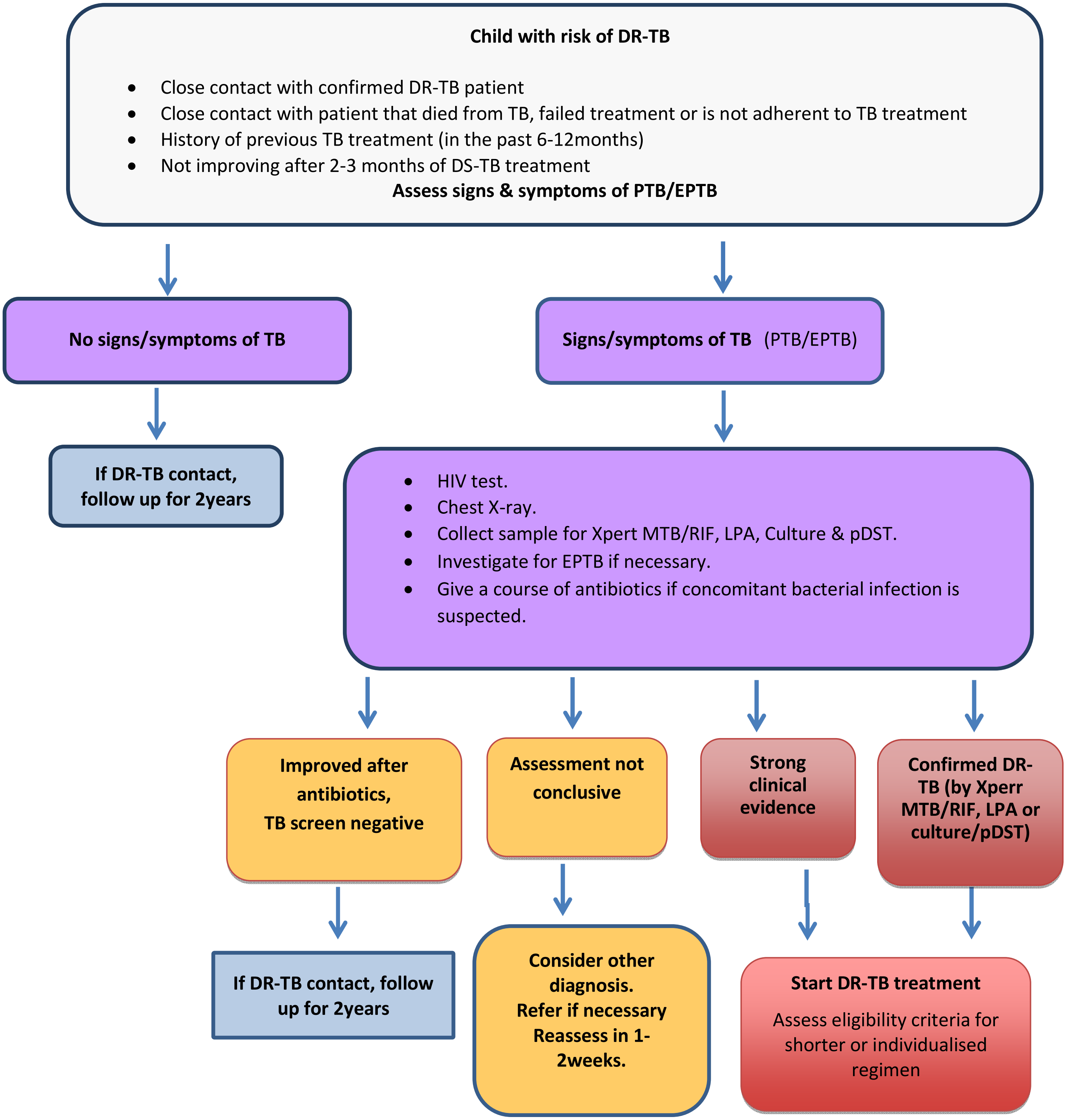

4. Treatment of drug-resistant tuberculosis

4.1 Eligibility criteria for starting DR-TB treatment regimens

a) Patients (adults and children) with confirmed rifampicin resistance
All patients enrolled in care in the implementation sites who have been diagnosed with RR-TB (RR/MDR/Pre-XDR/XDR TB) are eligible for the **DR-TB treatment options** as described in this guideline.

b) Other patients at high risk for rifampicin resistant TB to be considered for enrollment:
In the absence of bacteriological confirmation, the following are eligible for second-line treatment after considering all circumstances around the patient:

- Young children who are diagnosed with active TB, with a close contact, (especially a parent or caregiver) who has bacteriologically confirmed DR-TB
- PLHIV with active TB who are close contacts of known DR-TB patients
- EPTB who are close contact of a known DR-TB patient
- Failure of first-line TB treatment

4.2 Patient triage approach to select the appropriate DR-TB regimen

All patients with DR-TB are eligible to start DR-TB treatment without delay.A systematic approach must be followed to determine if the patient should be treated with **the shorter DR-TB regimen or an individualized DR-TB regimen**.

The approach includes clinical evaluation to determine the patient's risk of resistance or intolerance to FQ and/or SLI, and bacteriological testing of pre-treatment specimen to determine the strain's resistance to FQ and SLI drugs.

Before starting treatment,**two sputum samples must be sent**for second-line Line Probe Assay (SL-LPA) and for culture, as well as first line and second line phenotypic drug susceptibility tests (FL/SL pDST).Once the results are available, the initial regimen can then be adjusted if necessary to the appropriate regimen. [20]

Figure 4:Eligibility criteria for shorter and individualized treatment regimens [20]

RR-TB patient #

SEND SAMPLE FOR SL-LPA ANDCULTURE/pDST

EVALUATE PATIENT USING THE FOLLOWING ELIGIBILITY CRITERIA FOR SHORTER DR-TB TREATMENT REGIMEN

1) No evidence of resistance to FQ and/or SLI.
2) No contact with patient that has resistance to FQ/SLI.
3) No exposure to SLD for ≥ 1 month.
4) No known intolerance to drugs in the shorter regimen.
5) Not pregnant.
6) No EPTB *
7) No other risk of unfavorable outcome. **

**Initial treatment regimen**	Eligible<br>**Shorter DR-TB treatment regimen**	Ineligible<br>**Individualized DR-TB treatment regimen**

Regimen adjustmentbased on:<br>• **SL LPA (DST) results**<br>• **Treatment tolerance**	No resistance/intolerance to SLI and/or FQ	Resistance/intolerance to SLI and /or FQ	Resistance/intolerance to SLI and/or FQ	No resistance/intolerance to SLI and/or FQ
	**Continue** shorter DR-TB treatment regimen	**Change** to individualized DR-TBtreatment regimen	**Continue** individualized DR-TB treatment regimen	**Continue** treatment whilst **consulting** experts on potential regimen adjustment

#Includes patients with high risk of rifampicin resistance as contacts of RR/MDR-TB patients and failure of first line TB treatment.
* Non-severe forms of EPTB that can be eligible for the shorter treatment regimen include TB pleural effusion (adults and children) and TB lymph node (children).
** Risk of unfavorable outcome includes extensive or advanced TB disease (multiple cavities or extensive parenchymal damage)

If there is no risk of intolerance and/or resistance to FQ and/or SLI based on the clinical evaluation and/or the molecular DST (SL-LPA), the patient will start with the shorter regimen. If there is risk of intolerance/resistance to FQ and/or second line injectable and/or bacterial confirmation of drug resistance, pregnancy, EPTB (except pleural effusion in adults and children and TB Lymph node in children) or other risk factors for poor treatment outcome (such as severe TB disease), the patient shall start with an individualized treatment regimen [20].

Once the genotypic second line (SL-LPA) DST results become available, the initial treatment regimen should be re-evaluated. In this case, there are 5 options (see Figure 4):

1. For patients that started with the shorter regimen and genotypic second line DST results reveal no additional resistance to FQ and/or SLI and are tolerating well the drugs the shorter regimen can be continued
2. For patients that started with the shorter regimen and genotypic results show additional resistance to FQ and/or SLI, the patient should switch to (starting from the beginning of the treatment duration) an individualized treatment regimen (based on the culture and DST results). For patients with intolerance/toxicity to any drug, it can be considered to stop the offending drug and substitute with a new or repurposed TB drug if necessary (the duration and composition of the regimen should be guided by DST results).
3. For patients that started with an individualized regimen based on **resistance** to FQ and/or SLI which is confirmed by the culture and DST results, they should continue the individualized treatment regimen.
4. For patients that started with individualized regimen based on **intolerance** to FQ and/or SLI, the regimen should be re-evaluated and adjusted if needed based on the phenotypic DST results.
5. For patients that started with an individualized regimen based on **resistance** for FQ and/or SLI which is not confirmed by phenotypic DST results, they should continue the treatment whilst consulting expert on potential regimen adjustment based on DST results and clinical status. [20]

Ideally, all DR-TB patients are to be tested for resistance to FQ and/or SLI before starting any DR-TB treatment, and efforts should be made to ensure that rapid molecular DST for FQ and SLI is available for all DR-TB patients with prompt turn-around time of results.

4.3 DR-TB Treatment Regimens

4.3.1 Shorter DR-TB regimen (STR)

4.3.1.1 Patients eligible for the shorter DR-TB treatment regimen

Patients who have **low risk of additional resistance to FQs and/or SLIs**(patients without known contact with pre-XDR-/XDR-TB patient and/or without previous exposure to SLDs for more than one month) or with DST results excluding additional resistance to FQ and/or SLI will be treated with the shorter DR-TB treatment regimen.

Children and HIV infected patients with clinically diagnosed TB who have not been previously treated with SLDs and with low-risk of additional resistance to FQ and/or SLI who have been in close contact with patients with RR-/MDR-TB should also be considered for treatment with a shorter regimen as described in this guideline.

4.3.1.2 Exclusion criteria for the shorter DR-TB regimen

1) Confirmed resistance to FQ and/or SLI.
2) Contact with patient that has resistance to FQ/SLI.
3) Exposure to SLD for ≥ 1 month. **Patients already on treatment with a conventional DR-TB treatment regimen for more than one month cannot be switched to the shorter DR-TB treatment regimen.**
4) Known intolerance to drugs in the shorter regimen.
5) Pregnancy.
6) Extra-pulmonary TB(except lymph node TB in children and pleural TB in children and adults):
Specific recommendations on EPTB cannot yet be made by WHO, as studies were limited to patients with pulmonary disease. The inclusion of patients with EPTB for treatment with the shorter DR-TB regimen is upon the respective NTP's discretion. It is suggested that non-severe forms of EPTB such TB pleural effusion (adults and children) and TB Lymph Nodes (children) could be eligible for treatment with the shorter DR-TB regimen.
7) Other risks of unfavorable outcome including extensive or advanced TB disease (multiple cavities or extensive parenchymal damage).

4.3.1.3 Regimen design for the shorter DR-TB regimen

The shorter regimen is given as a standardized regimen with little or no room for customization with substitutions or switches during treatment.

- The **intensive phase** consists of **Km**[1]**, high dose Mfx**[2]**, Cfz, Eto**[3]**, Z, E and H**^{h} daily for **fourmonths.**
- The **criteria to shift to continuation phase** is based on smear conversion and clinical response. The patient should have smear negative results and be clinically improving. All attempts should be made to send monthly samples for culture and trace results. It is important to have SL LPA, culture and SL phenotypic DST results available to exclude resistance to FQ and SLI (though SL LPA test when performed in smear negative samples can have uninterpretable results).
- If the smear conversionis not achieved at month four, the intensive phase shall be extended to a maximum total duration of six months (until smear conversion and one negative culture result). Then Km (Am or Cm) may be given thrice-weekly (on alternate days), from the fourth month onwards (fifth and sixth months).
- If the patient remains smear and/or culture positive at six months, will be declared as a treatment failure and switched to an individualized regimen.

---

[1] Am and Cm are acceptable alternatives
[2]. In children <14 Kg Levofloxacine is an acceptable alternative (due to bad palability of Moxifloxacine)
[3] Pto is an acceptable alternative

- Failure declaration and a switch to an individualized treatment will be considered earlier than six months in patients with clear lack of response (clinically, smear grading, culture). The decision to switch earlier must be made by the expert committee of state consilium (and if required national consilium.
- The **continuation phase** consists of high dose **Mfx, Cfz, E and Z** for a fixed duration of **five months.**

Shorter Regimen

4-6 Km-Mfx-Cfz-Pto-Z-E-*H*[h] / 5 Mfx-Cfz-E-Z

Add vitamin B6 100 mg

Table 2: Short course regimen drug dosages for adults and adolescents > 30 kg

Dosage of medicines to use in the WHO recommended shorter DR-TB regimen for adults:

Drug	Weight Group		
	Less than 30 kg	30 – 50 kg	More than 50 kg
Moxifloxacin	400 mg	600 mg	800 mg
Clofazimine	50 mg	100 mg	100 mg
Prothionamide	250 mg	500 mg	750 mg
Pyrazinamide	1000 mg	1500 mg	2000 mg
Ethambutol	800 mg	800 mg	1200 mg
Isoniazid	300 mg	400 mg	600 mg
Kanamycin*	15 mg / kg body weight (maximum 1 gm)		

*****For adults over 59 years of age, the dose will be reduced to 10 mg/kg (maximum dose 750 mg)

Dosage of medicines to use in the WHO recommended shorter regimen for children and adolescents:
Given below together with individualized regimen.

4.3.2. Individualized regimen

Patients with DR-TB who are not eligible for treatment with the shorter regimen will be treated with the individualised regimen. The total duration is 20 months or longer.**Regimen design for individualised treatment**

1) The regimen will be designed based on the patient's most recent DST results and history of previous drug use and/or exposure (see Table ---);
2) Standard duration of the intensive phase will be at least 6 months and duration of the continuation phase will be at least 14 months.

The duration of the intensive phase (injectable agent) depends on the number of effective drugs in the regimen, patient response to treatment and tolerability. At least 5 effective drugs during the

intensive phase (one chosen from Group A, one from Group B and at least two from Group C. If the minimum number of TB medicines cannot be composed as above, an agent from D2 and other agents from Group D3 should be added to bring the total to five. At least 3 effective drugs are strongly advised to be used after culture conversion (until the end of treatment)

3) Bdq or Dlm will be used for 6 months and 12 months as and when required. The use of Bdq or Dlm can be extended by DR-TB expert committee in cases where the remaining regimen is insufficient (less than 3 effective drugs) and treatment tolerability is good.

Note

- For patients enrolled for treatment with regimens containing new drugs (Bdq or Dlm) informed consent should be obtained as per WHO guidelines.
- For HIV-infected patients, ART will be prescribed within the first eight weeks of DR-TB treatment initiation. For patients on ART, Dlm (if available) is preferable because of the drug-drug interaction between Bdq and Efavirenz (and Lopinvir/Ritonavir).
- Criteria to include new drugs Bdq or/and Dlm in the Individualised regimen are: DR-TB patient with additional resistance or intolerance to Fluoroquinolone and/or SLI (Pre-XDR, XDR), and those with extensive lesions and advanced disease and others with likelihood or poorer outcome. If indicated, Dlm or Bdq can be used also for children with proper safety measures. However, for children more than 6 years old (>20 Kg) and adolescents,Dlm should be the drug of choice.
- The patient will be provided with materials in local languages that explain DR-TB treatment procedures.

Additional information on contraindications and precautions for SLDs is provided in the Annex

Table 3: Adapted Individualised regimens for DR-TB in Nigeria

**Types of DR-TB**	**Phases of treatment**		**Total duration**
	**Intensive**	**Continuation**	
**Pre-XDR TB (resistant to second line injectable)**	6 Mfx-Bdq-Lzd-Cfz-PAS-H^h-Z	14 Bdq(6)-Lzd-Cfz-PAS-Z	20 months
**Pre-XDR TB (resistant to fluoroquinolones)**	6 Cm/Km-Bdq-Lzd-Cfz-PAS-H^h-Z	14 Bdq(6)-Lzd-Cfz-PAS-Z	20 months
**XDR-TB**	6 Cm/Dlm-Bdq-Lzd-Cfz-PAS-H^h-Z	14 Bdq(6)-Lzd-Cfz-PAS-Z	20 months

Dosage and administration

Table 4: Weight-based oral anti-TB drug daily dosing in adults ≥30 kg[a]

**Drugs**	**Daily dose**	**30-35 Kg**	**36-45 Kg**	**46-55 Kg**	**56-70 Kg**	**>70 Kg**
Isoniazid	4-6 mg/kg once daily	150 mg	200 mg	300 mg	300 mg	300 mg
Rifampicin	8-12 mg/kg once	300 mg	450 mg	450 mg	600 mg	600 mg

	daily					
Pyrazinamide	20-30 mg/kg once daily	800 mg	1000 mg	1200 mg	1600 mg	2000 mg
Ethambutol	15-25 mg/kg once daily	600 mg	800 mg	1000 mg	1200 mg	1200 mg
Rifabutin	5–10 mg/kg once daily	300 mg	300 mg	300 mg	300 mg	300 mg
Levofloxacin	750–1000 mg once daily	750 mg	750 mg	1000 mg	1000 mg	1000 mg
Moxifloxacin	400 mg once daily	400 mg	400 mg	400 mg	400 mg	400 mg
Ethionamide	500–750 mg/day in 2 divided doses	500 mg	500 mg	750 mg	750 mg	1000 mg
Prothionamide	500–750 mg/day in 2 divided doses	500 mg	500 mg	750 mg	750 mg	1000 mg
Cycloserine	500–750 mg/day in 2 divided doses	500 mg	500 mg	500 mg	750 mg	750 mg
p-aminosalicylic acid	8 g/day in 2 divided doses	8 g	8 g	8 g	8 g	8–12 g
Bedaquiline	400 mg once daily for 2 weeks then 200 mg 3 times per week					
Delamanid	100 mg twice daily (total daily dose = 200 mg)					
Clofazimine	200–300 mg daily (2 first months) then reduce to 100 mg daily (alternative dosing 100 mg daily)					
Linezolid	600 mg once daily	600 mg	600 mg	600 mg	600 mg	600 mg
Amoxicillin/Clavulanic acid 7/1	80 mg/kg/day in 2 divided doses	2600 mg	2600 mg	2600 mg	2600 mg	2600 mg
Amoxicillin/Clavulanic acid 8/1	80 mg/kg/day in 2 divided doses	3000 mg	3000 mg	3000 mg	3000 mg	3000 mg
High-dose isoniazid	16–20 mg/kg once daily	600–1000 mg	1000–1500 mg	1500 m	1500 m	1500 m
Imipenem / Cilastatin	1000 imipenem/1000 mg cilastatin twice daily					
Meropenem	1000 mg three times daily (alternative dosing is 2000 mg twice daily)					

a. Adapted from ‘Companion handbook to the WHO guidelines for the Programmatic Management of Drug-resistant Tuberculosis - 2014’.

Table 5: Weight-based injectable anti-TB daily dosing in adults ≥30 kg[a]

DRUGS	DAILY DOSE	30–33 KG	34–40 KG	41–45 KG	46–50 KG	51–70 KG	>70 KG
**Kanamycin**	15–20 mg/kg once daily	500 mg	625 mg	750 mg	875 mg	1000 mg	1000 mg
**Amikacin**	15–20 mg/kg once daily	500 mg	625 mg	750 mg	875 mg	1000 mg	1000 mg
**Capreomycin**	15–20 mg/kg once daily	500 mg	600 mg	750 mg	800 mg	1000 mg	1000 mg

a. Adapted from 'Companion handbook to the WHO guidelines for the Programmatic Management of Drug-resistant Tuberculosis - 2014'.

Weight based dosing in children for shorter as well individualized regimen:

General considerations:

- Anti-TB drugs should be dosed according to weight and adjusted regularly as weight increases during treatment.
    - When a liquid formulation is available, it should be used for patients less than 15 kg.
    - Most second-line TB drugs do not have paediatric liquid or tablet formulations, so it may be necessary to split the pills in order to approximate the correct dose. To split tablets into 0.75, it is suggested to split the tablet in half and then split a half tablet in half. Discard the smaller quarter tablet and give the child a half tablet plus the remaining quarter tablet.
    - Doses of most anti-TB drugs have not been established for children below 5 kg, but often the potential benefit outweighs the risks. In such cases, the child should be dosed as close to the middle of the mg/kg range as possible.

Table 6: Weight based dosing tables:<u>All tables with dosages for children are grouped as one.</u>

Isoniazid (7–15 mg/kg for patients less than 30 kg; maximum dose 300 mg daily):

**Body weight Kg**	**50 mg per 5 ml oral solution**	**100 mg tablet**	**300 mg tablet**
5	5 ml	0.5 tab	
6	6 ml	1.0 tab	
7	7 ml	1.0 tab	
8	8 ml	1.0 tab	
9	9 ml	1.0 tab	
10	10 ml	1.5 tab	
11	11 ml	1.5 tab	
12	12 ml	1.5 tab	
13	13 ml	2.0 tab	
14	14 ml	2.0 tab	
15	15 ml	2.0 tab	

16-20		2.0 tab	
21-30			1.0 tab

Notes:

The table shows the "regular" dose for children, not high-dose isoniazid, which is rarely used in children.

Children at risk for peripheral neuropathy (e.g. malnutrition or HIV co-infection) should also receive pyridoxine 5–10 mg/day.

Rifampicin (10–20 mg/kg for patients less than 30 kg; maximum dose 600 mg daily):

Body weight Kg	100 mg per 5 ml oral suspension	150 mg tablet	300 mg tablet
5	4 ml	0.5 tab	
6	5 ml	0.5 tab	
7	5 ml	0.5 tab	
8	6 ml	1.0 tab	
9	7 ml	1.0 tab	
10	8 ml	1.0 tab	
11	9 ml	1.0 tab	
12	10 ml	1.0 tab	
13	10 ml	1.5 tab	
14	11 ml	1.5 tab	
15	12 ml	1.5 tab	
16-30			1.0 tab

Notes:

Oral solution is preferred for children less than 15 kg.

Ethambutol (15–25 mg/kg, maximum dose 1200 mg daily):

Body weight Kg	100 mg tablet	400 mg tablet
5-7	1.0 tab	
8-13	2.0 tab	
14-17	3.0 tab	
18-26		1.0 tab
27-30		1.5 tab

Notes:

Older children over 16 kg can use the adult 400 mg tablet in combination with the 100 mg tablet to reduce pill count.

Pyrazinamide:

Pyrazinamide (30–40 mg/kg for patients less than 30 kg; maximum dose 2000 mg daily):

Body weight Kg	400 mg tablet
5-7	0.50 tab
8-9	0.75 tab
10-14	1.00 tab

15-20	1.50 tab
21-27	2.00 tab
28-30	2.50 tab

Pyrazinamide (30–40 mg/kg for patients less than 30 kg; maximum dose 2000 mg daily):

**Body weight Kg**	**500 mg tablet**
5-6	0.25 tab
7-9	0.50 tab
10-11	0.75 tab
12-18	1.00 tab
19-25	1.50 tab
26-30	2.00 tab

Notes:

Pyrazinamide comes in either 400 mg or 500 mg tablets. Tablets are big enough to split into quarters.

Injectable anti-TB drugs:

**Drug**	**Daily dose**	**Maximum daily dose**
Kanamycin	15–30 mg/kg once daily	1000 mg
Amikacin	15–30 mg/kg once daily	1000 mg
Capreomycin	15–30 mg/kg once daily	1000 mg

Example: Injectable dose calculation for a child weighing 6.9 kg

- Calculate the low and high doses for the child's weight. For kanamycin:
- Low dose: 15 mg/kg x 6.9 kg = 103 mg
- High dose: 30 mg/kg x 6.9 kg = 207 mg
- Choose a convenient dose between the two numbers.

- Select a dose between the two numbers and toward the higher number. In this case, 200 mg is a convenient dose.
    - Calculate the number of ml to draw up in the syringe based on the mg/ml concentration of the preparation.

Levofloxacin:

5 years and under: 15–20 mg/kg split into two doses (morning and evening).

Over 5 years: 10–15 mg/kg once daily:

**Body weight Kg**	**Under 5 years (250 mg tablet)**	**More than 5 years (250 mg tablet)**
10-15	0.50 tab twice daily	-
16-23	0.75 tab twice daily	1.0 tab once daily
24-30	1.00 tab twice daily	1.5 tab once daily

Notes:

Levofloxacin is dosed twice daily for children 5 years of age and under (total daily dose: 15–20 mg/kg/day) and once daily for children over 5 years of age (total daily dose: 7.5–10 mg/kg/day). This is done because children under 5 years metabolize levofloxacin faster than those older than 5 years. Once daily dosing at 15 mg/kg resulted in adequate serum concentrations for children less than 5 years in at least one programme and can be used as an alternative if twice daily dosing is not programmatically possible.

Moxifloxacin (7.5–10 mg/kg):

**Body weight Kg**	**400 mg tablet**
10-17	0.25 tab
18-30	0.50 tab

Cycloserine (10–20 mg/kg):

**Body weight Kg**	**250 mg capsule**	**1 capsule in 10 ml water**
5	0.25 cap	2.5 ml
6-9	0.50 cap	5.0 ml
10-11	0.75 cap	7.5 ml
12-22	1.00 cap	10.0 ml
23-30	2.00 cap	-

Notes:
For older children who cannot swallow capsules, the capsules can be opened and dissolved in 10 ml water to aid administration.

Prothionamide/Ethionamide (15–20 mg/kg):

**Body weight Kg**	**250 mg tablet**
5-10	0.5 tab
11-18	1.0 tab
19-24	1.5 tab
25-30	2.0 tab

Para-amino salicylic acid (PAS):

PAS (200–300 mg/kg for patients less than 30 kg):

**Body weight Kg**	**PASER (Jacobus)**
5	500 mg twice daily
6-7	750 mg twice daily
8-10	1000 mg twice daily
11-14	1500 mg twice daily
15-18	2000 mg twice daily
19-22	2500 mg twice daily
23-26	3000 mg twice daily
27-30	3500 mg twice daily

PAS (200–300 mg/kg for patients less than 30 kg):

Body weight Kg	Mono-PAS 9.2 g (Macleods)
5-6	1.5 g twice daily
7-8	2.0 g twice daily
9-13	3.0 g twice daily
14-18	4.0 g twice daily
19-24	6.0 g twice daily
25-30	8.0 g twice daily

Notes:

PASER is stable for up to eight weeks at 40°C and 75% humidity and therefore can be distributed to the patient on a monthly basis in most environments with no cold chain. If storage of longer than eight weeks is needed, refrigeration below 15°C is required.

PASER comes with a dosage scoop graduated in milligrams, and MonoPAS 9.2 g comes with a measuring spoon graduated in grams.

Group 5 anti-TB drugs:

Drug	Daily dose	Maximum daily dose
Linezolid (Lzd)	10 mg/kg given three times daily (pyridoxine should also be given)	600 mg
Clofazimine (Cfz)	Limited data, but 1 mg/kg once daily has been given	200 mg
Amoxicillin/Clavulanic acid (Amx/Clv)	80 mg/kg (based on the amoxicillin component) in two divided doses	4000 mg amoxicillin and 500 mg clavulanic acid
Meropenem (Mpn)	20–40 mg/kg intravenous every eight hours	6000 mg
Imipenem/cilastatin (Imp/Cln)	Meropenem is preferred in children	
Bedaquiline (Bdq)	Dose not yet determined in children	
Delamanid (Dlm)	Dose not yet determined in children	

Notes:

Most of the Group 5 drugs, except amoxicillin/clavulanic acid and linezolid, have limited experience with dosing in children. The data on long-term use of all the Group 5 drugs in children is also limited.

a. Adapted from ‘Companion handbook to the WHO guidelines for the Programmatic Management of Drug-resistant Tuberculosis - 2014’.

Use of delamanid in children & adolescents in RR/MDR-TB as per WHO guideline 2016:

Based on Guideline Development Group (GDG) recommendations, WHO recommends that delamanid may be added to the WHO-recommended longer regimen in children and adolescents (6

– 17 years) with multidrug- or rifampicin-resistant TB (MDR/RR-TB) who are not eligible for the shorter DR-TB regimen, under specific conditions.

The recommended dose of delamanid in children (aged 6–11 years) is 50 mg BID for 6 months, and in adolescents (aged 12–17 years) it is 100 mg BID for 6 months. Because bioavailability was found to be higher when given after a standard meal, delamanid should preferably be delivered after a meal. Given that TB regimens are generally administered once a day, any observation of treatment needs to be adapted to ensure supervision of the BID intake of delamanid in patients on this medication.

Because delamanid is shown to cause prolongation of the QT interval, children with a QTcF > 500 msec should not receive the drug.

Children and adolescent MDR-TB/RR-TB in whom delamanid may have a particular role include those with the following:

- higher risk for poor outcomes (e.g. drug intolerance or contraindication, extensive or advanced disease);
- additional resistance to fluoroquinolones or injectable drugs; or
- XDR-TB.

While patients with exclusively extra-pulmonary disease were not included in the delamanid trials, there is no absolute contraindication for its use in such patients, and inclusion may be considered where any potential harms that delamanid may cause are offset by the benefits expected.

4.4 Treating mono and poly-drug resistant TB

If the patient has mono- or poly-drug resistant TB with resistance to rifampicin (susceptible to isoniazid), they should receive the same regimens as RR/MDR-TB (shorter or individualized according to eligibility criteria), with isoniazid high dose included.
If the patient has mono- or poly-drug resistant TB with resistance to isoniazid (susceptible to rifampicin), care should be taken to evaluate the medical history for possible amplification of resistance which may have developed, but may not be apparent from the laboratory results.
As such, treatment for mono- and poly-drug resistant TB should never rely solely on DST results.It is important to assess the history of previous TB treatment, contact history, risk of amplification of resistance, extension of the disease, and patient's condition.
There is no updated international recommendation on the treatment of isoniazid mono- and poly-drug resistance at the time of developing these guidelines. For isoniazid poly-drug resistance, MDR-TB regimen can be considered. For isoniazid mono-resistance, the patient summary can be referred to the national DR-TB committee for guidance.

4.5 Adjuvant therapy

- Vitamin B6 (pyridoxine) preventive therapy at a dose of 100mg/day up to 150 mg/day should be given to all patients receiving isoniazid, cycloserine or linezolid to minimize peripheral neuropathy, neurological adverse event and myelosuppression.

- Corticosteroids (Prednisolone 1 mg/kg and gradually decreasing by 10 mg per week when a long course is indicated) may be used in the following conditions:
    - TB Meningitis (and other compromise of CNS).
    - TB Pericarditis.
    - Immune reconstitution inflammatory syndrome (IRIS).

4.6 Role of surgery

Patients with DR-TB with the following conditions should be considered for surgical intervention:

- Patients who remain smear positive, while on fully monitored treatment for more than six months*and*
- Have resistance to more than two of medicines*and*
- Have localized pulmonary disease.

The most common operative procedure in patients with pulmonary DR-TB is resection surgery. Generally, at least two months of therapy should be given prior to resection surgery to decrease the bacterial load in the surrounding lung tissue. Even with successful resection, an additional 12-24 months of chemotherapy should still be given.

5. Treatment of DR-TB in special conditions and situations

5.1 Pregnancy

- **Ideally women should avoid getting pregnant during DR-TB treatment**.A pregnancy test and counselling with partner at baseline is mandatory and effective use of contraceptives is advocated (preferably barrier contraception or intra-uterine device or IUDs).
- Treatment of DR-TB during pregnancy should be **individualized**.
- **Involve the patient (and if possible, the partner) in the decision making** for the regimen.
- Most pregnant patients should be started on treatment as soon as the diagnosis is made, if there is life-risk to the mother. However, since the majority of teratogenic effects occur in the first trimester, **treatment may be delayed until the second trimester if the patient is stable.**
- ***Treat with four oral second-line anti-TB drugs*** which are likely to be highly effective against the infecting strain ***plus pyrazinamide***.
- **Avoid injectable agents**. Aminoglycosides can be particularly toxic to the developing fetal ear. **Cm** may also carry a risk of ototoxicity but is the injectable drug of choice if an injectable agent cannot be avoided because of an immediate life-threatening situation of the mother resulting from DR-TB. The option of using **Cmthree times aweek** from the start can be considered.
- **Avoid Pto (Eto)** as it can increase the risk of nausea and vomiting associated with pregnancy, and teratogenic effects have been observed in animal studies.
- There is no evidence of safety ofCFZduring pregnancy or lactation. Avoid unless absolutely necessary.
- Despite limited data on safety and long-term use of ***FQ, Cs, PAS and Amx/Clv*** in pregnancy, they are considered safe drugs for DR-TB treatment during pregnancy.
- There may not be a clear transition between the intensive and continuation phase, and the injectable agent can be given for three to six months postpartum even in the middle of treatment. If the patient is doing well and past the normal eight-month period for the injectable agent, there is no need to add it.

Note: new data and updates regarding DR-TB treatment in pregnancy are emerging. Contact national DR-TB committee for latest information.

There are two possible scenarios in DR-TB and pregnancy:

1) **The patient is already on DR-TB treatment and becomes pregnant.** In this case if still on the intensive phase, stop the injectable and give another second-line oral drug during the rest of treatment.

2) **A pregnant patient is diagnosed with DR-TB.**
   Assess the patient individually:
   - Month of pregnancy,
   - Severity of the disease,
   - Risk of FQ or SLD resistance.

   Laboratory results:
   - Xpert MTB/RIF
   - First and second line LPA

- Culture and phenotypic DST.

Consider the possibility of delaying the treatment until after the second trimester if the patient is stable and with minimum disease.

Regimen selections in pregnant patients must be referred to the expert committee. A possible regimen could be:Mfx/Lfx + Cm(three times a week) + Cs +PAS + E + Z.

5.2 Breast feeding:

The general recommendation is that women can breast feed while they are on DR-TB treatment. Most of the drugs are passed via the breastmilk, but the concentrations are very low.
The notable exceptions are Clofazimine and Bedaquiline which both accumulate in the fatty tissues of the breast and are excreted in the milk (the baby may have discoloration of skin by clofazimine).

When the mother is sputum smear or culture-positive, family members may be asked to assist with the care of the infant until she becomes sputum smear-negative. When the mother and infant are together, this common time should be spent in well-ventilated areas or outdoors. The mother should be offered N-95 respirator during the intensive phase.

There is need to closely monitor the infant for the signs and symptoms of TB during the following two years.

5.3 Children

For dosage of DR-TB medicines in children, refer to pages 29 to 34 of this guideline [4], [9],[10],[11],[14], [15].

5.3.1 Considerations for DR-TB treatment in children

- Treatment should be based on **contact DST** or DST of a sample from the child when it is available.
- The same inclusion criteria for adults should be applied for children being considered for the shorter regimen or individualized regimen, though the documentation of fluoroquinolone or injectable resistance, as well as history of prior treatment with 2nd line medication may be taken from the adult index case.
- It is important to **monitor the weight monthly** and to **adjust the dosages accordingly**.
- All **drugs should be dosed** at the **higher end of the recommended range** when it is possible (with the exception of Ethambutol which should be dosed at the lower range: 15 mg/Kg)
- Most second-line anti-TB drugs do not have pediatric liquid or tablet formulations, so it may be necessary to split the pills in order to approximate the correct dose.
- Ideally the dose of the injectable is between 15-30 mg/kg/day. Consider using the injectable five times per week.
- Ensure that once treatment starts that it is completed; “trial of TB treatment” should not be used as a diagnostic tool. **Adherence to the full course of treatment** should be emphasized and reinforced.
- Identify a caregiver should be the **DOT supporter** for all ages, including adolescents. Health care workers are responsible for ensuring DOT.
- Co-morbidities should be adequately managed.

- For all **HIV-infected children**:
  - Cotrimoxazole preventive therapy (CPT).
  - Commence antiretroviral therapy (ART) within 2-8 weeks of starting anti-TB treatment.
  - Address disclosure as soon as possible (children above 6 years).
- Provide **adequate nutritional support** for all malnourished children (ready to use therapeutic food, therapeutic milk when necessary).
- To achieve the average dose, clofazimine may be given every other day, or even every third day. (Clofazimine capsules are very difficult to open, they color the hands and can only be diluted in oil).
- **Delamanid** is recommended by WHO for children **above the age of 6 (weighing more than 20 kg**) and adolescents who are not eligible for the shorter regimen. [16]
- For infants and children under 6 years of age that need a regimen with new drugs, consult the national DR-TB committee.

5.4Liver disease

- Eto/Pto, H^h, PAS, and Bdq might be hepatotoxic; use with caution in chronic stable liver disease with close monitoring of liver function tests.
- Avoid pyrazinamide in patients with underlying chronic liver disease.
- Avoid other potentially hepatotoxic non-tuberculous agents in patients with underlying chronic liver disease.
- If DR-TB is to be treated concurrently with acute hepatitis, a combination of four non-hepatotoxic drugs should be used.
- Monitor LFTs at the baselines and monthly. Stop all drugs if the ALT/AST are more than five times the upper limit (or more than 3 times with jaundice) and investigate accordingly.
- No formal studies of drug-drug interactions between the Hepatitis C protease inhibitors and the second-line anti-TB drugs have been done; if a patient has stable liver function, treatment of DR-TB should be initiated first.
- Alcohol use might substantially exacerbate liver disease in some settings, although studies have found that no increased risk of hepatic adverse events exists in people with DR-TB.

5.5Renal disease

- Care should be taken in the administration of second-line anti-TB medicines in patients with renal insufficiency.
- The formula to calculate the estimated creatinine clearance rate (CrCl) is as follows:

$$\text{Estimated clearance} = \frac{(140 - \text{Age}) \times \text{Body weight (Kg)} \times \text{constant}}{\text{Serum creatinine } (\mu mol/l)}$$

Constant = 1.23 for males, 1.04 for females

*If creatinine is reported in conventional units (**mg/dl**), it can be **converted**to (**µmol/l**) bymultiplying by **88.4***

(For example, a creatinine = 1.2 mg/dl is equivalent to: 1.2 x 88.4 = 106.1 µmol/l)

Normal values are*97 to 137ml/min for males* and *88 to 128ml/min for females.[4]*

- Frequency and dosage of anti-TB drugs should be adjusted if creatinine clearance is below 30 ml/min (Refer to Annex C, table 18)
- Bdqor Dlmcan be used to substitute second-line injectable drugs in patients with significant pre-existing or aminoglycoside-related renal dysfunction.

- Bdqor Dlm does not require any dose adjustments for patients with mild to moderate renal dysfunction (i.e., not on dialysis).

5.6Diabetes Mellitus

- Diabetes Mellitus (DM) increases the risk of poor outcomes and can potentiateadverse effects e.g., renal dysfunction, peripheral neuropathy and visual disturbances.
- Prompt diagnosis and initiation on effective treatment is crucial in patients with diabetes.
- DM must be monitored closely throughout the treatment of DR-TB using fasting blood sugar testing twice a month and whenever indicated.
- Oral hypoglycemic drugs are not contraindicated but dosages may need to be increased during treatment with anti-TB drugs.
- Eto/Pto tend to make insulin control in diabetes more difficult and can result in hypoglycemia and poor glucose regulation
- Creatinine and potassium levels should be monitored frequently, often weekly for the first month and then at least monthly thereafter.
- No anti-TB drug is contraindicated in diabetic patients.

5.7 Seizure disorders

- Cycloserine (Cs) should be avoided in patients with poorly controlled seizure disorders. However, in cases where Csis a crucial component of the treatment regimen, it can be given and the anti-seizure medication can be adjusted as needed to control the seizure disorder.
- If the patient is on Cs or isoniazid, provide pyridoxine prophylaxis (50 mg of vitamin B6 for every 250 mg of Cs).
- Caution must also be exercised when giving isoniazid or imipenem as it may trigger seizures.
- The use of isoniazid and rifampicin may interfere with some commonly used anti-seizure medications. Interactions should be checked before their use.
- Seizures that present for the first time during anti-TB therapy are likely to be the result of an adverse effect of one of the anti-TB medicines, particularly cycloserine or isoniazid.
- Other causes of seizure should be considered especially in HIV positive patients (cryptococcal meningitis, cerebral toxoplasmosis, tuberculoma, malignancies etc).

5.8 Psychiatric disorders

- Patients with a history of overt psychiatric illness should be evaluated by a health care worker with psychiatric training, preferably before starting DR-TB treatment and at any point if symptoms re-appear. Any psychiatric illness identified should be fully addressed.
- There is a high baseline incidence of depression and anxiety in patients with DR-TB, often related to the chronicity of the condition and the socioeconomic stress factors associated with the disease.
- Medical treatment, individual counseling and/or group therapy may be necessary to manage patients suffering from a psychiatric condition or an adverse psychiatric effect caused by medication. Group therapy provides a supportive environment for DR-TB patients and should be provided for all patients, including those without psychiatric conditions. Every facility that treats DR-TB patients is encouraged to conduct regular support group sessions for these patients.

- The adverse effects of Cycloserine may be more prevalent in psychiatric patients. Close monitoring is recommended if it is used in patients with psychiatric disorders.
- All facilities treating DR-TB should have an organized system for psychiatric emergencies (e.g., psychosis, suicidal tendencies, etc.).

5.9 Psycho-active substance dependence

- Patients with substance dependence disorders, including alcohol dependence, should be offered treatment for their addiction. Consultation with social workers, psychiatrists and/or the HCWs of drug rehabilitation centers is encouraged to formulate the treatment plan.
- Complete abstinence from alcohol or other substances should be strongly encouraged, although active consumption is not a contraindication for anti-TB treatment.
- If DR-TB treatment is repeatedly interrupted because of the patient's dependence to alcohol or psychoactive substances, TB treatment may be suspended until measures to ensure adherence have been established.
- Good DOT gives the patient an opportunity to interact with and get support from healthcare providers, which often allows treatment completion even in patients with substance dependence. In patients who are dependent on alcohol or other substances, cycloserine may have a higher incidence of adverse drug reactions(e.g., a predisposition to seizures). However, if cycloserine is considered important to the regimen, it should be used and the patient must be closely observed for adverse effects, which if any should be adequately treated.

6. HIVand drug-resistant tuberculosis.

6.1 General considerations in the management of DR-TB and HIV co-infection

HIV is a powerful risk factor for all forms of TB (drug-susceptible and drug-resistant TB). [4]

HIV infection is a significant challenge for the prevention, diagnosis, and treatment of DR-TB, and mortality rates among HIV-infected patients with DR-TB are relatively high.

Xpert MTB/RIF should be used as initial diagnostic test for all HIV positive patients with presumed TB. If available, perform culture and phenotypic DST at start of TB therapy. [4]

All patients with presumed or diagnosed DR-TB should be offered an HIV test and if positive, start co-trimoxazole preventive therapy (CPT) and ART regardless of CD4 count. [4] Those that are HIV negative should be educated on preventive measures and offered condoms.

If the patient is already known to be HIV positive, CD4 and VL should be monitored. For patient diagnosed ART failure it is important to switch to second line ART as early as possible.

Essential components in the management of DR-TB in HIV co-infected persons include the following:

- Early diagnosis of DR-TB and HIV.
- Early diagnosis of ART failure for patients already on ART.
- Aggressive and prompt initiation of appropriate treatment with both SLDs and ART.
- Consideration of overlapping toxicities and drug-drug interactions.
- Early diagnose and management of adverse drug reactions (ADR).
- Early diagnosis and management of other opportunistic infections and IRIS.
- Additional nutritional support.
- Psychosocial support and counseling for adherence to both treatments.
- Provision of integrated TB/DR-TB and HIV services
- Strong infection control measures.

6.2 Clinical features and diagnosis of DR-TB in HIV infected patients

The diagnosis of TB (including RR/MDR-TB and XDR-TB) in PLHIV is more difficult and may be confused with other pulmonary or systemic infections. The presentation is more likely tobe extra-pulmonary or sputum smear-negative than in TB patients not infected with HIV, especially as immuno-suppression advances. This can result in misdiagnosis or delays in diagnosis,and in turn, higher morbidity and mortality. [4]

It is important to emphasize the use of clinical criteria and radiography especially for seriously ill patients, and the prompt utilization of all available investigations including molecular tests (Xpert MTB/RIF), FL and SL LPA, culture/phenotypic DST [4]. For patients with CD4 < 100cells/µl or those who are seriously ill, perform urine lateral flow lipoarabinomannan (LF-LAM) assay (when available).

For patients with advanced HIV disease, molecular tests and/or mycobacterial culture of otherfluids (e.g., blood, pleural fluid, ascitic fluid, cerebrospinal fluid, and bone-marrow aspirates)and histopathology (e.g., lymph node biopsies) may be helpful in diagnosis. [4]

Consider starting DR-TB treatment even in the absence of bacteriological confirmation if the clinical evidence and risk factors are strong (clinically diagnosed TB). **[4]**

Evaluate possible differential or **concomitant** diagnosis: non-tuberculosis mycobacterium (NTM), pneumocystis jirovecii pneumonia (PCP), pulmonary Kaposi's sarcoma, lymphoma, fungal pneumonia, herpes simplex or cytomegalovirus (CMV) pneumonitis and lymphoid interstitial pneumonitis (LIP in pediatric patients).

6.3 Drug-resistant TB and HIV co-treatment

- Antiretroviral therapy (ART) is recommended for all the patients with HIV and DR-TB, irrespective of CD4 cell counts, as early as possible, preferably within**2 and 8 weeks**after starting DR-TB treatment(if DR-TB treatment is tolerated well). [4]
- If the patient is already on ARVs, **request CD4 and viral Load** for early detection of ART failure. If the patient is failing need to be switched to second line without delays (as early as possible).
- Adverse events are frequent in PLHIV, some toxicities are common to both anti-TB treatment and ART [4] (see Table 7)
- Monitoring needs to be more intense to evaluate the response to therapy, adverse events and IRIS.

6.4 Potential overlapping toxicities in the treatment of HIV and drug-resistant TB

In general, HIV patients have a higher rate of adverse drug reactions (ADRs) to both TB and non-TB medications, and the risk increases with the degree of immuno-suppression. Identifying the cause of the adverse event is difficult, since many of the medications used to treat DR-TB and HIV have overlapping or additive toxicities. [4]

When possible, avoid the use of agents with shared toxicity profiles. However, if thebenefit of using drugs that have overlying toxicities outweighs the risk, it is recommended to increase the monitoring of adverse events rather than disallowing certain combinations. [4]

Table 7 : Potential overlapping and additive toxicities in the treatment of DR-TB and HIV [4], [12]

TOXICITY	ANTI-TB AGENT	ANTIRETROVIRAL AGENT	COMMENTS
Gastrointestinal (Nausea, vomiting, diarrhoea).	**Eto/Pto, PAS,** Cfz, H, Z, Bdq, Dlm and others.	NNRTIs-NVP, **PIs-RTV**, NRTIs-AZT, ddI & d4T	If diarrhea, consider opportunistic infections as the probable cause. Persistent vomiting may be due to other causes (e.g., hepatitis, lactic acidosis, meningitis, pregnancy etc).
Abdominal pain	**Eto/Pto, PAS,** Cfz, Lzd	All ARVs	Abdominal pain may be an early symptom of severe adverse drug reaction such as hepatitis, pancreatitis or lactic acidosis.
Dermatologic(Skin rash)	H, R, E, Z, FQ, Eto/Pto, Cfz	NNRTIs: **NVP & EFV**;	Do not re-challenge ABC as it can result in life threatening anaphylaxis. Do not re-

		NRTI: ABC	challenge with any agent that may have caused Stevens Johnson Syndrome.<br>Co-trimoxazole can also produce skin rash.
CNS toxicity and/or psychiatric symptoms (depression, psychosis)	**Cs**, H^h, FQ, Eto/Pto, Imipenem (Imp)/Meropenem (Mpn)	**Efavirenz (EFV)**	EFV CNS toxicity often resolve on their own after first 2-4 weeks of treatment. Rule out other causes.
Headache	Cs, Bdq	AZT, EFV	Rule out more serious causes of headache as meningitis, toxoplasma, etc.
Peripheral Neuropathy	H^h, Lzd, (less frequent: FQ, SLI, Cs, Eto/Pto, E)	Stavudine (d4T), Didanosine (ddI)	Patient receiving H^h, Cs and/or Lzd should receive prophylaxis with pyridoxine (B6).<br>Avoid use of d4t and ddi with Cs and Lzd.<br>If peripheral neuropathy is due to Lzd and is grade ≥ 2 stop Lzd and don't reintroduce.
Renal symptoms with hypokalemia	**SLI (Am, Km, Cm)**	**Tenofovir (TDF)**: Rare	Avoid concomitant use of TDF and SLI if possible. Even without the concomitant use of TDF, PLHIV have an increased risk of renal toxicity secondary to SLI. Frequent creatinine and electrolytes monitoring is recommended. Adjust medication doses if clearance <30 ml/min.
Hematological (Bone Marrow toxicity)	**Lzd**	**Zidovudine (AZT)**	Monitor full blood count monthly when using Lzd. All patients with Lzd should receive pyridoxine (Vit B6) 100 mg daily.<br>Consider other causes as cotrimoxazole, HIV infection, opportunistic infections.
Hepatotoxicity	**Z, H^h,** Eto/Pto, PAS, Bdq	**NVP, EFV,** all NRTIs, all PIs	When ALT/AST are elevated > 5 times stop both ART and anti TB agents and restart the TB medication first (see chapter on adverse event management).<br>Rule out other causes such as viral hepatitis (A, B, C and CMV).
Pancreatitis	**Lzd**	d4T, ddI	Avoid the use of these agents together.<br>If an agent caused pancreatitis suspend it permanently.<br>Consider other causes as gallstones or excessive alcohol use.
Lactic acidosis	**Lzd**	d4T, ddI, AZT	If an agent has caused high lactate or lactic acidosis stop permanently and replace it.
Optic Neuritis	**E, Lzd**	ddI	Suspend permanently the agent that caused optic neuritis and replace it.
Hypothyroidism	**Eto/Pto, PAS**	d4T	Monitor Thyroid Stimulating Hormone (TSH) and replace with levothyroxine when necessary (TSH >10 mIU/l)

Dysglycaemia	**Gfx, Eto/Pto**	PIs	Eto/Pto can make insulin control in diabetic patients more difficult (hypoglycemia and poor glucose regulation).<br>PIs can cause insulin resistance and hyperglycemia.
Arthralgia	**Z** (less frequent FQ and Bdq)	PIs	Arthralgia is very common with Z, also reported with FQ, Bdq and PIs.
QT Prolongation	Bdq, Dlm, Mxf, Gfx, Cfz (less frequently with Lfx)	ART has been associated with QT prolongation	Unknown data on additive effects of combining ART with second line anti-TB drugs for prolonging QTc. If the QTc is prolonged >500ms stop DR-TB QTc prolonging drugs but do not stop ART.

6.5 Drug-drug interactions in the treatment of HIV and drug-resistant TB

Bedaquiline interactions with ARVs (NNRTI and PIs):

Bedaquiline is metabolized by the CYP3A4. Many drugs can either induce or inhibit the CYP3A4, resulting in drug–drug interactions.Interaction with CYP3A4 inducers can lead to loss of efficacy of bedaquiline (due to lowblood concentration from increased metabolism of the drug), and conversely, interaction withstrong and moderate inhibitors of CYP3A4 can lead to higher exposure (higher blood levels)of bedaquiline. [4]

- **Efavirenz:** co-administration with Bdq may result in reduced Bdq exposure and loss of efficacy as EFV is a moderate inducer of CYP3A activity, hence this is **not recommended**
- **Nevirapine:** co-administrated with Bdq in HIV-infected patients has no clinically relevant effect, hence **can be co-administered**.
- **Lopinavir/ritonavir and other protease Inhibitors:** when co-administered with Bdq results in increased in Bdq plasma concentrations, hence should be **used with caution** and frequent monitoring of ECG and LFTs.

6.6 Use Bedaquiline and Delamanid with ART

Delamanid: has no interactions with ART and is preferable for PLHIV eligible for individualized DR-TB treatment regimen containing new drugs.

Bedaquilinecan be used in PLHIV taking the following into consideration:

- Avoid the concomitant use with Efavirenz [4]
- Use with caution with protease inhibitors. [4] If the patient is on second line ART and there is no access to integrase Inhibitors, PIs can be used with frequent monitoring of ECG and LFT.

Use of Bdq with ART:
<u>Patients not yet on ART:</u>

Initiate NVP-based regimen; irrespective of the CD4 count and within 2-8 weeks. When Bdq is completed substitute NVP with EVF.

Patients already on ART:

If the patient is already on ART with EFV, assess the viral load:

- *If the viral load is* ***undetectable***, the patient can be substituted from EFV to NVP during all period of therapy with Bdq. When Bdq is completed, can switch back to EFV.
- *If the viral load is* ***detectable***, the patient should be switch to second line ART regimen using integrase inhibitors (dolutegravir or raltegravir). If integrase inhibitors are not available, protease inhibitors (lopinavir/ritonavir) can be considered with caution (monitoring more frequently ECG and LFT).

If the patient is already on second line regimen with Protease Inhibitors (lopinavir/ritonavir), assess the viral load:

- *If the viral load is* ***undetectable*,** substitute lopinavir/ritonavir with dolutegravir or raltegravirduring all periods of therapy with Bdq. As soon as Bdq is completed can be switched back to lopinavir/ritonavir. If integrase inhibitors are not available continue with lopinavir/ritonavir with caution.
- *If the viral load is* ***detectable***, the patient should be switched to third line regimen(using integrase inhibitors as dolutegravir or raltegravir).

7. Hepatitis C - HCV (Adopted/Adapted from WHO guideline 2016).

7.1 Epidemiology:

The number of deaths per year due to HCV-related diseases continues to increase. According to estimates from the Global Burden of Disease study, the number of deaths due to hepatitis C was 333,000 in 1990, 499,000 in 2010 and 704,000 in 2013. Some countries are also experiencing a recent resurgence of HCV infection among young people who inject drugs (PWID) and HIV-infected men who have sex with men (MSM).

A more recent systematic review that excluded older studies estimated that worldwide 110 million persons are HCV-antibody positive and 80 million have chronic infection. A systematic review of studies from Africa showed a prevalence of HCV of 2.98%, with a higher prevalence observed in studies from West Africa and lower in studies from South-east Africa.

There are no reliable estimates of the global prevalence of HIV/HCV coinfection. One analysis indicates that 2.3 million persons may be co-infected globally, while an analysis from Africa estimated that 5.7% of persons with HIV were co-infected with HCV. It is worth noting that the prevalence of coinfection is generally lower in settings where the primary route of HIV transmission is not through injection drug use.

Groups at increased risk of infection with HCV are also at risk of infection with tuberculosis (TB), as TB is endemic in many countries where blood products are not screened routinely. TB is also the most common AIDS-defining illness and the leading cause of HIV-associated mortality.

Hepatitis B virus (HBV) and HCV coinfection is commonly found in HBV-endemic countries in Asia, sub-Saharan Africa and South America. Up to 25% of HCV infected persons may be co-infected with HBV in some areas.

Although there are no population-based estimates for this parameter, a reasonable assumption is that between 10% and 30% of persons with chronic HCV infection have stage F3 or F4 fibrosis.

7.2 Natural history of HCV infection:

HCV causes both acute and chronic hepatitis. Chronic infection with HCV is usually clinically silent, and is only very rarely associated with life-threatening disease. Spontaneous clearance of acute HCV infection occurs within six months of infection in 15–45% of infected individuals in the absence of treatment. Almost all the remaining 55–85% of persons will harbour HCV for the rest of their lives (if not treated) and are considered to have chronic HCV infection. Anti-HCV antibodies develop as part of acute infection and persist throughout life. In persons who have anti-HCV antibodies, a nucleic acid test (NAT) for HCV RNA, which detects the presence of the virus, is needed to confirm the diagnosis of chronic HCV infection.

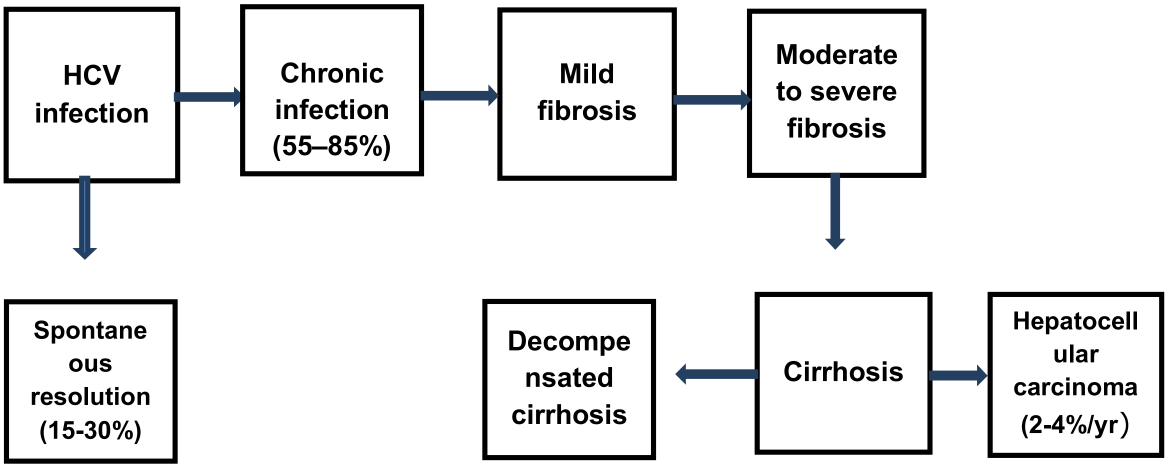

7.3 Screening for HCV infection:

Screening for HCV infection is done using HCV serological testing. If positive, a NAT for HCV RNA is needed to confirm chronic HCV infection. Several screening assays have been evaluated by WHO, and sensitivity, specificity, and positive and negative predictive values are available. It is important to consider the possibility of infection with other blood-borne viruses in persons infected with HCV, and to offer screening for HBV and HIV in addition to HCV. Screening for other infections, for example TB, is also indicated in some groups at risk, such as people living with HIV, prisoners and PWID (people who inject drugs).

7.4 Genotype testing:

In most countries, there is a mix of HCV genotypes among persons with chronic HCV infection. The 2016 Guidelines provide recommendations on the preferred and alternative DAA regimens by HCV genotype. Therefore, knowing a patient's genotype is still important for determining the most appropriate treatment regimen. Genotyping is usually carried out following sequencing of the 5'UTR (untranslated region) or of the NS5B region of the HCV genome. Genotype determination, however, is expensive and not available in all settings. Where genotype information is unavailable, pragmatic decision-making may be required, taking into account the common genotypes circulating in the affected population. However, this advice would only be practicable in countries such as Egypt or Mongolia, where almost all persons are infected with a single genotype.

7.5 Assessment of HCV treatment:

7.5.1 Existing recommendation from 2014:
All adults and children with chronic HCV infection, including people who inject drugs, should be assessed for antiviral treatment.

7.5.2 New recommendation:

Treatment with direct-acting antiviral agents**: it is recommended that DAA (direct-acting antiviral) regimens be used for the treatment of persons with hepatitis C infection rather than regimens with pegylated interferon and ribavirin.**

Subgroup considerations: **for patients with HCV genotype 3 infection with cirrhosis, and patients with genotypes 5 and 6 infection with and without cirrhosis, an interferon-based regimen – sofosbuvir/pegylated interferon/ribavirin – is still recommended as an alternative treatment option.**

Table 8: Summary of recommended preferred regimens with treatment durations*:

Persons without cirrhosis:

	Daclatasvir / Sofosbuvir	Ledipasvir / Sofosbuvir	Sofosbuvir / Ribavirin
Genotype 1	12 weeks	12 weeks[a]	
Genotype 2			12 weeks
Genotype 3	12 weeks		24 weeks
Genotype 4	12 weeks	12 weeks	
Genotype 5		12 weeks	
Genotype 6		12 weeks	

Persons with cirrhosis:

	Daclatasvir / Sofosbuvir	Daclatasvir / Sofosbuvir / Ribavirin	Ledipasvir / Sofosbuvir	Ledipasvir / Sofosbuvir / Ribavirin	Sofosbuvir / Ribavirin
Genotype 1	24 weeks	12 weeks	24 weeks	12 weeks[b]	
Genotype 2					16 weeks
Genotype 3		24 weeks			
Genotype 4	24 weeks	12 weeks	24 weeks	12 weeks[b]	
Genotype 5			24 weeks	12 weeks[b]	
Genotype 6			24 weeks	12 weeks[b]	

* Treatment durations are adapted from the 2015 guidelines of the American Association for the Study of Liver Diseases (AASLD) and European Association for the Study of the Liver (EASL).

a Treatment may be shortened to 8 weeks in treatment-naive persons without cirrhosis if their baseline HCV RNA level is below 6 million (6.8 log) IU/mL. The duration of treatment should be shortened with caution.

b If platelet count <75 x 10^3/µL, then 24 weeks' treatment with ribavirin should be given.

Table 9: DAAs presently available and accessible in Nigeria at this point:

Product	Presentation	Posology (Doses)[a]
Sofosbuvir/Daclastavir	Tablets containing 400 mg of sofosbuvir and 60 mg of daclatasvir.	One tablet once daily.
Sofosbuvir/Ledipasvir	Tablets containing 400 mg of	One tablet once daily.

	sofosbuvir and 90 mg of ledipasvir.	
Ribavirin	Capsules containing 200 mg of ribavirin	If body weight <75 kg – 2 capsules in the morning & 3 in the evening.<br>If body weight >75 kg – 3 capsules in the morning and 3 in the evening.

a. Dosages are adapted from the 2015 guidelines of the American Association for the Study of Liver Diseases (AASLD) and European Association for the Study of the Liver (EASL).

Existing recommendations (from the 2014 guidelines document):

Screening to identify persons with HCV infection: it is recommended that HCV serology testing be offered to individuals who are part of a population with high HCV prevalence or who have a history of HCV risk exposure/behaviour.

When to confirm the diagnosis of chronic HCV infection: it is suggested that nucleic acid testing (NAT) for the detection of HCV ribonucleic acid (RNA) be performed directly following a positive HCV serological test to establish the diagnosis of chronic HCV infection, in addition to NAT for HCV RNA as part of the assessment for starting treatment for HCV infection.

Screening for alcohol use and counselling to reduce moderate and high levels of alcohol intake: an alcohol intake assessment is recommended for all persons with HCV infection followed by the offer of a behavioural alcohol reduction intervention for persons with moderate-to-high alcohol intake.

Assessing degree of liver fibrosis and cirrhosis: in resource-limited settings, it is suggested that the aminotransferase/platelet ratio index (APRI) or FIB-4 (age in years, AST, ALT and platelets) test be used for the assessment of hepatic fibrosis rather than other noninvasive tests that require more resources such as elastography or FibroTest.
APRI & FIB-4 formulas:
APRI = [{AST (IU/L) / AST_ULN (IU/L) x 100} / platelet count (10^9/L).
FIB-4 = Age (yrs) x AST (IU/L) / platelet count (10^9/L) x [ALT (IU/L)$^{1/2}$].
ALT: alanine aminotransferase, AST: aspartate aminotransferase, IU: international unit, ULN: upper limit of normal.

Assessing for HCV treatment: all adults and children with chronic HCV infection, including people who inject drugs, should be assessed for antiviral treatment.

Treatment with pegylated interferon and ribavirin: pegylated interferon in combination with ribavirin is recommended for the treatment of chronic HCV infection rather than standard non-pegylated interferon with ribavirin.

7.6 Monitoring of treatment:

Table 10: Framework for the frequency of monitoring patients undergoing HCV therapy based on type of regimen:

Time	DAA alone			DAA+ribavirin			DAA+pegylated interferon+ribavirin			
	FBC, renal & liver functions	Adherence, side effects	HCV RNA	FBC, renal & liver functions	Adherence, side effects	HCV RNA	FBC, renal & liver functions	Thyroid function	Adherence, side effects	HCV RNA
**Baseline**	X		X	X		X	X	X		X
**Week 1**				X	X		X		X	
**Week 2**				X	X		X		X	
**Week 4**	X	X		X	X		X		X	
**Week 8**				X	X		X		X	
**Week 12**				X	X		X	X	X	
**Week 12 after end of treatment**			X	X		X	X	X		X
**Week 24 after end of treatment**										X

Table 11: Drug-Drug interactions between co-administered HCV & HIV treatment:

HIV ARVs	Daclatasvir	Ledipasvir/Sofosbuvir	Sofosbuvir	Ribavirin
**Nucleoside Reverse Transcriptase Inhibitors:**				
Abacavir (ABC)	♥	♥	♥	≠
Emtricitabine (FTC)	♥	♥	♥	≠
Lamivudine (3TC)	♥	♥	♥	≠
Tenofovir (TDF)	♥	≠	♥	≠
Zidovudine (AZT)	♥	♥	♥	⊗
**Entry / Integrase Inhibitors:**				

Dolutegravir (DTG)	♥	♥	♥	♥
**Non-nucleoside Reverse Transcriptase Inhibitors:**				
Efavirenz (EFV)	≠	≠	♥	♥
Nevirapine (NVP)	≠	♥	♥	♥
**Protease Inhibitors:**				
Lopinavir (LPV)	♥	♥	♥	♥
Ritonavir (r)	≠	♥	♥	♥

Source: University of Liverpool hepatitis drug interactions webpage.
♥ No clinically significant interaction expected.
≠ Potential interaction.
⊗ These drugs should not be co-administered.

Therapy with direct-acting antivirals (DAAs): contraindications/warnings:

Ledipasvir/sofosbuvir:
- Amiodarone co-administration
- P-glycoprotein (gp) inducers
- Renal failure (eGFR <30 mL/min/1.73 m^2)

Daclatasvir:
- Drugs inducing or inhibiting CYP3A

Sofosbuvir:
- Amiodarone co-administration (caution also with beta-blockers)
- Renal failure (eGFR <30 mL/min/1.73 m^2)

Ombitasvir/dasabuvir/paritaprevir/ritonavir or ombitasvir/dasabuvir/ritonavir:
- Child–Pugh Class B and C cirrhosis
- Drugs inducing or inhibiting CYP3A or CYP2C8
- Hypersensitivity to any component including ritonavir
- Untreated HIV-1 infection because ritonavir can lead to antiretroviral drug resistance.

7.7 Special Considerations for specific population:

7.7.1 Persons with TB/HCV co-infection:
People at increased risk of infection with HCV are also often at increased risk of infection with TB. Therefore, screening for active TB should be part of the clinical evaluation of patients being considered for HCV treatment. WHO recommends a four-symptom screening algorithm to rule out active TB in PLHIV (people living with HIV/AIDS). If the patient does not have any one of the following symptoms – current cough, fever, weight loss or night sweats – TB can be reasonably excluded; otherwise, the patient should undergo further investigations for TB or other diseases.
Most of the DAAs interact with metabolic pathways in the liver, which increases and/or decreases the drug level of DAAs when co-administered with antimicrobial medicines such as rifabutin, rifampin and rifapentine. Therefore, concurrent treatment of HCV infection and TB should be

avoided. Active TB should generally be treated before commencing therapyfor HCV. Furthermore, in persons with HCV infection being treated for TB, it is important to monitor liver function tests, as the risk of antimycobacterial induced hepatotoxicity is higher in patients with TB/HCV coinfection than in those with TB mono-infection, although the risk of severe hepatotoxicity is rare.

Concurrent treatment of HCV infection and multidrug-resistant TB is particularly complicated because of many DDIs between DAAs and second-line antimicrobials. There are limited data on the management of persons co-infected with HCV, HIV and TB, but such cases need sound clinical judgement in order to reduce the additive side-effects, pill burden and DDIs. Clinicians need to be aware of the risk of reactivation of TB if the person, particularly if HIV co-infected, receives interferon-based therapy, as interferon-based therapy could increase the incidence of active TB.

7.7.2 Persons with HIV/HCV co-infection:

Persons with HIV/HCV coinfection generally have more rapid progression of liver fibrosis, especially those with a CD4 cell count of <200 cells/mm^3. Furthermore, even among patients in whom ART leads to successful control of HIV infection (i.e. undetectable HIV viral load), the risk of hepatic decompensation among co-infected patients is higher than among patients with HCV mono-infection. For these reasons, all persons with HIV/HCV coinfection should be considered for HCV treatment.

Treating such patients in the past with interferon and ribavirin combination therapy was very difficult, as many patients had to discontinue treatment due to side-effects such as depression or weight loss as well as severe anaemia, thrombocytopenia and neutropenia. Furthermore, SVR rates in patients with coinfection were lower than among HCV-monoinfected patients.

Outcomes of HCV therapy with DAAs in persons with HIV coinfection are comparable to those with HCV mono-infection. Thus, DAA therapy has substantially simplified the treatment of persons with HIV and HCV coinfection. There are fewer DDIs between DAAs and ARV medicines, and SVR rates with DAA-based therapy among persons with HIV coinfection are higher than 95%, even for those with prior HCV treatment failure or advanced fibrosis. Therefore, there is no longer a need to consider HIV/HCV co-infected patients as a special, difficult-to treat patient population. The need to check for DDIs between HIV and HCV medications, however, needs to be emphasized.

It is advisable to first initiate treatment for HIV and achieve HIV suppression before starting HCV treatment, although there are some circumstances where it may make sense to treat HCV infection first and then initiate therapy for HIV. This could include persons with moderate-to-severe fibrosis at risk of rapid liver disease progression if the HIV infection is not associated with significant immunosuppression at the time of treatment. Also, in view of the short duration of HCV treatment, the risk of DDIs between HCV and HIV medicines and the increased risk of ART-related hepatotoxicity in the presence of HCV infection, treating HCV infection first can simplify subsequent ART depending on the regimen available locally. Persons co-infected with HIV are at higher risk of developing side-effects of HCV therapy, and should be monitored more closely. Before starting HCV therapy, careful consideration of DDIs is essential. Where DDIs are likely, ARV drug substitutions should be made before commencement of HCV therapy. It is particularly important to be aware of HIV infection when considering ritonavir based therapies (such as paritaprevir/ombitasvir/dasabuvir) in order to avoid single-drug treatment of HIV infection, which could lead to drug resistance to ARVs. Given that many countries will not have access to a wide range of HCV therapies and may have limited opportunities for re-treatment, it is critical that co-

infected patients be carefully assessed and any drug interactions that may either reduce efficacy or increase the risk of side-effects avoided.

Potential harmful effects of ARV drugs include their hepatotoxic effects. Several studies have shown that hepatotoxicity as a result of ART may be worsened in the presence of concomitant HCV infection. However, the highest rates of hepatotoxicity have been observed with ARV drugs that are no longer commonly used or recommended, including stavudine (d4T), didanosine (ddI), nevirapine (NVP) or full-dose ritonavir (600 mg twice a day). For most HIV/HCV-coinfected persons, including those with cirrhosis, the benefits of ART outweigh concerns regarding drug-induced liver injury.

Raised liver enzymes may be the result of ART-induced drug toxicity and/or opportunistic infections, making interpretation of liver enzyme elevations more problematic than for patients with HCV infection alone. ALT and AST should be monitored at 1 month after ART initiation and then every 3–6 months. A significant elevation of AST/ALT may prompt careful evaluation for other causes of liver function impairment (e.g. alcoholic hepatitis, hepatobiliary disease), and may require short-term interruption of the ART regimen or specific drug suspected of causing the elevation.

8. Treatment monitoring

Patients should be monitored closely for signs of treatment failure and adverse drug reactions (baseline and follow up examinations):

- Treatment can be monitored through clinical history, physical examination, psychosocial assessment and adherence, chest radiography, electrocardiography (ECG), audiometry, bacteriological test (smear and culture), laboratory monitoring (hematology-FBC, Creatinine, Potassium, LFT, TSH), baseline pregnancy test, hepatitis B, C and HIV test (if positive CD4 and VL every six months).
- Weight should be monitored monthly and drug dosages should be adjusted accordingly.
- For patient under individualized regimen, additional monitoring is required: serum albumin (Dlm), vision test charts, serum amylase/lipase and hematology-FBC (Lzd).

8.1 Monitoring progress of treatment

Patients should be monitored closely for signs of treatment failure. Monitoring response totreatment is done through regular clinical monitoring(history taking, physical examination), bacteriological monitoring and chest radiograph.

8.1.1 Clinical monitoring:

Patients should be asked about their TB symptoms and their weight should be recorded at every encounter. Children's height and weight should be measured monthly to ensure that they are growing normally. For adults, weight should be recorded monthly (height is only recorded at the start of treatment).
Persistent fever, weight loss or recurrence of any of the classic symptoms of TB should prompt investigation of treatment failure or untreated co-morbidities (bacterial pneumonia is very frequent in patients with damaged lung due to TB).

8.1.2 Bacteriological monitoring:

Sputum smear and culture examination must be performed monthly throughout the entire treatment duration. (Molecular tests are not recommended for treatment monitoring).

- Culture conversion is not equivalent to cure. A certain proportion of patients may initially convert and later revert to positive sputum cultures.
- The most important evidence of improvement is conversion of the culture to negative. While smear is useful because of its much shorter turnaround time, the culture is much more sensitive to detect ongoing active disease and/or treatment failure.
- For patients who remain smear or culture positive after more than four months of treatment or who are suspected to have treatment failure, SL-LPA and phenotypic DST should be repeated.
- In addition to pointing to treatment failure, persistently positive sputum smears may indicate colonization of damaged lungs by mycobacteria other than TB. This may be confirmed by two mycobacterial culture results. In such cases, DR-TB should continue being adequately treated and treatment for NTM added as well. [4]

8.1.3 Radiological monitoring:

- The chest radiograph may appear unchanged in the first few months of treatment or only show a slight improvement, especially in patients with chronic pulmonary lesions.
- Chest radiographs should be taken at baseline, every six months and at the end of the treatment to document progress and to use for comparison if the patient's clinical condition changes.
- Chest radiography is also done when a surgical intervention is being considered, or whenever the patient's clinical situation worsens. [4]

8.2 Monitoring Adverse Events

All Adverse Events (AEs) including adverse drug reactions (ADR) should be monitored in an active, systematic, and timely manner for early diagnosis and prompt management (important component of aDSM).

The assessment of patients under DR-TB treatment should include clinical examination, laboratory exams and complementary tests such as ECG, audiometry, and vision tests.

Psychosocial consultation and patient education are also crucial for detection of AEs and adherence to the treatment.

8.2.1 Clinical monitoring of adverse drug reactions (ADRs)

Patients should be routinely screened for ADRs at least weekly during the first month of intensive phase, and monthly for the entire duration of the treatment.

DOT providers should be trained to screen patients regularly for symptoms of common ADRs: rash, gastrointestinal disturbances (e.g., nausea, vomiting, and diarrhea), psychiatric symptoms (e.g., depression, anxiety, suicidal ideation and behavioral changes), jaundice, ototoxicity, peripheral neuropathy and symptoms of electrolyte wasting (e.g., muscle cramping, palpitations).

DOT providers should also be trained in simple adverse effect management and when to refer patients to a nurse or doctor.

8.2.2 Educational, psychological and social consultation

Patient education and psychosocial consultation should be done at baselineby personnel trained in health education, psychological and social issues relevant to DR-TB management and adherence. Patient education should include clear information about adverse drug reactions (e.g., symptoms, importance of prompt consultation, basic management).

Should be repeated during the treatment monthly at every consultation. The patient should be referred to a social worker, psychologist or psychiatrist when indicated.

8.2.3 Laboratory monitoring of AEs

Blood should be collected for monthly monitoring full blood count and biochemistry.

Test should include:

- **Full blood count:** if on linezolid, monitor weekly during first month, then monthly or as needed based on symptoms (there is little clinical experience with prolonged use of linezolid). For HIV-infected patients on zidovudine, monitor monthly initially and then as needed based on symptoms.
- **Creatinine including calculation of clearance (CrCl) and electrolytes (potassium):**Monitoring monthly while receiving an injectable agent. Every two weeks in HIV infected patients, diabetics and other high-risk patients in the beginning and later if needed. If hypokalemia is diagnosed, monitor also calcium and magnesium. Monitor electrolytes more frequently in patients with ECG abnormalities (prolonged QTcFr).
- **Liver function test (ALT/AST):**Periodic monitoring (every 1-3 months) in patients receiving pyrazinamide for extended periods or for patients at risk for, or with symptoms of hepatitis. For HIV-infected patients monthly monitoring is recommended. For patients on Bdq, monitor monthly. For patients with viral hepatitis, monitor every one to two weeks for the first month and then every 2-4 weeks.
- **Thyroid Stimulating Hormone (TSH):**Monitorat baseline and every six months if on ethionamide, prothionamide, or PAS. If levothyroxine replacement is required, monitor every 30-45 days to adjust the dosage.
- **Fasting blood sugar:** At baseline and when required. Monitor frequently in patients with diabetes.
- **Serum Albumin:** If patient on Dlm, at baseline and every two months.
- **Serum amylase and lipase:**Special attention to patients receiving Bdq, lzd, D4T, ddI and based on risk factors. Perform at baseline and when required.
- Other baseline investigations: **HIV test** (if positive, CD4 and Viral Load), **pregnancy test, hepatitis B and C.**

8.2.4 ECG Monitoring

Bedaquiline, Delamanid, Moxifloxacin and Clofazimine are potentially cardiotoxic, with toxicity manifesting as prolongation of the QTcFr interval (that increase the risk of arrhythmias).

The ECG monitoring is mandatory for patients on shorter regimen and Bdq/Dlm.

The ECG should be performed at baseline, every two weeks in the first month and then monthlt till the end of treatment.

More frequent monitoring is required in patient with low albumin (<3,4 g/dl), low electrolytes, hypothyroidism, heart conditions or clinical symptoms of cardiotoxicity (tachycardia, syncope, palpitations, weakness or dizziness).

The QT interval is influenced by heart rate: shortens at faster heart rates, and lengthens at slower heart rates. It need to be corrected by a formula (QTc) which estimates the QT interval at a heart rate of 60 bpm. This allows comparison of QT values over time at different heart rates.

How to measure QT Interval and calculate the QTc Fr:
The QT interval is that portion of the ECG that begins at the start of the QRS complex and stops at the end of T wave. It measures the time required for the ventricle to depolarize and repolarize. It is measured in seconds (s).

1) From the 12-lead ECG printout, choose **Lead II, V5 or V6** as they usually best show the end of the T wave.

Figure 6: QT interval

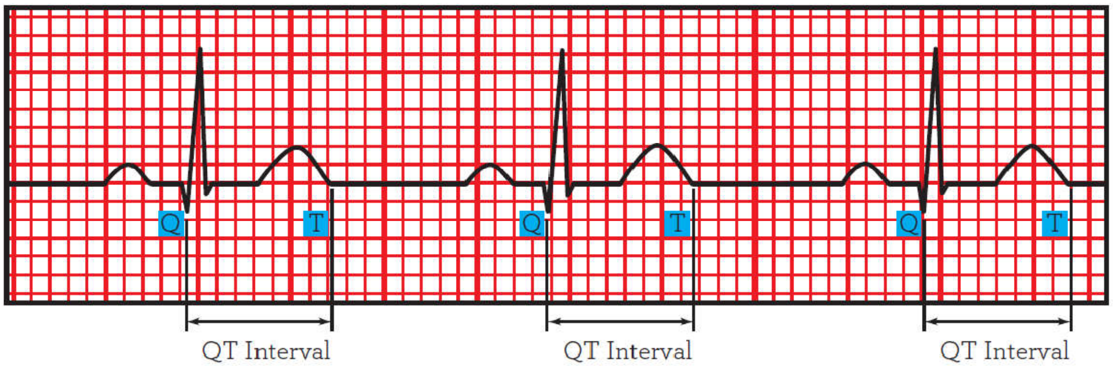

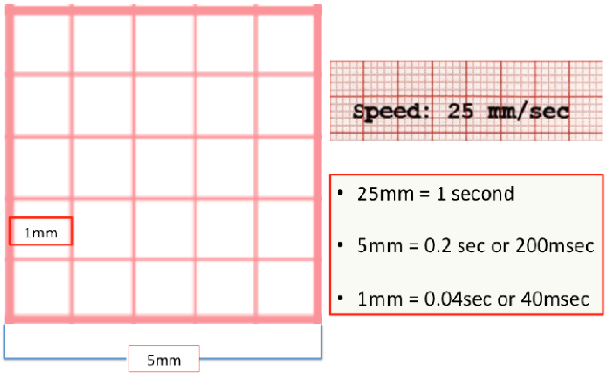

Each small square = 1 mm = 0.04 seconds when the speed of scrolling is 25 m/sec.

QT (in sec) = number of small squares x 0.04

2) Measure the QT interval (in seconds) from the beginning of the QRS complex to the end of the T wave. This is the uncorrected QT. Measure at 3 to 5 successive beats and take the maximum interval.

- Make an imaginary line on Q and on T on one heartbeat on the selected lead
- Count the number of small squares between Q and T: e.g.8 small squares in the example
- Multiply the number of squares by the unit time per square (0.04 sec): 8 small squares x 0.04 sec = 0.32 seconds

3) Measure the RR interval

- Make an imaginary line on two consecutive R waves
- Count the number of small squares between the two Rs: e.g. 20 small squares in the above example.

- Multiply the number of small squares by the unit time per square (0.04): 20 small squares 0.04 sec = 0.80 seconds. The RR interval is 0.80 sec. [24]

4) Calculate the corrected QT interval (QTc)

- The corrected QT interval (QTc) estimates the QT at a heart rate of 60 bpm.The QTc will be calculated using the **Framingham's** formula:

$$\mathbf{QTc\ Fra = QT + 0.154\ (1\text{-}RR)}$$

Where:
QTcFra= the corrected QT interval
QT = the time between the start of the QRS complex and the end of the T wave (sec)
RR = The RR interval is the time of the ECG from one R wave to another (RR cycle length) (sec)
Then multiply the result by 1000 to have the QTcFra in milliseconds
QTcFra (sec) x 1000 = QTcFra (ms).

U waves: *Small U waves separated from the T wave should not be included in the QT measurement, but big U waves (> 1 mm) superposed with the T wave should be included in the QT measurement.*
Hypokalaemia causes apparent QT prolongation in the limb leads (due to T-U fusion) with prominent U waves in the precordial leads. [24]

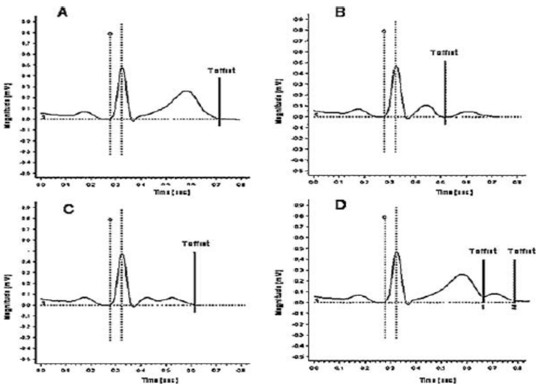

8.2.5 Audiometry

Audiometric examination should be performed at baseline and at least monthly while the patient is receiving the second-line injectable. In addition to physical examination of the ears with an otoscope, an audiogram is charted for each audiometric measurement, denoting the hearing intensity threshold (in decibels-dB, shown on the vertical axis) for each ear at various frequencies (in Hertz-Hz, shown on the horizontal axis). The threshold on the right ear is usually plotted as an O whereas that for the left ear is plotted as an X. Normal hearing threshold ranges from *0dB* to *25dB* hearing level. [23]

Figure 7: Example of an audiogram showing severe bilateral high frequency hearing loss

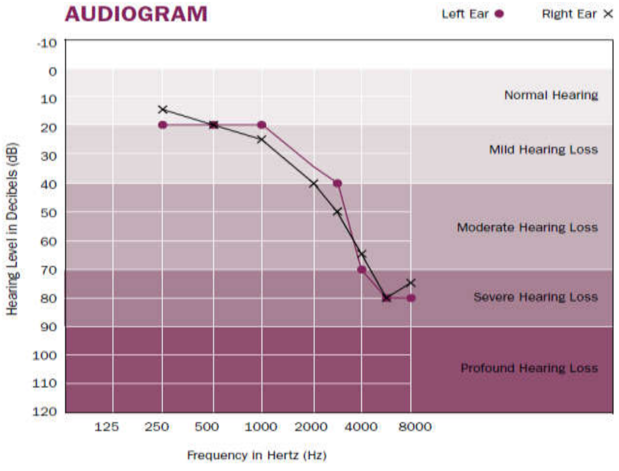

Source: Auditory neuroscience: making sense of sound. (http://auditoryneuroscience.com/acoustics/clinical_audiograms, accessed 30 June 2013)

8.2.6 Vision test charts

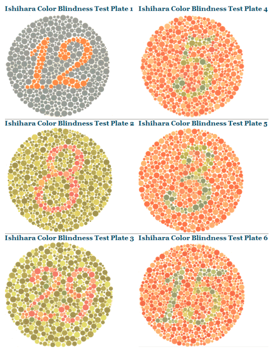

The patients on long-term ethambutol or linezolid perform a visual acuity test with Snellen's chart and color vision test with Ishihara's chart) at baseline (as a small percentage of the population hascolor blindness). Repeat the test if any change in acuity or color vision is suspected.[4]

8.2.7 Monitoring schedule for shorter and individualized regimens

Table8: Treatment monitoring schedule for shorter regimen

***Prompt action on abnormal clinical or laboratory findings is mandatory** *****Circle any parameter carried out**												
**Patient Name:** **Age:**			**DR-TB No:** **Sex:**									
**Month/Year**												
**Examination**	**Baseline**	**1**	**2**	**3**	**4**	**5**	**6**	**7**	**8**	**9**	**10**	**11**
**Clinical symptoms & exam**	X	X	X	X	X	X	X	X	X	X	X	X
**Toxicity symptoms & signs**	X	X	X	X	X	X	X	X	X	X	X	X
**Educational & psychosocial**	X	X	X	X	X	X	X	X	X	X	X	X
**Weight**	X	X	X	X	X	X	X	X	X	X	X	X
**Height &BMI (wt/ht^2)**	X											
**Xpert MTB/RIF**	X											
**SL LPA**	X				Repeat if smear and/or culture positive or presumption of failure							

**Sputum AFB**	X	X	X	X	X	X	X	X	X	X	X	X
**Specimen culture**	X	X	X	X	X	X	X	X	X	X	X	X
**Phenotypic DST**	X				Repeat if smear and/or culture positive or presumption of failure							
**X-Ray**	X						X					X
**ECG[6]**	X & week 2	X	X	X	X	X	X	X	X	X	X	X
**Haematology (FBC, diff)[1]**	X											
**Creatinine and potassium[2]**	X	X	X	X	X	X	X					
**LFTs (ALT/AST)**	X			X			X			X		
**TSH[3]**	X						X					
**Fasting blood sugar**	X											
**Vision test chards[4]**	X											
**Audiometry[2]**	X	X	X	X	X	X	X					
**HIV test[5]**	X											
**Hepatitis B, C**	X											
**Pregnancy test[6]**	X											
**HIV positive patients**												
**CD4 count**	X						X					
**Viral load**	X						X					

1. Repeat FBC as necessary if HIV-infected (especial care in patient with AZT)
2. Creatinine, potassium and audiometry should be request monthly while on injectable
3. TSH:If replacement with levotiroxine is required, repeat TSH every 30-45 days to adjust the dosage.
4. For patient on long term Ethambutol request vision test.Repeat if any change/complain in acuity or color vision
5. HIV test can be repeated if indicated (consider repeat every 6 months).
6. Pregnancy test: at baseline, then offer use of effective contraceptives (Depo-Provera or Intra uterine device-IUD)

Table 9: Treatment Monitoring Schedule for individualized regimen

Prompt action on abnormal clinical or laboratory findings is mandatory

*****Circle any parameter carried out***

Patient Name: **DR-TB No:**
Age: **Sex:**

**Month/Year**																					
**Examination**	**Baseline**	**1**	**2**	**3**	**4**	**5**	**6**	**7**	**8**	**9**	**10**	**11**	**12**	**13**	**14**	**15**	**16**	**17**	**18**	**19**	**20**
**Clinical** symptoms & exam	X	X	X	X	X	X	X	X	X	X	X	X	X	X	X	X	X	X	X	X	X
**Toxicity** symptoms & signs		X	X	X	X	X	X	X	X	X	X	X	X	X	X	X	X	X	X	X	
**Educational & psychosocial**	X	X	X	X	X	X	X	X	X	X	X	X	X	X	X	X	X	X	X	X	X

**Weight**	**X**	**X**	**X**	**X**	**X**	**X**	**X**	**X**	**X**	**X**	**X**	**X**	**X**	**X**	**X**	**X**	**X**	**X**	**X**	**X**	**X**
**Height/BMI (wt/ht^2)**	**X**																				
**Xpert MTB/RIF**	**X**																				
**SL LPA**	**X**					Repeat if smear and/or culture positive or presumption of failure															
**Sputum AFB**	**X**	**X**	**X**	**X**	**X**	**X**	**X**	**X**	**X**	**X**	**X**	**X**	**X**	**X**	**X**	**X**	**X**	**X**	**X**	**X**	**X**
**Specimen culture**	**X**	**X**	**X**	**X**	**X**	**X**	**X**		**X**		**X**		**X**		**X**		**X**		**X**		**X**
**Phenotypic DST**	**X**					Repeat if smear and/or culture positive or presumption of failure															
**X-Ray**	**X**						**X**						**X**						**X**		
**Haematology (FBC, diff)[1]**	**X&week 2**	**X**	**X**	**X**	**X**	**X**	**X**	**X**	**X**	**X**	**X**	**X**	**X**	**X**	**X**	**X**	**X**	**X**	**X**	**X**	**X**
**Creatinine and potassium[2]**	**X**	**X**	**X**	**X**	**X**	**X**	**X**														
**LFTs (ALT/AST)**	**X**	**X**	**X**	**X**	**X**	**X**	**X**		**X**		**X**		**X**		**X**		**X**		**X**		**X**
**TSH[3]**	**X**						**X**						**X**						**X**		
**Fasting blood sugar**	**X**																				
**Serum Albumin[4]**	**X**																				
**Serum amylase/Lypase[5]**	**X**																				
**ECG[6]**	**X & week 2**	**X**	**X**	**X**	**X**	**X**	**X**	**X**	**X**	**X**	**X**	**X**	**X**	**X**	**X**	**X**	**X**	**X**	**X**	**X**	**X**
**Vision test chards[7]**	**X**	**X**	**X**	**X**	**X**	**X**	**X**	**X**	**X**	**X**	**X**	**X**	**X**	**X**	**X**	**X**	**X**	**X**	**X**	**X**	**X**
**Audiometry[2]**	**X**	**X**	**X**	**X**	**X**	**X**	**X**														
**HIV test[8]**	**X**																				
**Hepatitis B, C**	**X**																				
**Pregnancy test[9]**	**X**																				
**HIV positive patients**																					
**CD4 count**	**X**						**X**						**X**						**X**		
**Viral load**	**X**						**X**						**X**						**X**		

1. **Repeat second line LPA and culture/phenotypic DST for patients who remain culture positive after more than four months of treatment.**
2. If on Linezolid, FBC at baseline, week 2 and then monthly
3. Creatinine, potassium and audiometry should be requested monthly while on injectable.
   Continue monthly creatinine monitoring for patients at more risk of renal failure (HIV positive patients, diabeteics patients receiving other nephrotoxic drugs).
   If any ECG abnormality check potassium (and Mg-Ca if available).
4. TSH:If on ethionamide, prothionamide, or PAS. If replacement with levothyroxine is required, repeat TSH every 30-45 days to adjust the dosage.
5. Serum albumin: If patient is on Dlm request serum Albumin at baseline: if low (<3,4 g/dl) monitor more frequent albumin and ECG.If serumalbumin is <2.8g/dl, Dlm is contraindicated.
6. If patient on Bdq and Lzd; request amylase and lipase at baseline. Then assess when clinically indicated
7. **ECG is mandatory for patients on Bdq, Dlm, Mfx and Cfz** at baseline, week 2 and then continue monthly ECG in patients receiving more than one QT prolongation drug (Bdq, Dlm, Mxf, Lfx,Cfz). More frequent monitoring is required in patient with low albumin (<3,4 g/dl), low electrolytes, hypothyroidism or heart conditions.

8. If patient on Linezolid, perform monthly visual acuity test (Snellen's chart) and color vision test (Ishihara's chart).
9. HIV test can be repeated if indicated(consider every6 months).
10. Pregnancy test: at baseline, then offer effective contraceptives(barrier contraception or intra uterine device-IUD).

9 Management of Adverse Events

The prompt evaluation, diagnosis and treatment of adverse events (AEs) is an extremely important component of aDSM, even if the AE is not particularly dangerous. [4]

Before starting treatment, the patient should be counseled in detail about the potential adverse events of the prescribedregimen, and when to notify a healthcare provider. [4]

Preventive treatment should be applied where necessary, e.g. pyridoxine (vitamin B6) 50 mg for every 250 mg of cycloserine or at preventive dose of 50-100 mg /day (up to 150 mg) for patients receiving Isoniazid or Linezolid (to minimize peripheral neuropathy, neurological adverse event and myelosuppression).

Psychosocial support is an important component of the management of adverse events. DOT providers should educate patients about adverse events and encourage them to continue treatment. Patient support groups can provide psychosocial support to patients. [4]

Any relevant clinical event (AEs or ADR) and any additional diagnostic testing required and/or therapy must be recorded. All serious adverse event (SAEs) should be notified within 24 hours (refer to aDSM chapter).

Management of AEs should take the safety of the patient and treatment need into consideration. For minor AEs, reassurance to enhance adherence is needed. For AEs that need additional evaluation and/or medical treatment, a treatment decision structure (consultation back-up for DOT provider), additional tests and ancillary medicines should be available and accessible, free of charge. [20]

If the drug(s) that are thought to cause the AE need to be removed from the regimen, replacements might be required, especially in the intensive phase when the bacillary load is high. Replacement of drugs should take the clinical condition and bacteriological status of patients into account. Ensure that the regimen contains at least four medicines with known effective drugs [20]. Any decision must be made on the basis of careful patient review (consult national consilium of DR-TB for guidance).

8.1 Severity grading scale of adverse events and main laboratory parameters

All SAEs and AEs should be graded for severity according to the Severity Grading Scale (Grades 1-4). For those AEs not described in the Severity Grading Scale, the general definition of clinical severity should apply.

Table 10: Severity grading scale of adverse events [18], [19]

**Grade 1 MILD**	**Grade 2 MODERATE**	**Grade 3 SEVERE**	**Grade 4 LIFE-THREATENING**
Transient or mild discomfort (<48 hours) without limitation of normal daily activities*. No medical intervention or corrective treatment required	Mild to moderate limitation of normal daily activities*. Minimal medical intervention or corrective treatment required	Marked limitation of normal daily activities* Medical intervention, therapy, stop or reduction of the offending drug is required. Possible hospitalization	Severe limitation of normal daily activities*. Medical intervention and corrective treatment required almost always in a hospital setting.

*The term 'activity' covers basic self-care functions such as bathing, dressing, transfer/movement, feeding; but also, usual social and functional activities or adaptive tasks and desirable activities, such as going to work, shopping, cooking, use of transportation, pursuing a hobby, etc.

Table 11: Severity grading scale of main laboratory parameters [18], [19]

	Hb (g/dl)	Platelets (/mm^3)	Neutrophils (/mm^3)	AST (UI/l)	ALT (UI/l)	Creat. (µmol/l)	K+ (mEq/l or mmol/l)
Normal values	>12	>150,000	>1,500	*	*	*	3.5-5.0
Grade 1	10-11.9	100,000-149,999	1,000-1,500	1.5 < 2.5 x ULN	1.5-<2.5 x ULN	1.1 – 1.5 x ULN	3.2-3.4
Grade 2	8-9.9	50,000-99,999	750-999	2.6-5.0 x ULN	2.6-5.0 x ULN	1.6 – 3 x ULN	2.8-3.1
Grade 3	6-8	20,000-49,999	500-749	5.1-10 x ULN	5.1-10 x ULN	3-6 x ULN	2.5-2.7
Grade 4	<6	<20,000	<500	>10 X ULN	>10 x ULN	> 6 x ULN	<2.5

*Normal values vary fromlaboratory to laboratory and might be slightly different in men, women and children. (Check normal parameters for your laboratory)
ULN= upper limit of normal

8.2 Clinical Management of adverse events

Tables 12: Management of Adverse Events [4],[12], [18], [19], [20], [21], [23], [24]

**Prolonged QT interval**<br>**Possible anti-TB drug causes: Bdq, Dlm, Mxf, Cfz.**

Normal Values	Grade 1<br>Mild	Grade 2<br>Moderate	Grade 3<br>Severe	Grade 4<br>Potentially Life-Threatening
**Male:**<br>**≤430**<br><br>**Female:**<br>**≤450**	**Borderline:**<br>Male:<br>430-450<br>Female:<br>450-470	**Prolonged:**<br><br>Male:<br>>450-<500ms<br><br>Female:<br>> 470-<500 ms	**Pathological:**<br>Male and Female:<br>≥ 500 ms<br>***or***<br>≥ 60 ms above baseline	**Life-threatening consequences:**<br>QTcFr ≥ 500 or >60 ms change from baseline and torsade de pointes or polymorphic ventricular tachycardia or signs/symptoms of serious arrhythmia
**Action**	Monitor monthy	Monitor more closely; at least weekly ECG until QTcFr has returned to Grade 1 or less. Check electrolytes and replete as necessary	Stop the QT prolonging drugs. Check and replete electrolytes as necessary.	Stop the QT prolonging drugs. Hospitalize and replete electrolytes as necessary.

Suggestions and precautions

- Close follow-up of patients at high risk who take several medicines that prolong the QT.
- Follow-up of potassium in high risk patients.
- Be careful in case of diarrhea, vomiting, use of loop diuretics, alcohol.
- Stop the medicine if QTc persists over 500ms even if the patient is asymptomatic.
- Think of arrhythmia when the patient suffers vertigo, syncope, palpitations.

If QTcF is prolonged ≥ 500 ms *(two repeated ECG):*

- Stop all QT prolonging drugs immediately. ART is usually not stopped unless the patient is severely unstable
- Hospitalize (consider continuous ECG monitoring).
- Check electrolytes and manage accordingly. (If low potassium: urgent management with replacement and frequent monitoring). Give magnesium sulphate supplements (orally or IV).
- Check TSH and treat any hypothyroidism found.
- Check albumin if on Delamanid.

Once stable (QTcFr< 450 and normal electrolytes), critical prolonging QT drugs can be added back:

- If the patient was on Mfx consider using Lfx instead.
- If the patient was on Cfz consider suspending it permanently (if not critical to the regimen).
- If the patient is on Bdq (or Dlm), and is critical to the regimen, add it back while suspending all other QT prolonging drugs (with the exception of stopping ART, which should not normally be

suspended in the management of QT prolongation).

**Hypokalemia**<br><br>**Possible anti-TBdrug causes: Am,Km,Cm, S**				
**Normal value**	**Grade 1**<br>**Mild**	**Grade 2**<br>**Moderate**	**Grade 3**<br>**Severe**	**Grade 4**<br>**Potentially Life-Threatening**
3.5-5.0 (mmol/l)	3.4-3.2	3.1-2.8	2.7-2.5	<2.5
**Action**	Continue injectable. Start oral potassium slow K* 600 mg = 8 mEq: 1 tab twice daily.<br><br>**Monitor K monthly**	Continue injectable. Start oral potassium slow K* 600 mg = 8 mEq: 2 tab twice daily<br><br>Oral Magnesium gluconate: 1000 mg twice daily.<br><br>**Monitor K every 2 weeks** and adjust the dose accordingly	Continue injectable. Start oral potassium: Slow K* 600 mg = 8 mEq: 2 tab thrice daily<br><br>Oral Magnesium gluconate: 1000 mg twice daily<br><br>**Monitor K every 1-2 days** and adjust dose accordingly	Stop injectable temporarily. Hospitalization**.** Start IV potassium in addition to oral. Replace magnesium and other electrolytes<br>**Monitor K 1 hour after replacement and repeat till K is >2.8 mmol/l**

* The formulations of oral potassium chloride varies by manufacturers and countries. Slow-release versions are common in low resources settings.One slow K 600 mg tab contains 8 mEq of potassium. **Adjust the number of pills according to the formulation available.**

Oral potassium and magnesium should be administered either two hours before or four to six hours after fluoroquinolones as they can interfere with fluoroquinolone absorption**.**

Replacing serum electrolytes:

- Replacement of 40 mEq of potassium increase 1 mEq/l the potassium.
- Oral replacement:The replacement varies 40 mEq to 80 mEq day. Usually patients don't tolerate more than 6-tab slow k (diarrhea, nausea). Doses should be divided to two or three times a day (no more than 20 mEq per dose)
- Hypokalemia may be refractory if concurrent hypomagnesemia is not also corrected.
- If unable to check serum magnesium, give empiric replacement therapy in all patients with hypokalemia: oral magnesium gluconate 1000mg twice daily.
- In refractory cases, can be given spironolactone 25 mg/day or Amiloride 5-10 mg/day orally (decreased potassium and magnesium excretion).

FOR HOSPITALIZED PATIENTS:
If severe hypokalemia (K ≤ 2.5 mmol/l or symptomatic hypokalemia): give Intravenous potassium concurrently with oral potassium replacement.
Dosing: 10-15 mEq /h IV and 80 mEq orally every six to eight hours.Recheck serum potassium 1 hour after infusion. Repeat IV replacement every 6 to 8 hours until serum potassium is ≥ 2.8 mmol/l.
The normal preparation of potassium chloride infusion is 40mEq in200mlof normal saline over 2-4 hrs.Do not exceed an infusion rate of 20 mEq/hr(100ml/hr).

Magnesium replacement:<br>Dosing: 2000 mg/day. If Mg can be measured and is less than 1.0, increase the dose up to 3000 mg-6000 mg (Mg doses greater than 2000 mg are usually given IV). Magnesium IV:<br>The normal preparation is magnesium sulfate2g in100ml or4gin250ml of5 % dextrose or normal saline. Do not exceed an infusion rate of 150mg/min (2gin100ml administered over one to two hours,4gin 250mladministered over two to four hours). Repeat until serum K is > 2.8mmol/l.<br><br>Other considerations:<br>• Check ECG in patients with significant serum electrolyte disturbances.<br>• Drugs that prolong the QT interval should be discontinued in-patients with evidence of QTc interval prolongation.<br>• Electrolyte abnormalities are reversible upon discontinuation of the injectable. Even after suspending the injectable, it may take weeks or months for this syndrome to disappear, so electrolyte replacement therapy should continue for several months after completion of the injectable phase of DR-TBtreatment (adjusting doses according to laboratory results).

**Nephrotoxicity**<br>**Possible anti-TBdrug causes: Am,Km,Cm, S**				
	**Grade 1**<br>**Mild**	**Grade 2**<br>**Moderate**	**Grade 3**<br>**Severe**	**Grade 4**<br>**Life-threatening**
Creatinine	1.1- 1.5 x ULN	1.6-3.0 x ULN	3.1-6 x ULN	> 6 x ULN or dialysis required
Creatinine clearance*<br>Normal value<br>Male: 97-137 ml/min<br>Female: 88-128 ml/min<br>CrCl grading [22]	>90ml/min	60-89ml/min	30-59ml/min	15-29ml/min<br><br>Note: < 15 ml/min is grade 5 and requires dialysis.
Action	Continue monitoring	Reduce injectable to 3 times a week.	Reduce injectable to 2 times a week.	**Stop injectable.** Monitor creatinine and electrolytes weekly until creatinine returns to normal.<br>Adjust the other drugs dosages
**Suspend the injectable permanently if the nephrotoxicity recurs despite intermittent dosing, and add additional anti-TB drugs to reinforce the regimen as Linezolid, Bedaquiline or Delamanid**(no dose adjustment is required for Lzd, Bdq or Dlm, use with caution and close monitoring of ADR)				

Consider other causes of renal insufficiency (pre-renal, intrinsic renal and post-renal).

***Creatinine clearance formula:**

$$\frac{\textbf{Weight (Kg) x (140 – Age) x (constant)}}{\textbf{Serum creatinine µmol/l}}$$

Constant: 1.23 for men and 1.04 for woman

If creatinine is reported in mg/dl multiply by 88.4 to convert to µmol/l

When **Cr Cl < 30 ml/min**, stop the injectable and monitor creatinine and electrolytes weekly until returns to normal. **Adjust all the TB medications according to Table 18in Annex B.**

Then reintroduce the injectable at a dose of 12-15 mg/kg/per dose 2 or 3 times per week, monitoring creatinine weekly.If the creatinine continues to rise suspend the injectable permanently and add additional anti-TB drug (Bdq, Dlm or Lzd).

**Hearing loss**<br>**(ANRS scale)**<br>**Possible anti-TB drug causes: Am,Km,Cm, S**				
**AUDIOMETRY TEST:**<br>Exclude causes of conductive hearing loss: ear wax, otitis media, tympanic perforation etc.<br>*Attain a baseline record of the hearing status of a person prior to treatment initiation.*<br>*Perform a monthly pure tone audiogram that includes speech frequencies (500-4000 Hz) and higher frequencies up to 8000 Hz.*<br>***Calculate the average hearing loss (AHL) for each ear:***<br>*sum the loss in dB at each frequencies of 500-1000-2000-4000 Hz (if a frequency is not perceived consider a loss of 120 dB) and divide by 4. You have the average of the best ear and the bad ear.*<br>***Then calculate the Weighted Average Hearing Loss (WAHL) for both ears:***<br>*WAHL= (Average of the best ear multiply by 7) +(Average of the worst ear multiplied by 3). Divide the total by 10.*<br>*WHO grading of hearing impairment:*				
**Normal Values**	**Grade 1**<br>**Mild**	**Grade 2**<br>**Moderate**	**Grade 3**<br>**Severe**	**Grade 4**<br>**Profound**
**0-25 dB**	**26-40 dB**<br>Speech perceived if voice is normal, difficulties arise if voice is low-pitched or distant from the subject. Most of the daily life noises are perceived	**41-60 dB**<br>Speech is perceived if the voice is loud. The subject understands better what is being said if he can see his/her interlocutor.	**61-80 dB**<br>Speech is perceived if the voice is loud and close to the ear. Loud noises are perceived.	**>80dB**<br>Speech is not perceived at all. Only very loud noises are perceived.
*** Grades 2, 3 and 4 are classified as disabling hearing impairment (for children, it starts at 31 dB)**<br>**Action:**<br>**Confirm results on repeated test in the same visit before any change in the treatment.**<br>Before reducing or stopping a drug: Think carefully, consult other experts.<br>If ototoxicity is detected early, it may be possible to stop the injectable preventing progression of the hearing loss.<br>**If vestibular disorder (vertigo, dizziness, imbalance, disequilibrium, nausea and vision problems) injectable should be stopped**.				
**Patient with no hearing loss at baseline:**				
**Injectable**	Continue injectable but consider reducing the frequency to 3 times per week	Stop the injectable and replace by new drugs Bdq, Dlm or Lzd.<br>Refer to audiologist.	Stop the injectable and replace by new drugs Bdq, Dlm or Lzd.	Stop injectable and replace by new drugs Bdq, Dlm or Lzd.<br>Refer to

	and perform more frequent audiometry.		Refer to audiologist.	audiologist.
**Patients with hearing loss at baseline: replace Km by Cm. Consider reducing the frequencyor stopping the injectable if there is a worsening of 1 grade of hearing loss compared with baseline (≥Grade 2)**				
**Hearing Aid**	Counselling.	Hearing aids usually recommended.	Certainly, hearing aids are needed. If not available, lip-reading and signing should be taught.	Hearing aids may help understanding words but additional rehabilitation needed and lip-reading and sometimes signing essential

- Five percent of the world's population has disabling hearing loss (>40dB in better ear in adults and >30 dB in children). This includes one third of 65 years old. Hence, it is important to attain a baseline record of the hearing status of a person prior to treatment initiation.
- **Patients at higher risk of ototoxicity: previous use of aminoglycoside, elderly, renal insufficiency, preexisting hearing problems, receiving other ototoxic medications.**
- The toxicity to the eighth-cranial nerve concerns the vestibule (dizziness) and the cochlea (hearing loss), and is irreversible.
- The frequencies between 500 Hz and 4000 Hz are considered to be those of a normal conversation.
- The higher frequencies (4000-8000 Hz) are the first to be affected; the frequencies of the human voice come next.
- Hearing loss becomes perceptible for patients at a frequency <4000Hz when it reaches 25-30dB (Brumett 1989). When patients mention hearing loss there is already a severe degree of loss.
- Children: above 4 years can perform pure tone audiometry. Below 4 years refer to audiologist.
- Hearing loss should always be compared to baseline measurements and ototoxicity is defined as any of:
  - (a)20dB decrease at any one frequency.
  - (b) 10dB decrease at any two adjacent frequencies.
  - (c) Loss of response at three consecutive test frequencies where responses were previ ously obtained. [23]

**Hepatotoxicity**<br>**Possible anti-TB drug causes: Z, H, Pto, Lzd, Cfz, Bdq, Mfx.**				
	**Grade 1**<br>**Mild**	**Grade 2**<br>**Moderate**	**Grade 3**<br>**Severe**	**Grade 4**<br>**Life-threatening**
**ALT (SGPT)**	1.25 – 2.5 x ULN	2.6 – 5.0 x ULN	5.1 – 10,0 x ULN	> 10.0 x ULN
**AST (SGOT)**	1.25 – 2.5 x ULN	2.6 – 5.0 x ULN	5.1 – 10,0 x ULN	> 10.0 x ULN
**Action**	Continue treatment.<br>Patients should be follow until resolution (return to baseline) or stabilization of AST/ALT elevation.	Continue treatment.<br>Patients should be followed until resolution (return to baseline).	**Stop all drugs,** including anti-TB drugs; measure LFTs weekly. Treatment may be reintroduced after toxicity is resolved.	**Stop all drugs,** including anti-TB drugs; measure LFTs weekly. Treatment may be reintroduced after toxicity is resolved.
**If JAUNDICE: Stop all anti-TB drugs until resolution**				

Consider other potential causes of hepatitis: viral (hepatitis B and C), HIV, alcohol.

Avoid potentially hepatoxic non-tuberculosis drugs.

Reintroduction of anti-TB drugs

- Check ALT/AST once a week. Reintroduce anti-TB drugs once liver enzymes return to at least Grade 2.
- Anti-TB drugs should be reintroduced in serial fashion. The least hepatotoxic drugs should be added first: Km-E-Cfz-Mxf. Then introduce the more hepatotoxic one by one every three days:Pto-H-Z while monitoring liver function tests after each one to identify the responsible drug.
- If reintroduction leads to signs of hepatotoxicity, stop the suspected drug and replace it by another if it is essential for the treatment (do not replace H and Z). Follow transaminases monthly.
- If patient is on ART and experienced Nevirapine (NVP) hepatotoxicity - should not be re-challenged with NVP.

**Peripheral neuropathy**<br>**Possible anti-TB drug causes: Lzd, Cs, H, S, Km, Cm, H, FQ, Pto/Eto, E.**<br>**Possible other causes: Diabetes Mellitus, alcohol, HIV infection, vit B deficiency, hypothyroidism. and other drugs**				
	**Grade 1**<br>**Mild**	**Grade 2**<br>**Moderate**	**Grade 3**<br>**Severe**	**Grade 4**<br>**Life-threatening**
Neurosensory alteration (including paresthesia and painful neuropathy).	Asymptomatic with sensory alteration on exam or minimal paresthesia without or minimal interference with usual social and functional activities.	Sensory alteration or paresthesia causing greater than minimal interference with usual social and functional activities.	Sensory alteration or paresthesia causing inability to perform usual social and functional activities.	Disabling sensory alteration or paresthesia causing inability to perform basic self-care functions.
**Action**	Stop offending drugs (Lzd, High dose INH). If symptoms improve after 2 weeks consider restarting Lzd at a lower dose.	**Stop Lzd anddo not reintroduce**.	**Stop Lzd and do not reintroduce.**	**Stop Lzd and do not reintroduce.**

Suggested management strategy

- All patients taking high dose INH and linezolid should receive 100 mg of pyridoxine (Vit B6) day.
- The neuropathy associated with linezolid is common after prolonged use and often extremely painful and irreversible. For this reason linezolid, should be immediately stopped and not reintroduced when symptomatic neuropathy develops (Grade 2 and above).
- Symptomatic relief:
  - Increase pyridoxine (Vit B6) to a maximum of 150 mg.
  - Nonsteroidal anti-inflammatory drugs or acetaminophen may help alleviate symptoms.
  - Tricyclic antidepressants have also been used successfully. Start amitriptyline 25 mg at bedtime. The dose may be increased to a maximum of 150 mg daily for refractory symptoms.
  - Carbamazepine may also be effective in relieving pain and other symptoms of peripheral neuropathy. **Carbamazepine is a strong inducer of CYP3A4 and should not be used with Bedaquiline or Delamanid.**

**Myelosuppression (anemia, thrombocytopenia, and/or neutropenia)**<br>**Possible anti-TB drug causes: Lzd.**<br>**Possible other causes: AZT, Cotrimoxazole, HIV Infection, chemotherapy.**				
	**Grade 1**<br>**Mild**	**Grade 2**<br>**Moderate**	**Grade 3**<br>**Severe**	**Grade 4**<br>**Life-threatening**
**Absolute neutrophil count. Normal >1500/mm^3**	1500-1000/mm^3	999-750/mm^3	749-500/mm^3	< 500/mm^3
**Haemoglobin Normal > 10.5 g/dl**	10.5 - 9.5 g/dl	9.4-8.0g/dl	7.9 – 6.5 g/dl	< 6.5 g/dl
**Platelets, Normal > 100,000/mm^3**	99,999-75,000/mm^3	74,999-50,000/mm^3	49,999-20,000/mm^3	< 20,000/mm^3
**WBC, decreased**	<Low limit normal-3000/mm^3	3000-2000/mm^3	2000-1000/mm^3	< 1000/mm^3
**Action**	Monitor carefully, and consider reduction of dose of Lzd to 300mg daily	Monitor carefully, and consider reduction of dose of Lzd to 300mg daily; in case of Grade 2 neutropenia, **stop Lzd immediately.** Restart at reduced dose, when toxicity has decreased to Grade 1.	**Stop Lzd immediately.** Restart at reduced dose when toxicity has decreased to Grade 1.	**Stop Lzd immediately.** Consider blood transfusion or erythropoietin. Restart at reduced dose when toxicity has decreased to Grade 1.
**Suggested management strategy:**<br>1. All patients taking linezolid should also be receiving at least 100 mg of pyridoxine daily. This is largely to prevent myelosuppression, but may also prevent peripheral neuropathy.<br>2. Stop the causative drug immediately.<br>3. Monitor full blood counts regularly.<br>4. Hospitalize the patient and consider transfusion if the myelosuppression is severe.				

**Optic neuritis**<br>**Possible anti-TB drug causes: Lzd, E**<br>**Possible other causes: Multiple sclerosis, quinine, herpes, syphilis, sarcoidosis, cytomegalovirus (PLHIV).**<br>The first sign of optic neuritis is usually the loss of red-green color distinction. This is best tested using the Ishihara test. Other symptoms include central scotoma (loss of central vision or blind spot).				
	**Grade 1**<br>**Mild**	**Grade 2**<br>**Moderate**	**Grade 3**<br>**Severe**	**Grade 4**<br>**Life-threatening**
Optic neuritis is inflammation of the optic nerve resulting in permanent vision loss.	Visual changes causing minimal or no interference with usual social and functional activities.	Visual changes causing greater than minimal interference with usual social and functional activities.	Visual or changes causing inability to perform usual social and functional activities.	Disabling visual loss.
**Action**	**Stop Lzd or E immediately** if there are any suspicions of optic neuritis. Do not restart.	**Stop Lzd or E immediately** if there are any suspicions of optic neuritis. Do not restart.	**Stop Lzd or E immediately** if there are any suspicions of optic neuritis. Do not restart.	**Stop Lzd or E immediately** if there are any suspicions of optic neuritis. Do not restart.

Suggested management strategy:

- Do not restart the suspected causative drug (linezolid or ethambutol).
- Refer patient to an ophthalmologist for further evaluation and management.
- Optic neuritis generally improves following cessation of offending drug, if it can be stopped early enough.
- Patients with diabetes are at increased risk for optic neuritis. They should be managed with tight glucose control as a means of prevention. Patients with advanced kidney disease are also at increased risk for optic neuritis.

Lactic acidosis
Possible anti-TB drug causes: Lzd.
Possible other causes: AZT, 3TC

	**Grade 1**<br>**Mild**	**Grade 2**<br>**Moderate**	**Grade 3**<br>**Severe**	**Grade 4**<br>**Life-threatening**
Lactate and pH	< 2.0 x ULN without acidosis	≥ 2.0 x ULN without Acidosis	Increased lactate with pH < 7.3 without life threatening consequences	Increased lactate with<br>pH < 7.3 with life threatening consequences
Action	Continue treatment regimen. Patients should be followed until resolution (return to baseline).	Stop Lzd immediately anddo not reintroduce.	Stop Lzd immediately anddo not reintroduce.	Stop Lzd immediately anddo not reintroduce.

Early signs and symptoms include **nausea, vomiting, abdominal pain, anxiety, and increased respiration rate and heart rate.** Late symptoms include lethargy, hypotension and septic shock. Early detection of lactic acidosis is important because full-blown lactic acidosis is often fatal.
Diagnosis: Analysis of an arterial blood sample showing a low pH and high lactate: anion gap, metabolic acidosis, lactate > 5 mmol/l, increased lactate/pyruvate.
If laboratory is not available, start treatment with clinical features.
Suggested management strategy:

1. Stop linezolid and NRTIs if lactic acidosis occurs. Unfortunately, it may take months for the lactic acidaemia to resolve completely even after the causative drug is stopped.
2. Hospitalize patient and monitor serum electrolytes, renal function, arterial blood gas, and lactate levels.
3. Check vital signs frequently and provide supportive care. Sodium bicarbonate therapy to correct a low pH has not been shown to be of benefit in lactic acidosis.
4. After lactic acidosis resolves, do not restart the suspected offending medication.

Pancreatitis
Possible anti-TB drug causes: Bdq, Lzd.
Other causes: gallstones, heavy and long-time alcohol use, high triglycerides.

	**Grade 1 Mild**	**Grade 2 Moderate**	**Grade 3 Severe**	**Grade 4 Life-threatening**
Pancreatitis	Not Applicable	Symptomatic and Hospitalization not indicated	Symptomatic and Hospitalization indicated	Life-threatening consequences (e.g. circulatory failure, hemorrhage,

				sepsis)
Lipase	1.1 – 1.5 x ULN	1.6 – 3.0 x ULN	3.1 – 5.0 x ULN	> 5.0 x ULN
Amylase	1.1-1.5 x ULN	1.6-2.0 x ULN	2.1-5.0 x ULN	> 5.0 x ULN
	Continue treatment regimen. Patients should be followed until resolution (return to baseline).	**Stop Lzd immediately and do not restart.**	**Stop Lzd immediately and do not restart.**	**Stop Lzd immediately and do not restart.**

The most common symptoms and signs include s**evere epigastric pain (upper abdominal pain) radiating to the back** in 50% cases, **nausea and vomiting**.

Suggested management strategy:

1. Monitor liver function tests, amylase, lipase, and full blood count
2. Provide supportive care
3. Permanently discontinue Linezolid (or Bdq if suspected to be the cause of pancreatitis).

Nausea and Vomiting
Possible anti-TB drugs: Eto/Pto, PAS, Bdq
(less common H, E, Z, Amx/Clv, Cfz, Dlm)

Grade 1 Mild	Grade 2 Moderate	Grade 3 Severe	Grade 4 Life-threatening
1 episode in 24 hours	2-5 episodes in 24 hours	>6 episodes in 24 hours or needing IV fluids	Physiologic consequences requiring hospitalization or requiring parenteral nutrition

Management and comments:

- Nausea and vomiting are common in early weeks of therapy and usually abate with time ontreatment and adjunctive therapy. Some nausea and even vomiting may need to be tolerated atleast in the initial period.
- Assess for danger signs including dehydration, electrolyte disturbances and hepatitis
- Initiate rehydration therapy if indicated and correct any electrolyte disturbances.

Initiate a step-wise approach to manage nausea and vomiting:

Phase 1:

Give a light snack (biscuits, bread, rice, tea) before the medications. Give PAS with fruit juice.
Adjust medications and conditions without lowering the overall dose:

- Give Eto or PAS twice daily
- Give PAS two hours after other anti-TB drugs.

Another strategy is to stop the responsible medicine for two or three days and then add it back gradually increasing the dose (advise the patient that the medicine will be increased back to a therapeutic dose in a manner that will be better tolerated).

Phase 2: Start antiemetic(s):

- Metoclopramide 10 mg, 30 minutes before anti-TB medications
- Ondansetron 8 mg, 30 minutes before the anti-TB drugs and again eight hours after. Ondansetron can either be used on its own or with metoclopramide. **Odansetron prolongs the QT interval; careful with bedaquiline or delamanid**
- If ondansetron is not available, promethazine can be used: promethazine25 mg PO,30 minutes before the anti-TB drugs (may be increased to 50 mg 3 times daily).
- Omeprazole or Ranitidine can also provide relief (omeprazole decreases the acid production, is also useful in the treatment of nausea).

Phase 3: Decrease dose of the suspected drug by one weight class if this can be done without compromising the regimen. It is rarely necessary to suspend the drug completely.

Note: For patients, particularly anxious about the nausea, (and with “anticipatory nausea and vomiting”) a small dose of an anti-anxiety medicine (5 mg of diazepam) can help when given 30minutes prior to the intake of anti-TB drugs. Do not give diazepam longer than 2 weeks.

Gastritis
Possible anti-TB drug causes: Eto, Pto, PAS, Cfz, FQs, H, E, and Z.

If symptoms are associated consistent with gastritis (epigastric burning or discomfort, a sour taste in mouth associated with reflux) initiate medical therapy (prolonged duration):

- Omeprazole (proton-pump inhibitors): 20 mg once daily in the evening (or 2 hrs before or 3 hours after the TB medication.
- Ranitidine 150 mg twice daily or 300 mg once daily (H2-blockers).
- Avoid the use of antacids and milk as they decrease absorption of fluoroquinolones.

Stop any nonsteroidal anti-inflammatory drugs the patient may be taking.
Diagnose and treat for *Helicobacter pylori* infections.

**Abdominal pain**<br>**Possible anti-TB drugs: Eto, Pto, Cfz, Lzd**
Abdominal pain is most commonly gastritis. However, can also be associated with serious adverse events, such as pancreatitis, lactic acidosis (Lzd) and hepatitis. If any of these are suspected, obtain appropriate laboratory tests to confirm and suspend the suspected agent.<br><br>For severe abdominal pain, stop suspected agent(s) for short periods of time (one to seven days).<br>Lower the dose of the suspected agent, if this can be done without compromising the regimen.<br>Discontinue the suspected agent if this can be done without compromising the regimen.<br><br>Severe abdominal distress has been reported with the use of clofazimine (deposition of Cfz crystal). Although these reports are rare, if this occurs, clofazimine should be suspended.

**Diarrhea**<br>**Possible anti-TB drugs: PAS, Eto/Pto**			
**Grade 1**<br>**Mild**	**Grade 2**<br>**Moderate**	**Grade 3**<br>**Severe**	**Grade 4**<br>**Life-threatening**
Mild or transient; 3-4 loose stools/day or mild diarrhea last < 1 week	Moderate or persistent; 5-7 loose stools/day or diarrhea lasting >1 week	>7 loose stools/day or bloody diarrhea; or orthostatic hypotension or electrolyte imbalance or >2L IV fluids required	Hypotensive shock or physiologic consequences requiring hospitalization
**Management**<br>1. Encourage patients to tolerate some degree of loose stools and flatulence.<br>2. Encourage fluid intake with electrolytes, if possible.<br>3. Check other causes of diarrhea. Fever and diarrhea and/or blood in the stools indicate that diarrhea may be secondary to bacterial enteritis or pseudomembranous colitis (C.difficile) related to FQ. If HIV positive, assess CD4 and think other possible causes (Cytomegalovirus (CMV), isospora, microsporidium etc).<br>4.Check serum electrolytes (especially potassium) and dehydration status if diarrhea is severe.<br>5. Treat uncomplicated diarrhea (<u>no blood in stool and no fever</u>) with loperamide 4 mg by mouth initially followed by 2 mg after each loose stool to a maximum of 10 mg per day.			

**Rash, allergic reaction and anaphylaxis**<br><br>**Possible anti-TB drugs: any drug.**			
**Grade 1**<br>**Mild**	**Grade 2**<br>**Moderate**	**Grade 3**<br>**Severe**	**Grade 4**<br>**Life-threatening**

Erythema; moderate pruritus	Extended maculopapular eruption (with or without pruritus).	Extensive papulovesicular eruption, palpable purpura, moist desquamation or ulcerations.	Exfoliative dermatitis, mucous membrane involvement or erythema multiforme or suspected Stevens-Johnson or cutaneus necrosis requiring surgery.
Continue the treatment and add ancillary medicines*.	Continue the treatment and add ancillary medicines*. Close monitoring. Stop the therapy if rash is worsening.	**Stop all therapy pending resolution of reaction.** Do not reintroduce the offending agent.	**Stop all therapy pending resolution of reaction**. **Hospitalization is required. Do not reintroduce the offending agent.**

Management:

1. For serious allergic reactions (Grade 3-4), stop all therapy pending resolution of reaction. In the case of anaphylaxis manage with standard emergency protocols (including adrenaline). If Steven Johnson Syndrome treat with IV corticosteroid, IV fluids and IV broad spectrum antibiotic.

Suspend permanently any drug identified to be the cause of a serious reaction. Any drug that resulted in anaphylaxis or Steven–Johnson syndrome should never be reintroduced

2. Eliminate other potential causes of allergic skin reactions (like scabies or other environmental agents).

3. *Anciliary medicines:

For minor dermatologic reactions, various agents may be helpful and allow continuation of the medication, such as

• Antihistamines.
• Hydrocortisone cream for localized rash.
• Prednisone in a low dose of 10 to 20 mg per day for several weeks.
• Dry skin may cause itching (especially in diabetics), liberal use of moisturizing lotion is recommended. Dry skin is a common and significant problem with clofazimine.

4. Once the minor rash resolves, reintroduce remaining drugs, one at a time with the one most likely to cause the reaction last. Consider not reintroducing even as a challenge any drug that is highly likely to be the cause. The order or reintroduction can be: H, Z, Eto/Pto, FQ, Cs, E, PAS, Km (or Am/Cm)

Arthralgia/Arthritis
Possible anti-TB drug: Z (less frequently FQ, Bdq).

	**Grade 1 Mild**	**Grade 2 Moderate**	**Grade 3 Severe**	**Grade 4 Life-threatening**
Arthralgia (joint pain)	Mild pain not interfering with function	Moderate pain, analgesia and/or pain interfering with	Severe pain; pain and/or paininterfering	Disabling pain

		function but not with activities of daily life (ADL)	with ADL.	
Arthritis (inflammation involving a joint)	Mild pain with inflammation, erythema or joint swelling, but not interfering with function.	Moderate pain with inflammation, erythema or joint swelling; interfering with function, but not with activities of daily life (ADL).	Severe pain with inflammation, erythema or joint swelling and interfering with ADL.	Permanent and/or disabling joint destruction.

Management:

- Give NSAIDs: ibuprofen 600 mg 3 times a day.
- Rest the joint.
- Initiate therapy with nonsteroidal anti-inflammatory drugs:
  Ibuprofen 400 to 800 mg three times a day or Indomethacin 50 mg twice daily
- Lower the dose or discontinue the suspected agent (most commonly pyrazinamide) if this can be done without compromising the regimen.
- Uric acid levels may be elevated in patients on pyrazinamide. There is little evidence to support the addition of allopurinol for arthralgia, although if gout is present it should be used.
- If acute swelling, redness and warmth are present in a joint, consider aspiration for diagnosis of gout, infections, autoimmune diseases, TB arthritis etc.

Psychosis
Possible anti-TB drugs:Cs, H, FQ, Eto/Pto.

**Grade 1**<br>**Mild**	**Grade 2**<br>**Moderate**	**Grade 3**<br>**Severe**	**Grade 4**<br>**Life-threatening**
Mild psychotic symptoms	Moderate psychotic symptoms (e.g., disorganized speech; impaired reality testing).	Severe psychotic symptoms (e.g., paranoid; extreme disorganization); hospitalization not indicated.	Acute Psychosis (suicidal ideation, maniac status, hallucinations). Life-threatening consequences, threats of harm to self or others; **hospitalization indicated**

Management

The most likely drug is cycloserine followed by high dose isoniazid.

1. **Stop the suspected agent for a short period of time** (1–4 weeks) while psychotic symptoms are brought under control.

2. Always check creatinine in patients with new onset psychosis. A decrease in renal function can result in high blood levels of cycloserine, which can cause psychosis.

3.If moderate to severe symptoms persist, initiate **antipsychotic therapy (haloperidol).**
4.**Hospitalize in a ward with psychiatric expertise if patient is at risk to himself/herself or others**.
5.Increase pyridoxine to the maximum daily dose (200 mg per day).
6.Lower the dose of the suspected agent (most commonly cycloserine to 500 mg a day).
7.Discontinue the suspected agent if this can be done without compromising the regimen.
8.Once all symptoms resolve and patient is off cycloserine, antipsychotic therapy can be tapered off. If cycloserine is continued at a lower dose, anti-psychotic therapy may need to be continued and any attempts of tapering off should be done after referring to a psychiatrist.

Some patients will need to continue antipsychotic treatment throughout DR-TB treatment (and discontinued gradually upon completion of treatment).

Previous history of psychiatric disease is not a contraindication to cycloserine, but its use may increase the likelihood of psychotic symptoms developing during treatment. Avoid if there is an alternative.
Psychotic symptoms are generally reversible upon completion of DR-TB treatment or cessation of the offending agent.

Depression
Possible anti-TB drugs: Cs, FQ, H, Eto/Pto
Other causes: Psychological and socioeconomic circumstances, chronic disease.

**Grade 1**<br>**Mild**	**Grade 2**<br>**Moderate**	**Grade 3**<br>**Severe**	**Grade 4**<br>**Life-threatening**
Mild depressive symptoms; and/or PHQ9 depression score 1-9.	Moderate depressive symptoms; limiting instrumental Activities of Daily Life (ADL); and/or PHQ9 depression score 10-14.	Severe depressive symptoms; limiting self-care ADL; hospitalization not indicated; and/or PHQ9 depression score 15-19.	Life-threatening consequences, threats of harm to self or others; PHQ9 depression score 20-27; and/or hospitalization indicated

Management:
- Anti-TB Therapy may contribute to depression. Depressive symptoms may fluctuate during the therapy.
- Assess and address underlying emotional and socioeconomic issues.
- Provide psychosocial support (for the patient and family).
- If depression is significant, initiate antidepressant therapy (amitriptyline, fluoxetine).
- **Avoid serotonin reuptake inhibitors and tricyclic antidepressant with Lzd (risk of serotonin syndrome).**
- Lower the dose of the suspected agent if this can be done without compromising the regimen. (reducing the dose of cycloserine to 500 mg or one weight band daily)
- Discontinue the suspected agent if this can be done without compromising the regimen.

**Seizures**<br>**Possible anti-TB drugs: Cs, H, FQ, Imp/Cln.**
First **address other causes of seizures:** infection, epilepsy, meningitis, encephalitis, alcohol withdrawal, hypoglycemia, cerebrovascular accident, malignancy or toxoplasma (in PLHIV).<br>Then:<br>1. Hold cycloserine and isoniazid pending resolution of seizures.<br>2. Initiate anticonvulsant therapy (carbamazepine, phenytoin or valproic acid are most commonly used).<br>3. Increase pyridoxine to the maximum daily dose (200 mg per day).<br>4. Check serum electrolytes including potassium, sodium, bicarbonate, calcium, magnesium and chloride (and replace accordingly).<br>5. Check creatinine level. A decrease in renal function can result in high blood levels of cycloserine, which can cause seizures. Adjusting the dose of cycloserine in the presence of low creatinine may be all that is needed to control the seizures<br>6. When seizures have resolved, restart medications one at a time. Cycloserine should not be restarted unless itis absolutely essential to the regimen. If cycloserine is reinitiated, start a dose one weight band lower.<br><br>Notes:<br>The anticonvulsant is generally continued until DR-TB treatment is completed or suspected agent is discontinued.<br><br>History of previous seizure disorder is not a contraindication to the use of agents listed hereif a patient's seizures are well controlled and/or the patient is receiving anticonvulsant therapy. (Do not include cycloserine if an alternative drug is available.)

**Hypothyroidism**<br>**Possible anti-TB drugs: Eto/Pto, PAS**			
**Grade 1**<br>**Mild**	**Grade 2**<br>**Moderate**	**Grade 3**<br>**Severe**	**Grade 4**<br>**Life-threatening**
Sub-clinical hypothyroidism (TSH 6-10mIU/l, T4 free normal)	Simple Hypothyroidism without complications. Treatment required (TSH > 10 mIU/l)	Severe Hypothyroidism with clinical symptoms. Urgent treatment	Myxedematous coma.
**Management**<br>Start treatment when TSH > 10 mIU/l<br>Dose of levothyroxine:			

Adults: 1.2- 1.4 μg/kg/day (50-200 μg daily)
Children: 4-5 μg/kg/day (max 200 μg/dia). Children need higher dose due to hormone metabolism.

1. Most adults will require 100-150 μg of levothyroxine daily. Start levothyroxine in the following manner:
   - Young healthy adults can be started on 75–100 μg daily
   - Older patients should begin treatment with 50 μg daily
   - Patients with significant cardiovascular disease should start at 25 μg daily
2. Monitor TSH every every 30 to 45 days and increase the dose by 25 μg until TSH normalizes (TSH < 5 mIU/l). Adjust the dose more slowly in the elderly and in patients with cardiac conditions.

Note: it could be considered to start treatment with TST >6 -10 mIU/l with low dose of levothyroxine (25 to 50 μg).
Thyroid dysfunction resolves upon discontinuation of the causative agent. Hormone replacement must continue at least 2 to 3 months after DR-TB treatment is completed.

10. Management of close contacts of DR-TB patients

10.1 Contact Investigation

Household contact is defined as a person who shared the same enclosed living space as the index case for one or more nights or for frequent or extended daytime periods during the 3 months before the start of the current treatment episode.

Close contact of DR-TB is defined as a person who is not in the household but who shared an enclosed space, such as a social gathering place, workplace, or facility, with the index case for extended daytime periods during the 3 months before the start of the current treatment episode.

All household or close contacts of known DR-TB patients should receive careful TB screening and clinical follow-up for a period of at least two years (every 6 months or more frequently if required).

Attention should be paid to those contacts who are below the age of 5, those who have HIV infection,pregnant women and diabetic patients.

Contact management is crucial and should be guided by the clinical status of the contact i.e. whether symptomatic or asymptomatic.

All symptomatic adult contacts and all child contacts of DR-TB patients need to undergo clinical screening: including Xpert MTB/RIF, SL-LPA, Culture & phenotypic DST and Chest X-Ray.

It shouldbe notedthat bacteriological confirmation may not always be possible in young children and HIV positive patients. A negative test in a symptomatic child or HIV patient does not exclude disease.

In contacts with presumptive extra-pulmonary TB, bacteriological confirmation is often quite challenging. Extra-pulmonary samples may be referred for Xpert MTB/RIF test and culture but they are often negative.Patients with extra-pulmonary TB may have subtle pulmonary involvement, hence it is important to refer sputum samples for Xpert MTB/RIF test, LPA and culture/pDST as well.

If active TB is strongly suspected, an empirical regimen based on the resistance pattern of the index patient is warranted, particularly for children, immune compromised adults and patients with EP-TB.

10.2 Prophylaxis in children and adults exposed to DR-TB

Generally, chemoprophylaxis in children or adults exposed to DR-TB who are asymptomatic is not recommended as there is little evidence and no agreed consensus on the optimal regimen for prophylaxis. More evidence might be available in coming years from ongoing randomize control trials using fluoroquinolone or delamanid based regimens.

A reasonable approach is to monitor all the contacts (children and adults) closely for 24 months in order to identify and treat developing TB promptly.

11. Active TB Drug Safety Monitoring and Management (aDSM)

- ***All adverse events (AEs)*** *need to be* ***detected and managed*** *in a timely manner.*
- ***All serious Adverse Events*** *(SAEs) should be registered and* ***reported within 24 hours.***
- ***Reporting SAEs is mandatory*** *for all clinicians and treatment centers managing DR-TB patients.*
- ***Standardized data on SAEs*** *would be systematically collected, collated and reported using and adapting the existing reporting system.*

aDSM is intended to be an integral component of the programmatic management of drug-resistant TB (PMDT). Its rationale is based on recent developments in DR-TB treatment, particularly the approval for use of new medicines ahead of the completion of Phase III trials, increased use of repurposed drugs for DR-TB treatment and the development of novel second-line anti-TB regimens. Such approaches need careful monitoring for drug-related harm, some of which may not have been described as yet. [3]

11.1 aDSMdefinition and objective

The term 'active TB drug-safety monitoring and management' (aDSM) defines active and systematic clinical and laboratory assessment of patients while on DR-TB treatment.
aDSM applies to patients on treatment with: (i) new anti-TB drugs; (ii) novel RR/MDR-TB regimens; or (iii) extensively drug-resistant TB (XDR-TB) regimens, in order to detect, manage and report suspected or confirmed drug toxicities.
The overall objective of aDSM is to reduce risks from drug-related harm in patients on second line treatment for drug-resistant TB and to generate standardized aDSM data to enable causality assessment for SAEs, determine their frequency (rates) and detect signals. This will contribute to future policy updates on the use of such medicines.
Pharmacovigilance is one of the components of aDSM, and is defined by WHO as "the science and activities relating to the detection, assessment, understanding and prevention of adverse effects or any other drug-related problem" [3]

11.2 Key steps to implement aDSM

Based on the experience of successful implementation of other care and monitoring components of PMDT programs, eight key steps have been identified for programs to follow when introducing aDSM:

1. Create a national coordinating mechanism for aDSM.
2. Develop a plan for aDSM.
3. Define management and supervision roles and responsibilities.
4. Create standard data collection materials.
5. Train staff for collection of data.
6. Define schedules and routes for data collection and reporting.
7. Consolidate aDSM data electronically.
8. Develop (or use existing) capacity for signal detection and causality assessment.

Ideally, all eight steps should be in place before patients are enrolled on treatment with new drugs, novel RR/MDR-TB regimens or XDR-TB regimens. As this may not always be feasible, two steps – step (4) create standard data collection materials and step (5) train staff for collection of data – are essential ahead of any patient enrolment.

The responsibility for the coordination of aDSM at the national level should be assigned to an existing TB expert body, such as the national DR-TB committees as well as PV unit.These committees should primarily have scientific and clinical expertise for DR-TB care and drug safety monitoring and also include expertise important for management and communication (e.g., funding, advocacy, patient representation).

11.3 Strategy for aDSM implementation

- A national coordinating mechanism for aDSM will be established by the inclusion of NAFDAC in the existing DR-TB committee/National consilium of experts.
- Provide IEC materials at all service delivery points.
- Integrate review of pharmacovigilance issues into the quarterly State/Zonal/National meetings.
- Conduct bi-annual causality assessment .
- Recording and reporting of AE and SAE on the aDSM.
- Conduct training of health care workers on aDSM.
- Collect completed yellow and aDSM forms quarterly and submit to NAFDAC and NTBLCP.
- Analyze aDSM reports monthly/quarterly at all levels.
- Communicate and disseminate finding(s) to relevant stakeholders after the meetings.
- Utilize information for future policies on drug safety and rational use.

10.4 Common definitions used in aDSM

Adverse drug reaction (ADR) is a response to a TB medicine which is noxious and unintended, and which occurs at doses normally used in humans. This term is used to qualify adverse events that are thought to be related to a drug.

Adverse event (AE) is any untoward medical occurrence that may present in a TB patient during treatment with a pharmaceutical product, but which does not necessarily have a causal relationship with this treatment.

Serious adverse event (SAE) is any AE that is:

	**SERIOUS ADVERSE EVENTS (SAEs)**
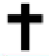	Fatal
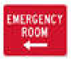	Immediately life threatening
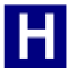	Leading to hospitalization or prolongation of hospitalization
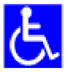	Leading to a persistent or significant disability
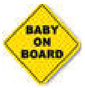	Congenital anomaly
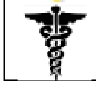	Otherwise medically important, necessitating an intervention to prevent one of the above listed outcomes.

SAEs may require a drastic intervention, such as termination of the drug suspected of having caused the event. All the centers should report all SAEs within 24 hours (Core package of aDSM).

Adverse event of special interest* is an AE documented to have occurred during clinical trials and for which the monitoring team is specifically sensitized to report regardless of its seriousness, severity or causal relationship to the TB treatment. The centers that offer intermediate and advanced packages of aDSM will include all AEs of special interest in their reporting.

Adverse event of clinical significance* is an AE which is either (i) serious, (ii) of special interest, (iii) leads to a discontinuation or change in the treatment, or (iv) judged as otherwise clinically significant by the clinician. The centers that offer the advanced package of aDSM will include all AEs of clinical significance in their reporting.

Adverse event leading to treatment discontinuation or change in drug dosage: is one that leads a clinician to stop, interrupt temporarily or change the dosage of one or more drugs, regardless of its seriousness, severity, or causal relationship to the TB treatment.

Medication errors: defined as unintended mistakes in the prescribing, dispensing and administration of a medicine that could cause harm to a patient (e.g. wrong drug prescribed, overdose) must be managed on a case by case basis. Hospitalization should be considered as appropriate. [19]

***Adverse Events of clinical significance or special interest in aDSM:**
1. All serious adverse events (SAEs).
2. All AEs of special interest (suggested list from WHO):

- Peripheral neuropathy (paresthesia).
- Psychiatric disorders and central nervous system toxicity (e.g. depression, psychosis, suicidal intention, seizures).
- Optic nerve disorder (optic neuritis) or retinopathy.
- Ototoxicity (hearing impairment, hearing loss).

- Myelosuppression (manifested as anemia, thrombocytopenia, neutropenia or leukopenia).
- Prolonged QT interval (Framingham correction).
- Lactic acidosis.
- Hepatitis (defined as increases in alanine aminotransferase (ALT) or aspartate aminotransferase (AST) ≥5x the upper limit of normal (ULN), or increases in ALT or AST ≥3x ULN with clinical manifestations, or increases in ALT or AST ≥3x ULN with concomitant increase in bilirubin ≥1.5 x ULN).
- Hypothyroidism.
- Hypokalemia.
- Pancreatitis.
- Phospholipidosis.
- Acute kidney injury (acute renal failure).

3. Adverse events leading to treatment discontinuation or change in drug dosage, defined as all AEs regardless of their seriousness, severity, or causal relationship to the MDR-TB treatment, leading to a discontinuation of MDR-TB treatment, including permanent and temporary treatment interruption, or changes in drug(s) dosage(s) or drug regimen, as decided by the clinician.

4. Adverse events not listed above but judged as otherwise clinically significant by the clinician, defined as all AEs regardless of their seriousness, severity, or causal relationship to the RR/MDR-TB treatment, not pertaining to one of the above-mentioned category but considered of clinical significance by the treating physician.

11.5 Levels of monitoring in aDSM

In addition to drug-safety monitoring, aDSM also incorporates a component that promotes the systematic clinical and laboratory assessment for early detection and prompt management of all ADRs and AEs regardless of their seriousness, severity or whether they have been attributed to any of the medicines to which the patient is exposed. All the AE should be registered and recorded in the patient's file.
Regarding reporting, there are different **aDSM packages according to the AE reported:**

1. Core package: requires the reporting of only all serious adverse events (SAEs). It iscompulsory for all DR-TB treatment centers. The report should be done within 24 hours.
2. Intermediate package: includes SAEs as well as AEs of special interest (Selected treatment centers called sentinel sites).
3. Advanced package: includes all AEs of clinical significance.

Standardized data on SAEs would be systematically collected, collated and reported using and adapting the existing reporting system (Yellow form, aDSM form, electronic system) or from registers (laboratory registers, TB treatment registers) and the patient's medical records/treatment cards as necessary.

11.6 Causality assessment

Causality assessment is the evaluation of the likelihood that a TB medicine was the causative agent of an observed adverse reaction. The formal causality assessment is performed by expert at the national pharmacovigilance center.

Causal relationship is a relationship between an exposure (A) and an event (B) in which A precedes and causes B. This may refer to the causal association between an exposure to a TB medicine and the occurrence of an adverse reaction.
Signal is reported information on a possible causal relationship between an adverse event and a TB medicine, the relationship being unknown or incompletely documented previously or representing a new aspect of a known association. The information may arise from one or multiple sources that are judged to be of sufficient likelihood to justify verification.

Steps for Causality assessment:

- Take complete medical and drug history.
- Document adverse events including clinical description, lab results, date of onset/end.
- Determine evolution of events, severity/seriousness and outcome.
- Adopt the ANRS/recommended PV – TB scale for grading AEs and SAEs.
- Prevent or manage AE/SAE based on recommended actions (Refer to severity grading chart).
- Establish association between concomitant medicines (herbal medicines and supplements), food and co-morbid conditions.
- Establish Potential drug toxicities and overlapping adverse effects in the treatment of HIV and drug-resistant TB.
- Determine and detect signals and causal relationship.
- Document data/finding on the aDSM form and e-TB manager.

11.7 Recording and reporting of adverse events (AEs)

Collect routine safety data using recording and reporting systems (i.e. eTB manager/aDSM or yellow forms) or from registers (laboratory registers, TB/DR-TBtreatment registers) and patient's medical records/treatment cards as necessary.
Routine data collection on safety of the DR-TB regimens includes registration of all SAEs and AEs of special interest – in line with the intermediate aDSM package.
For all patients who started on DR-TB treatment that experience an SAE or AE of special interest the following information will be recorded:

- DR-TB number/ART code.
- Type of SAE (congenital anomaly or birth defect, persistent or significant disability, death, required hospitalization, prolonged hospitalization, life threatening).
- Type of AE of special interest.
- Onset date of adverse event.
- Clinical action taken (including provision of ancillary drugs, re-challenge), and
- Result of the causality assessment (whether the SAE is attributable to one or more anti-TB or concomitant drugs).

Notification of adverse events occurs as follows:
Immediate transmission (within 24 hours of awareness) of serious adverse events, drug-exposed pregnancies and medication errors (with or without associated AEs/SAEs) to the pharmacovigilance (PV) unit and NTP/National DR-TB committee.
Routine recording and reporting of all other AEs (non-serious) should be recorded on the patient's file and reported using the AE Form.

12. Procurement supply and management of shorter regimen commodities

The success of Shorter Regimen depends on the uninterrupted availability of all the relevant commodities: medicines, lab equipment, diagnostics, reagents, and recording and reporting materials to fulfill the six rights of a logistic system.

To achieve this, the following should be conducted:

1) **Product selection**:
   - Select appropriate medicines / lab commodities and consumables for the shorter or individualized regimen in line with the National guideline.

2) **Quantification & Forecasting:**
   - Build and document assumptions for forecasting and quantification for procurement of medicines, lab commodities using the QuanTB or an excel based tool.

3) **Warehousing and Distribution**:
   - Store all health products under optimal warehousing conditions in line with good storage practices.
   - Quarantine and dispose all damaged and expired DR-TB health productsin line with the provisions of the procurement and supply chain management of health products policy guideline.
   - DR-TB health products would be distributed/transported in line with the existing in-country mechanisms for this purpose.

4) **Rational Drug Use:**
   - Select good quality and cost effective ancillary medicines.
   - Provide ancillary medicines on time for the management and prevention of SAEs and AEs from registered pharmacy stores.

5) **Logistics Management Information Systems (LMIS)/Inventory Management:**
   - Utilize the existing LMIS tools and templates to collect and collate data/reports to inform resupplies/issues, quantification, forecasting and shipment planning of DR-TB health products.
   - Provide inventory control tools for recording all transactions involving DR-TB health products at the warehouse/facility.

6) **Quality assurance:**
   - Routine quality assurance of DR-TB medicines in selected warehouses/facilities in-country will be conducted to ascertain the efficacy/quality of DR-TB medicines issued to the patients.

13.Supervision, monitoring and evaluation for DR-TB

TheDR-TBsupervisory,monitoringandevaluationsystem(SMEsystem) is an integral part of NTBLCP structure.However,toensurethatplannedactivitiesarecarriedout,additionalactivitiesspecificto DR-TBhavebeenrecommended.

For more information refer to: Federal Ministry of Health of Nigeria - Department of Public Health (FMOH), National Tuberculosis and Leprosy Control Program (NTBLCP), Guidelines for programmatic and clinical management of drug resistant tuberculosis in Nigeria, second edition. 2016 (page 92-97).

13.1 Information Flow System

The NTBLCP M&E information flow system starts from the health facilityto the central unit of the program. The M&E responsibilities at the various levels are summarized in pages 94-96 of PMDT guidelines 2016.

13.2 Supervision

Supervisionis a process of providing technical support aimed at improvingstaff competenceandeffectiveness through observation, discussion, and on-the-job training.

- TheStateTBLteam & a representative of consilliumwillconduct monthly supervision ofDR-TBtreatmentcenters as well as monitoring of patients in community care.
- The national program shall also conduct joint quarterlysupervisory visitsto theDR-TB treatmentcenters and health facilities providing community DR-TB treatment.

13.3 Program Monitoring

Monitoring is an essential component of a good control program. The following activities are vital for monitoring DR-TB activities in line with the National guidelines:

- The consillium of DR-TB experts including medical officers in the out patients LGA clinics should meet quarterly and when necessary to review all DR-TB cases
- The state DR-TB team should meet quarterly to validate their records and discuss challenges, lessons learned and develop plan of action
- All patients on community care should be medically evaluated at least once a month.
- The National DR-TB Committee meets quarterly to review the progress of DR-TB program implementation.
- The annual program review meeting is a forum where key stakeholders meet to review the progress made, challenges encountered and come up with strategies for the new reporting year.

13.4 DR-TB Program Indicators

Indicators are pointers to program performance. There are three overarchingindicators for monitoring PMDT program contained in national PMDT guideline 2016:

- DR-TB case notification indicators.
- DR-TB enrolment for care indicators.
- DR-TB case holding indicators.

13.4.1 End-TB strategy indicators

In addition, three of the top ten End-TB strategy indicators not captured in the guideline shall be reported by NTBLCP as indicated in table below.

Table 13: DR-TB Program Indicators (End-TB indicators)

N	Indicator	Definition	Formula	Frequency	Source document
1	Treatment coverage, new TB drugs.	Proportion of TB patients treated with regimens that include new TB drugs divided by the number of notified patients eligible for treatment with new TB drugs.	Number of TB patients treated with regimens that include new TB drugs divided by the number of notified patients eligible for treatment with new TB drugs.	**Quarterly**	**Treatment register, Xpert MTB/RIF Line list**
2	Drug susceptibility testing (DST) coverage for DR-TB patients.	Proportion of TB patients with DST results for at-least rifampicin divided by total number of notified (new & retreatment) cases in the same year.<br><br>*DST coverage includes results from molecular test (Xpert MTB/Rif/LPA) as well as conventional phenotypic DST result.	Number of TB patients with DST results for at-least rifampicin divided by total number of notified (new & retreatment) cases in the same year.	**Quarterly**	**DR-TB facility register, Xpert MTB/RIF Line list**
3	Contact investigation coverage	Proportion of contacts of people with bacteriologically confirmed TB who were evaluated for TB, divided by the number eligible.	Number of contacts of people with bacteriologically confirmed TB who were evaluated for TB, divided by the number eligible,	**Quarterly**	**DR-TB facility register, Xpert MTB/RIF Line list**

Source: Table 1.0-End TB Strategy indicators.

13.4.2Indicatorsattheendofintensivephase(sixthmonthinterimoutcome)

- Proportionofeligible DR-TBpatients startedonshorter treatment regimens withanegative cultureresultattheendof the sixthmonthoftreatment.
- Proportionofeligible DR-TBpatients startedon shorter treatment regimensbutdiedattheend ofsixthmonthoftreatment.
- Proportionofeligible DR-TBpatientsstartedon shorter treatment regimensbutlosttofollow-upattheendofsixthmonthoftreatment.
- Proportionofeligible DR-TBpatientsstartedon shorter treatment regimensbutnotevaluated attheendofsixthmonthoftreatment.

- Proportionof DR-TBpatientsstartedon shorter treatment regimens andhadtestdone butcultureresultnotavailableattheendof sixth monthoftreatment.
- Proportion(%)ofculturepositiveDR-TBpatientsinitiatedonDR-TBtreatmentlater foundnottohaveDR-TBbythesixthmonthoftreatment.

For more information refer to PMDT guidelines 2016-page 95.

13.4.3Indicatorsattheendoftreatment

- Proportion(%)ofDR-TBpatientscured(DR-TBcurerate).
- Proportion (%) of DR-TB patients declared treatment completed(RR/MDR-TB treatment completionrate).
- Proportion(%)ofDR-TBpatientssuccessfullytreated(DR-TBtreatmentsuccessrate).
- Proportion(%)ofDR-TBpatientsthatfailedtreatment(DR-TBtreatmentfailurerate).
- Proportion(%)ofDR-TBpatientsthatdied(DR-TBdeathrate).
- Proportion(%)ofDR-TBpatient'slosstofollow-up(DR-TBlosttofollow-uprate).
- Proportion(%)ofDR-TBpatientsnotevaluated(DR-TBnotevaluated rate).
- Proportion(%)ofDR-TBpatientsstillontreatment(DR-TBstillontreatmentrate).

For more information refer to PMDT guidelines 2016-page 96.

13.4.4Indicators to monitor active TB drug safety monitoring and management (aDSM).

The indicators in table 14 are used to monitor the adverse drug reaction for patients receiving DR-TB treatment regimens – shorter or individualized.

Table 14: Active TB Drug Safety Monitoring & Management

No	INDICATOR	DEFINITION	FORMULA	SOURCE DOCUMENT/FREQUENCY	NOTE
1	Coverage process	Target RR/MDR-TB patients included in cohort event monitoring (CEM)	Proportion of TB patients started on target treatment included in CEM during period of assessment divided by TB patients started on target treatment during the period of assessment eligible for CEM	CEM register, Second line treatment register. ETB-manger<br><br>Quarterly	To be computed during the period of recruitment but not in the post-treatment observation phase.
2	Completeness process	Time to stop target drug	The difference in days between the date of start of treatment with a target	CEM register<br><br>12 monthly	Stratify by reason for stopping (e.g. success, died, treatment failed, loss to follow up, exclusion criterion developing after start of treatment such as

			drug and the date of the stopping the target drug.		pregnancy).
3	Serious adverse effect	RR-/MDR-TB patients included in CEM with any serious adverse event	Proportion of TB patients included in CEM during the period of assessment with ≥ 1 serious adverse event divided by TB patients included in CEM during the period of assessment.	CEM register.<br><br>Quarterly.	Tobecomputed during theperiodof patientrecruitment andduringthepost-treatmentobservation outcome(Indicate hospitalizations, disability)
4	Adverse reactions associated with target treatment.	Frequency ofADRs associated withthe target treatment.	Proportion ofADRsattributedto targettreatmentamong patientsonCEMdivided by TBcasesincludedin CEMduringtheperiod ofassessment.	CEM register,<br><br>Quarterly.	To be computed during the period of patient recruitment and during the post-treatment observation phase.Onlytobereported aftercausality assessment(e.g.de-challenge, re-challenge)suggests thetargettreatmentasthecausativeagent (certain,probableor possible).<br>Thesamepatientmay haveseveralADRs (thereforetheunitofmeasurementis theADRandnotthe patients).
5	Adverse reactions associatedwith target treatment	Time to development of adverse drug reactions associated with target treatment	Difference in days between the days of start of target treatment and the date of the first detected onset of ADR attributed to it.	CEM register<br><br>6monthly.	Tobecomputedduring theperiodofpatient recruitmentandduring thepost-treatment observationphase.

13.5 Routine Data Quality Assurance (RDQA)

The RDQA for TB is intended:

- To assess and measure rapidly the quality of data recording/reporting systems on a regular basis and monitor/improve data recording/reporting systems.

- To provide self-assessment by the program; measures the quality of the data collection system; and offer flexible use for monitoring and supervision or to prepare for an external audit.

Using appropriate RDQA tool/checklist DQA shall be performed at various level to assess/monitor the quality of data at the health facility, LGA, State level. The potential users of the RDQA tool includenational/state TB program managers and health staff involved in TB.

13.6 Recording and Reporting Tool

The recording and reporting tools used in DR-TB control program include:

- Form 01: Second line TB treatment card.
- Form 02: Second line TB treatment register.
- Form 03: Request for examination of biological specimen for TB.
- Form 04: Laboratory register for Xpert MTB/RIF, culture, and drug susceptible testing (DST).
- Form 05: DR-TB patient referral/transfer form.
- Form 06: Discharge form (from DR-TB treatment center to DOT facility).
- Form 07: Patient hard card.
- DR-TB 07 Quarterly Xpert MTB/RIF summary form.
- DR-TB 08 M(X)DR-TB monthly notification form.
- DR-TB 09 Quarterly Report on DR-TB case registration.
- DR-TB 10 Six-month interim outcome.
- DR-TB 11 Annual report interim outcome.
- DR-TB 12 24-month final outcome.

See R&R tools for DR-TB in Annex section of the addendum.

13.7 Evaluation

Structured interpretation of patient progress reports in line with the expected impact is required in assessing performance.

The effectiveness of the shorter regimen will be assessed at different intervals:

- **Cohort review:** will be done quarterly.
- **Six-month Interim outcome:** to be completed 9-12 month after the closing day of the cohort:
    - Patients lost to follow-up in month six should be <5%
    - Patients with an unknown culture and smear status should be <10%
    - Patient with culture conversion within the first six months should be >90%
- **12-month Preliminary outcome:** to be completed 12-15 month after the closing date of the cohort:
    - Patient lost to follow-up in months 7-11 to be <10%.
    - Patient with an unknown culture status at 11 month to be <10%.
- **Final outcome (24-month outcome)**: to be completed 24 months (annual cohort) after receipt of culture results:
    - Patients who have a final outcome of treatment success (cured and completed treatment) to be >90%
    - Patient with the outcome cured among successfully treated to be>85%.
    - Patient who are lost to follow-up at the end of the treatment to be <7%.

13.8 Treatment Outcome Definitions

All patients should be assigned a final outcome when they complete the treatment regimen and/or at the end of the cohort reporting period.

Table 15: Treatment outcome definitions

**Cured**	A DR-TB patient who completed treatment without evidence of failure and has three or more consecutive cultures taken at least 30 days apart that are negative after the intensive phase.
**Treatment completed**	A DR-TB patient who completed treatment without evidence of failure BUT does not have three or more consecutive cultures taken at least 30 days apart which are negative after the intensive phase.
**Treatment success**	The sum total of cured and treatment completed.
**Treatment failure**	A DR-TB patient for whom treatment was terminated or need for permanent regimen change of at least two anti-TB medicines because of:<br>o Lack of conversion[a] by the end of intensive phase; or<br>o Bacteriological reversion[b] in the continuation phase after conversion[a] to negative; or<br>o Evidence of additional acquired resistance to fluoroquinolones or second-line injectable drugs; or<br>o Adverse drug reactions.
**Died**	A DR-TB patient who dies for any reason during the course of treatment.
**Loss to follow-up**	A DR-TB patient whose treatment was interrupted for two consecutive months or more.
**Not evaluated**	A DR-TB patient for whom no treatment outcome is assigned. (This includes patients "transferred out" to another treatment unit and where the treatment outcome is unknown to the reporting unit).

[a] **Conversion (to negative):** Culture is considered to have converted to negative when two consecutive cultures, taken at least 30 days apart, are found to be negative.

[b] **Reversion (to positive):** Culture is considered to have reverted to be positive when, after an initial conversion, two consecutive cultures, taken at least 30 days apart are found to be positive. For the purpose of defining treatment failure, reversion is considered only when it occurs in continuation phase. [4]

References

1. WHO. The evaluation and safety of a shorter standardized treatment regimen for multidrug-resistant tuberculosis (GDI). 2015 May.

2. WHO. Treatment guidelines for drug resistant tuberculosis. 2016 May.

3. WHO. Active tuberculosis drug-safety monitoring and management (aDSM). Framework for implementation. 2015 Nov.

4. WHO. Companion handbook to the WHO guidelines for the programmatic management of DR-TB. 2014, updated 2015 and 2016.

5. WHO. Policy implementation package for new TB drug introduction. 2014.

6. WHO. Global Tuberculosis report. 2016.

7. WHO. The use of bedaquiline in the treatment of MDR-TB. 2013 Interim guidance.

8. WHO. The use of delamanid in the treatment of MDR-TB. 2014 Interim guidance.

9. The Sentinel Project. The use of delamanid and bedaquiline for children with drug-resistant tuberculosis. 2016.

10. The Sentinel Project. Management of Multidrug-Resistant Tuberculosis in children: a field guide. 2015.

11. WHO. Guidance for national tuberculosis programmes on the management of tuberculosis in children. 2015.

12. Federal Ministry of Health of Nigeria Department of Public Health (FMOH), National Tuberculosis and Leprosy Control Programme (NTBLCB). Guidelines for programmatic and clinical management of drug resistant tuberculosis in Nigeria, second edition. 2016

13. Federal Ministry of Health of Nigeria (FMOH), The national strategic plan for Tuberculosis Control 2015-2020/2015.

14. S M Graham et al, Desk guide for diagnosis and management of TB in children, Paris, France: The International Union of Tuberculosis and Lung Disease, Third edition, 2016www.theunion.org

15. MDR-TB weight dosing chard for children-Sentinel Projecthttp://sentinel-project.org

16. WHO. The use of Delamanid in the treatment of multidrug-resistant tuberculosis in children and adolescents (Interim policy guidance)-October 2016.

17. GLI Model TB algorithms-Global Laboratory Initiative-March 2017

18. National Agency for Research in AIDS and Hepatitis/France/Division of Microbiology and Infectious Diseases (DMID) grading system/NCI’s Common Terminology Criteria for Adverse Events (CTCAE) scale

19. endTB Clinical and Programmatic Guide for Patient Management with New TB Drugs. Version 3.2-www.endTB.org

20. Generic programmatic and clinical guide for the introduction of new drugs and shorter regimen for treatment of Multi/Extensively Drug-Resistant Tuberculosis-USAID/KNCV/Challenge TB-Version 0.19-April 2017

21. NCI's Common Terminology Criteria for Adverse Events (CTCAE) scale.

22. kdigo 2012 clinical practice guideline for the evaluation and management of chronic kidney disease

23. Audiometry in the management of Drug-Resistant Tuberculosis-USAID/KNCV/Challenge TB-Version 1-May 2017

24. Guidance on requirements for QTc measurement in ECG monitoring when introducing new drugs and shorter regimens for the treatment of Multi/Extensively Drug-Resistant Tuberculosis--USAID/KNCV/Challenge TB-Version 0.3-Ma

Annexes:

Annex A: Reference Laboratory services and testing capacity

Table 16: NTBLCP TB Reference Laboratories by states & testing capacity

N^0	Name of TB Reference Laboratory	Location	Zone	Type of Test Performed	Designated States
1	NRL NIMR	Yaba, Lagos State	SW	TB Culture, 1st Line & 2nd Line DST (LPA and conventional DST)	1. Lagos, Ogun (Culture, 1st& 2nd Line DST<br>2. All MDR-TB isolates from SW, SE, SS states (2nd Line DST)
2	NRL NTBLTC	Zaria, Kaduna State	NW	TB Culture, 1st Line & 2nd Line DST (LPA and conventional DST)	1. Kaduna, Zamfara, Kebbi & Sokoto (TB Culture, 1st&2nd Line DST)<br>2. All MDR-TB isolates from NW, NC, NE states (2nd Line DST)
3	TB RL UCH	Ibadan, Oyo state	SW	TB Culture, 1st Line & 2nd Line DST (LPA and Conventional DST)	1. Oyo, Osun, Ekiti, Ondo (TB culture, 1st& 2^{nd}Line DST)
4	TB RL AKTH	Kano, Kano state	NW	TB Culture, 1st Line DST (LPA and conventional DST)	1. Kano, Borno, Yobe, Jigawa, Katsina (TB culture and 1st Line DST)<br>2. Ship all MDR-TB isolates to NTBLTC Zaria for 2nd Line DST
5	TB RL Zankli	Karu, Nasarawa State	NC	TB Culture, 1st Line & 2nd Line DST (LPA and conventional DST)	1. FCT, Niger, Kwara, Kogi, Nasarawa, Benue (TB culture and 1st Line DST)<br>2. Ship all MDR-TB isolates to NTBLTC Zaria for 2nd Line DST
6	TB RL DLHMH	Calabar, Cross River State	SS	TB Culture, 1st Line Line DST (LPA and conventional DST)	1. Cross River, Anambra, Abia, Ebonyi, Akwa Ibom, Enugu (TB culture and 1st Line DST)<br>2. Ship all MDR-TB isolates to NIMR Lagos for 2nd Line DST
7	TB RL UPTH	Port Harcourt, River State	SS	TB Culture, 1st Line & 2nd Line DST (LPA and conventional DST)	1. Rivers, Delta, Bayelsa, Edo, Imo (TB culture and 1st Line DST)<br>2. Ship all MDR-TB isolates to NIMR Lagos for 2nd Line DST
8	TB RL JUTH	Jos, Plateau State	NC	TB Culture, 1st Line Line DST (LPA and conventional DST)	1. Plateau, Bauchi, Taraba, Gombe, Adamawa (TB culture and 1st Line DST)<br>2. Ship all MDR-TB isolates to NTBLTC Zaria for 2nd Line DST

Annex B: Adjustment of Anti-TB medicine dosages in patients with renal insufficiency with clearance < 30 ml/min

Table 18: Adjustment of Anti-TB medicine dosages in patients with renal insufficiency [4], [12]

Medicine	Change in dosage?	Change in frequency?	Recommended dose and frequency for patients with creatinine clearance <30 ml/min or for patients on haemodialysis (Unless otherwise indicated dose after dialysis)
Isoniazid	No change	No change	No adjustment necessary
Isoniazid High dose	No change	No change	Recommendations not available.
Rifampicin	No change	No change	No adjustment necessary
Pyrazinamide	No change	Yes	25-35 mg/kg per dose 3 times per week.
Ethambutol	No change	Yes	12-25 mg/Kg per dose 3 times per week.
Capreomycin	Yes	Yes	If Cr Cl ≤ 30 stop the injectable until creatinine returns to normal (monitor Cr weekly). Then reintroduce the injectable at the dose of 12-15 mg/kg per dose 2 or 3 times per week.
Kanamycin	Yes	Yes	
Amikacin	Yes	Yes	
Levofloxacin	No change	Yes	750-1000 mg per dose 3 times per week.
Moxifloxacin	No change	No change	No adjustment necessary.
Cicloserine	Yes	Yes	250 mg once daily, or 500 mg/dose 3 times per week
Protionamide/ Ethionamide	No change	No change	No adjustment necessary.
Para-aminosaliclic acid(PAS)	No change	No change	4g/dose, twice daily maximum dose. Use formulation of PAS that do not use sodium salt (to avoid sodium retention)
Bedaquiline	No change	No change	No dosage adjustment is required in patient with mild to moderate renal impairment (dosing not established in severe renal impairment, use with caution).
Delamanid	No change	No change	No dosage adjustment is required in patient with mild to moderate renal impairment (dosing not established in severe renal impairment, use with caution).
Linezolid	No change	No change	No adjustment necessary.
Clofazimine	No change	No change	No adjustment necessary.
Amoxicillin/ clavulanate	Yes	Yes	For creatinine clearance 10-30 ml/min dose 1000mg as amoxicillin component twice daily; for creatinine clearance <10 ml/min dose 1000mg as amoxicillin component once daily
Imipenem/ cilastatin	Yes	Yes	For creatinine clearance 20-40 ml/min dose 500 mg every 8 hours; for creatinine clearance <20 ml/min dose 500 mg every 12 hours
Meropenem	Yes	Yes	For creatinine clearance 20-40 ml/min dose 750 mg every 12 hours; for creatinine clearance <20ml/min dose 500 mg every 12 hours

AnnexC: New and repurposed drugs: Indications, adverse drug reactions, monitoring, contraindications, remarks/precautions and drug interactions.

Indications:

Bdq, Dlm and Lzd are indicated for DR-TB patients that are not eligible for STR (excluded pregnancy that need to be consider separately): Resistance to FQ/SLI ; contact with a patient with resistance to FQ/SLI; previous exposure to SLD > 1 month; intolerance to one or more medicines in STR or high risk of toxicity; risk of unfavorable outcome (extensive or advance disease: e.g., X-ray demonstrating multiple cavities, bilateral lesions, or extensive parenchymal damage or multiple system involvement). Dlm is also indicated for children > 6 years old (and > 20 Kg)

Cfz is indicated either for shorter or individualized treatment regimens.

Imp/Cln or Mpm are indicated for individualized treatment regimen when it is not possible to have 5 effective drugs to build the regimen. Imp and Mpm IV drug and is difficult to ensure a venous access for long periods of time.

Table 19: New (Bdq & Dlm) and repurposed drugs (Lzd, Cfz, Imp/Cln and Mpm)

Drug	Dosage	Adverse Drug Reactions & Monitoring	Contraindications	Remarks/precautions	Drug Interactions
**Bedaquiline (Bdq)**<br><br>100 mg tablets	**Week 1 and 2:**<br>400 mg daily<br>(4 tablets of<br>100 mg<br>7 days per week)<br><br>**Week 2 onwards**:<br>200 mg three times per week<br>(2 tablets of 100 mg three times/week)<br><br>Better absorption with food (light meal)	**More common:**<br>• Gastrointestinal (nausea, vomiting, abdominal pain);<br>• Arthralgia<br>• Headache<br><br>**Less common:**<br>• QTcFr prolongation<br>• Hepatotoxic.<br><br>**Monitoring:**<br>• ECG at baseline, week 2 then monthly (if abnormality, check ECG more frequent and request electrolytes and TSH).<br><br>• LFT monthly.	**Baseline ECG QTcF**<br>**> 500 ms** (repeated)<br>History of syncopal episode, ventricular arrhythmia or severe coronary artery disease<br><br>**Severe Hepatic failure**<br><br>**Children.**	Use with **caution:**<br>• **QTcF is > 450 ms in males or > 470 in females** (perform weekly ECG and electrolytes)<br>• **when use with other QTcFr prolonging drugs** (avoid using with Mxf, if possible)<br>• **Hypothyroidism**<br>• **Low electrolytes**<br>• **hepatic impairment**<br><br>**Pregnancy**: limited data	**Interaction with ART:**<br><br>EFV not recommended with Bdq (reduce Bdq exposure)<br><br>LPV/r: give with caution (increased Bdq level): close ECG monitoring<br><br>No interactions with NVP, NRTIs and Integrase inhibitors,

Drug	Dosage	Adverse Drug Reactions & Monitoring	Contraindications	Remarks/precautions	Drug Interactions
**Delamanid (Dlm)**<br><br>50 mg tablets	**Adults:**<br>100 mg twice daily<br>(2 tablets /12 hs)<br><br>**Children**<br>**6 to 11 years:**<br>20-34 Kg: 50 mg twice daily<br>(1 tablet/12 hs)<br>**11 to 17 years:**<br>100 mg twice daily.<br>2 tablets/12 hours.<br><br>Take with food	**More common:**<br>• Gastrointestinal (nausea, vomiting, abdominal pain).<br>• Dizziness<br><br>**Less common:**<br>• QTcFr prolongation<br><br>**Monitoring:**<br>• ECG at baseline, week 2 then monthly (if abnormality, check more frequent and request electrolytes, TSH to diagnose hypothyroidism and albumin)<br><br>• Serum Albumin at baseline. If low (<3.4g/dl) monitor more frequently albumin and ECG	**Baseline ECG QTcFr> 500 ms** (repeated)<br>History of syncopal episode, ventricular arrhythmia or severe coronary artery disease<br><br>**Serum Albumin <2.8 g/dl**<br><br>**Children < 6 years**: consult to expert Committee.	**Use with caution:**<br>• **QTcFr is > 450 ms in males or > 470 in females**<br>• (Perform weekly ECG and Electrolytes)<br>• **When use with other QTcFr prolonging drugs** (avoid using with Mxf)<br>• **Hypothyroidism**<br>• **Low electrolytes**	**Safe to administer usually with ART.**

Drug	Dosage	Adverse Drug Reactions & Monitoring	Contraindications	Remarks/precautions	Drug Interactions
**Linezolid (Lzd)**<br><br>600 mg tablets<br><br>Oral powder for suspension: 100 mg/5 ml, 240 ml bottle	**Adults:** 600 mg once daily<br><br>Add Pyridoxine 50-100 mg<br><br>**Children:**<br>10 mg/kg dose<br>If < 11 yr: thrice daily(Max 600 mg)<br>If >11 yr: twice daily (Max 600 mg)	• **Myelosuppression**<br>• (decreased level of platelets,<br>• white blood cells, and/or anemia).<br>• **Peripheral neuropathy**<br>• **Optic neuritis**<br>• **Abdominal pain**<br>• **Pancreatitis**<br>• **Lactic acidosis**<br><br>**Monitoring:**<br>• Symptoms of peripheral neuropathy monthly.<br>• Vision test monthly (or when symptoms).<br>• FBC: weekly the first month, then monthly.<br>• Amylase and Lipase at baseline and when required.	Intolerance to Lzd<br><br>**Symptoms of peripheral neuropathy**<br><br>Not recommended in pregnancy due to limited data	**Peripheral Neuropathy and optic neuritis are irreversible:** Lnz should be stopped and not re-challenged.<br><br>**Lactic acidosis:**<br>Patients who develop recurrent nausea or vomiting, unexplained acidosis, or a low bicarbonate level should receive immediate medical evaluation, including a lactic acid blood test. Lzd should be stopped and not re-challenged.	Avoid use with patients taking serotonergic agents, such as monoamine oxidase inhibitors (MAOIs), selective serotonin reuptake inhibitors (e.g. fluoxetine, paroxetine), lithium, tricyclic antidepressants, etc; it may cause serious serotonin syndrome.<br><br>Avoid concomitant use with AZT, d4T and ddI
**Clofazimine (Cfz)**<br><br>50 to 100 mg capsules	**Adults:** 200 mg once daily for 2 months followed by 100 mg daily.<br>**Children:** 1 mg/kg once daily.<br><br>Take with food	**More common:**<br>• **Skin hyperpigmentation.**<br>• **Dry skin, pruritusand rash.**<br>• **Photosensitivity.**<br>**Less common:**<br>• QTcFr prolongation.<br>• Severe abdominal symptoms.<br>• Retinopathy.<br>**Monitoring:**<br>Symptomatic monitoring	Allergy to Cfz<br><br>Not recommended in pregnancy due to limited data	Caution when use with other QTcFr prolonging drugs<br><br>Avoid in breastfeeding due to hyperpigmentation of the infant.	Using with drugs that prolong the QT interval may cause additive QT prolongation (e.g. bedaquiline, delamanid, fluoroquinolones, azole anti-fungal drugs, and others)

Drug	Dosage	Adverse Drug Reactions & Monitoring	Contraindications	Remarks/precautions	Drug Interactions
**Imipenem/ Cilastatin (Imp/Cln)**<br><br>Vials of 250 mg, 500 mg, 750 mg and 1000 mg (contain equal quantities of each drug)	1000 mg IV twice daily<br><br>Add amoxicillin/clavulanic acid (for the clavulanate component): 125 mg of clavulanate every 8 to 12 hs	**More common:**<br>• Gastrointestinal (Diarrhea, nausea, or vomiting).<br><br>**Less common:**<br>• Seizure (noted with CNS infection).<br>• Palpitations.<br>• Pseudomembranous colitis.<br><br>**Monitoring:**<br>Symptomatic monitoring	**Carbapenem intolerance;**<br><br>**Meningitis** (use meropenem rather than imipenem).<br><br>**Patients with CNS disorder.**	Little information is available regarding use in pregnancy; unknown safety during breastfeeding.	
**Meropenem (Mpm)**	**Adults:**<br>1000 mg IV every 8 hours.<br>Must be given with clavulanate (available as amoxicillin/clavulanate), 125 mg every 8–12 hours.<br>**Children:**20-40 mg/kg/dose<br>Given IV every 8 hours up to 2 g per dose.	**More common:**<br>Diarrhea, nausea or vomiting.<br>**Less common:**<br>Seizure (noted with CNS infection), but rare compared to imipenem.<br>Rarely elevated LFTs, hematologic toxicity, hypersensitivity<br><br>**Monitoring:**<br>Symptomatic monitoring	**Carbapenem intolerance.**	There is little information regarding use during pregnancy; unknown safety during breastfeeding.	
