# Supplementary material for: Clinical guidelines for managing hearing loss as a complication of drug-resistant tuberculosis treatment: an evaluation of implementation fidelity in Kano, Nigeria
Source: BMC Health Serv Res. 2022 Feb 3;22:142. doi: 10.1186/s12913-022-07536-y (PMC8812187; doi:10.1186/s12913-022-07536-y)
Supplement: Supplementary file 2 — Additional file 2. Stata output on the sample size calculation. Stata output on the sample size calculation. [file 12913_2022_7536_MOESM2_ESM.docx]

## Stata output for sample size calculation
