# Supplementary material for: Clinical guidelines for managing hearing loss as a complication of drug-resistant tuberculosis treatment: an evaluation of implementation fidelity in Kano, Nigeria
Source: BMC Health Serv Res. 2022 Feb 3;22:142. doi: 10.1186/s12913-022-07536-y (PMC8812187; doi:10.1186/s12913-022-07536-y)
Supplement: Supplementary file 4 — Additional file 4. Study questionnaire. Study questionnaire. [file 12913_2022_7536_MOESM4_ESM.doc]

## Questionnaire

Questionnaire ID: ………………………………………………………………….………….

**“SECTION A: SOCIO-DEMOGRAPHICS OF HEALTH CARE PROVIDERS**.

1. Age ______________ (in years)
2. Sex 1] Male 2] Female
3. Professional cadre: 1] Doctor 2] Nurse 3] Other, please specify ----------
4. Years working on DR-TB as Health Care Provider (in months if <1 year) ………

**SECTION B: IMPLEMENTATION FIDELITY OF HEARING LOSS MANAGEMENT GUIDELINE.**

**I. CONTENT AND COVERAGE:**

1. I always inform patients about the early symptoms of hearing loss.

1) Strongly disagree 2) Disagree 3) Neutral 4) Agree 5) Strongly agree

1. I reduce the dosing frequency of the injectables to two to three times a week in patients experiencing hearing loss.

1) Strongly disagree 2) Disagree 3) Neutral 4) Agree 5) Strongly agree

1. If patients are experiencing hearing loss, I consider switching to capreomycin (or its equivalence). 1) Strongly disagree 2) Disagree 3) Neutral 4) Agree 5) Strongly agree
2. In patients whose hearing got worse, I stop injectables if reducing dosing frequency or switching medications does not work.

1) Strongly disagree 2) Disagree 3) Neutral 4) Agree 5) Strongly agree

1. When injectables are stopped in patients whose hearing got worse, I add additional anti-TB drugs to reinforce the regimen. 1) Strongly disagree 2) Disagree 3) Neutral 4) Agree 5) Strongly agree
2. When additional anti-TB drugs are not available, and patients wants to maintain their hearing, I would continue the injectables. 1) Strongly disagree 2) Disagree 3) Neutral 4) Agree 5) Strongly agree

**II. FREQUENCY**

1. For patient on injectables, I start with a baseline audiometry at the time of enrolment. 1) Strongly disagree 2) Disagree 3) Neutral 4) Agree 5) Strongly agree
2. I perform monthly audiometry for patients on injectables after the baseline at enrolment. 1) Strongly disagree 2) Disagree 3) Neutral 4) Agree 5) Strongly agree

**III. DURATION**

1. The minimum number of audiometry test in four months is five.

1) Strongly disagree 2) Disagree 3) Neutral 4) Agree 5) Strongly agree

1. The maximum number of audiometry in eight months is nine.

1) Strongly disagree 2) Disagree 3) Neutral 4) Agree 5) Strongly agree

**SECTION C: DETERMINANTS OF IMPLEMENTATION FIDELITY.**

1. **FACILITATION STRATEGIES**
2. I was trained on the Programmatic Management of Drug-Resistant TB (PMDT) guidelines? 0) No 1) Yes
3. I received refresher training on PMDT? 0) No 1) Yes
4. I have access to a copy of the PMDT guidelines. 0) No 1) Yes
5. I have access to an audiometer. 0) No 1) Yes
6. I can get my patient to do their audiometry test on time. 0) No 1) Yes
7. I can refer my patient to see a specialist (if required) on time 0) No 1) Yes
8. **QUALITY OF DELIVERY**
9. There was good supportive supervision from my superiors during PMDT implementation.1) Strongly disagree 2) Disagree 3) Neutral 4) Agree 5) Strongly agree
10. The injectable drugs were available to me for administration to the patient at the scheduled time. 1) Strongly disagree 2) Disagree 3) Neutral 4) Agree 5) Strongly agree
11. I have an effective linkage system to an audiometer when required 1) Strongly disagree 2) Disagree 3) Neutral 4) Agree 5) Strongly agree
12. Staff were adequately sensitized through mobilization and training activities before the commencement of PMDT implementation. 1) Strongly disagree 2) Disagree 3) Neutral 4) Agree 5) Strongly agree
13. I can easily resolve ototoxicity related problems of my patients following the PMDT guidelines. 1) Strongly disagree 2) Disagree 3) Neutral 4) Agree 5) Strongly agree
14. Adequate copies of the PMDT guidelines on managing hearing loss are available during PMDT implementation. 1) Strongly disagree 2) Disagree 3) Neutral 4) Agree 5) Strongly agree
15. **INTERVENTION COMPLEXITY**
16. How would you rate the clarity of information provided in the PMDT guidelines?

1] Very vague 2] Somewhat vague 3] Neither 4] Detailed 5] Very detailed

1. How would you rate the ease of the current process of implementing PMDT guidelines?

1] Very complex 2] Somewhat complex 3] Neither

4] Somewhat simple 5] Very simple

1. How would you rate the different roles among health care providers in the PMDT guidelines?

1] Very complex 2] Somewhat complex 3] Neither 4] Somewhat simple

5] Very simple

1. **PARTICIPANTS’ RESPONSIVENESS**
2. Most DR-TB patients know their treatment process. 1) Strongly disagree 2) Disagree 3) Neutral 4) Agree 5) Strongly agree
3. Is hearing loss assessment acceptable to DR-TB patients? 1) Strongly disagree 2) Disagree 3) Neutral 4) Agree 5) Strongly agree
4. Are DR-TB patients compliant with their treatment regimen? 1) Strongly disagree 2) Disagree 3) Neutral 4) Agree 5) Strongly agree
5. Over the last six months, I have been asked questions for clarifications by DR-TB patients. 1) Strongly disagree 2) Disagree 3) Neutral

4) Agree 5) Strongly agree

**Thank you so much for your time.**

## 
